# Cost effectiveness analysis of prostate cancer screening strategies in Germany: A microsimulation study

**DOI:** 10.1002/ijc.35513

**Published:** 2025-07-16

**Authors:** Muchandifunga Trust Muchadeyi, Shuang Hao, Karla Hernandez‐Villafuerte, Shah Alam Khan, Nikolaus Becker, Agne Krilaviciute, Petra Seibold, Roman Gulati, Peter Albers, Michael Schlander, Mark Clements

**Affiliations:** ^1^ Division of Health Economics German Cancer Research Centre (DKFZ) Heidelberg Germany; ^2^ Medical Faculty Mannheim University of Heidelberg Mannheim Germany; ^3^ Department of Medical Epidemiology and Biostatistics Karolinska Institutet Stockholm Sweden; ^4^ Division of Personalized Early Detection of Prostate Cancer German Cancer Research Centre (DKFZ) Heidelberg Germany; ^5^ Division of Public Health Sciences Fred Hutchinson Cancer Center Seattle Washington USA; ^6^ Alfred Weber Institute for Economics (AWI) University of Heidelberg Heidelberg Germany

**Keywords:** cost‐effectiveness analysis, magnetic resonance imaging, organised screening, prostate cancer, prostate‐specific antigen, QALYs

## Abstract

Prostate cancer (PCa) represents a significant public health challenge in Germany, with increasing incidence and economic impact. This study assessed the cost‐effectiveness of 10 screening strategies: prostate‐specific antigen‐based risk‐adaptive screening (PSA‐RAS), with or without magnetic resonance imaging (MRI), in men starting at age 45 or 50 and stopping at 60 or 70, digital rectal examination (DRE) for ages 45–75 years, and no screening. Using a well calibrated microsimulation model (Swedish Prostata) from a statutory health insurance perspective, lifetime outcomes were evaluated, including cancer incidence, mortality, overdiagnosis, biopsies, life‐years, and quality‐adjusted life‐years (QALYs) discounted annually at 3%. Cost and utility inputs were derived from the German diagnostic‐related group schedule, fee‐for‐service catalogues, literature, and expert opinion. DRE‐only was the least cost‐effective, yielding high biopsy and overdiagnosis rates with minimal QALY gains. PSA‐RAS reduced overdiagnosis and biopsy rates, with PSA‐RAS (50–60 years) without MRI emerging as the most cost‐efficient strategy, saving approximately €1.2 million per 100,000 men compared with no screening. Extending the PSA‐RAS to 70 years improved its effectiveness in terms of QALYs. PSA‐RAS (50–70) with MRI could become cost‐effective at an increasing willingness to pay threshold or decreasing MRI cost. This study suggests the potential of PSA‐RAS to improve PCa screening in Germany. Incorporating MRI, reducing MRI cost within the screening setting, and extending screening to 70 to align with EU recommendations could improve the cost‐effectiveness of PSA‐RAS with MRI. Future research should explore the integration of MRI with ancillary tests, such as 4K‐score or risk calculators, to reduce MRI use and associated costs.

## What's New?

Prostate cancer is a significant health and economic concern in Germany. In particular, screening methods require greater optimisation to prevent overdiagnosis and unnecessary procedures and to ensure clinical benefit. Here, prostate cancer screening strategies were evaluated to determine which are most effective in terms of cost and health outcomes. Digital rectal exams alone were found to be least effective. Meanwhile, PSA‐based screening without MRI was notably cost‐effective, saving some €1.2 million per 100,000 men, relative to no screening. PSA‐based screening tailored by age, with smart use of MRI, could help lower costs and improve prostate cancer care in Germany.

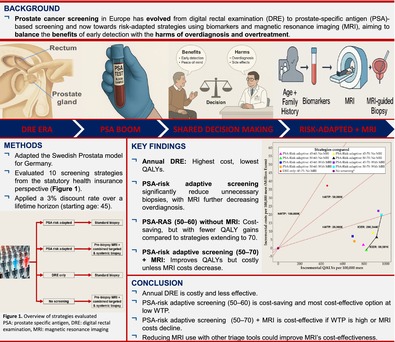

**Abbreviations**
AS: active surveillance
bpMRI: bi‐parametric magnetic resonance imaging
DGU: German Society of Urology (Deutsche Gesellschaft für Urologie)
DRE: digital rectal examination
DSA: deterministic sensitivity analysis
EAU: European Association of Urology
EU: European Union
HRQoL: health‐related quality of life
HSUV: health state utility values
hWTP: hypothetical willingness to pay threshold
ICER: incremental cost‐effectiveness ratio
IQWiG: The Institute for Quality and Efficiency in Healthcare (Institut für Qualität und Wirtschaftlichkeit im Gesundheitswesen)
ISUP: International Society of Urological Pathology
LY: life years
mpMRI: multi‐parametric magnetic resonance imaging
MRI: magnetic resonance imaging
NICE: National Institute for Health and Care Excellence
PCa: prostate cancer
PSA: prostate‐specific antigen
PSA‐RAS: prostate‐specific antigen‐based risk‐adaptive screening
QALY: quality‐adjusted life years
SBx: standard biopsies (TRUS‐guided biopsies)
TBx: MRI‐targeted biopsies
TRUS: transrectal ultrasound
WTP: willingness‐to‐pay

## 1 INTRODUCTION

Prostate cancer (PCa) significantly impacts health in Germany, with over 65,000 new cases and 15,000 fatalities in 2018.^1^ The median age at diagnosis is approximately 70 years,^2^ and its 10‐year prevalence reached 490,500 in 2020.^1^ Prevalence for PCa is expected to surpass that of breast cancer by 2030.^3^, ^4^ The economic impact is also substantial, with the total medical cost related to PCa escalating from €1.934 billion in 2015 to €3.135 billion by 2020.^5^

Traditionally, early detection of PCa has relied on digital rectal examinations (DRE), prostate‐specific antigen (PSA) tests, or both.^6^ However, concerns regarding their low sensitivity and specificity, whether used together or separately, cloud their clinical value.^6^ These drawbacks lead to overdiagnosis—identifying cancers that would remain undetected and harmless without screening—and overtreatment, causing unnecessary harm from treating such cancers.^7^ Consequently, findings from clinical trials have not supported organised PCa screening, prompting clinical guidelines to favour shared decision‐making between physicians and their patients.^8^

PCa screening is shifting toward risk‐adapted strategies that incorporate age, blood‐based biomarkers, and polygenic risk scores to determine screening ages, intervals, and biopsy recommendations.^4^, ^7^, ^9^ Incorporating magnetic resonance imaging (multiparametric [mpMRI] or bi‐parametric [bpMRI]) can guide biopsy recommendations and targeted biopsy, improving differentiation between clinically significant (Gleason score ≥7 or International Society of Uropathologists grade group [ISUP GG] ≥ 2) and insignificant cancers, potentially reducing biopsy events and overtreatment.^7^, ^8^, ^9^, ^10^ However, high costs, constrained specialised supply, implementation and quality concerns, especially in young men^11^ and lack of conclusive economic evidence hinder the widespread adoption of MRI within the screening setting.^4^

The recent inclusion of PCa in the European Commission's cancer screening recommendations^12^, ^13^ and the European Association of Urology's (EAU) endorsement of age‐related PSA risk‐adaptive screening (henceforth referred as PSA‐RAS) and MRI‐based approaches reflect a significant regional policy shift.^7^, ^9^ PSA‐RAS tailors screening by categorising individuals into risk groups based on individual PSA levels, aligning screening frequency, initiation age, and methods with their risk profiles. Many countries in Europe have initiated collaborative pilot studies of these innovative screening approaches.^14^, ^15^ The PRostate cancer Awareness and Initiative for Screening in the European Union (PRAISE‐U) (https://uroweb.org/praise-u), led by the EAU, was established to standardise screening protocols, harmonise guidelines across Member States, and facilitate data sharing to reduce prostate cancer morbidity and mortality across.^14^, ^15^, ^16^

Germany's Statutory Early Detection Program currently reimburses annual DRE from age 45 until an individual has a life expectancy of less than 10 years, and does not reimburse primary PSA testing, which has drawn increasing scrutiny.^17^ The ongoing PROBASE trial^18^, ^19^ that examines the added clinical benefit of PSA‐RAS highlighted the limited sensitivity and specificity of DRE in a young men (45–50 years).^19^ Despite the absence of a German‐specific economic evaluation, the German Society of Urology (DGU) recently proposed PSA‐RAS for men aged 45–70, starting with PSA testing, followed by stratification and, if needed, MRI and biopsy.^20^ This approach aligns with EU recommendations and the common stopping age of 69–70 years for prostate cancer screening identified in systematic reviews (10 out of 14 studies).^16^, ^21^

This study evaluated the cost‐effectiveness of PCa screening in Germany, focusing on DRE, PSA‐RAS, and MRI's potential impact. We aimed to provide additional evidence that may support Germany's health technology assessment, which emphasises added clinical benefits and health‐related quality of life (HRQoL).^22^ Existing cost‐effectiveness analyses of PSA‐RAS and MRI show varied conclusions in different settings, but such studies are conspicuously absent in Germany.^21^, ^23^, ^24^ Furthermore, no study has explored the cost‐effectiveness of PSA‐RAS and MRI, especially in the context of limited MRI resources in young men aged 45–60 and DRE.^21^, ^23^, ^24^ In Germany, the few cost‐effectiveness analyses conducted have only evaluated biomarkers as ancillary tests following a positive PSA and/or DRE.^21^, ^23^

This study addresses three key research questions:

1. Is PSA‐RAS, starting at age 50 with re‐screening intervals tailored to individual PSA levels and stopping at 60, cost‐effective? This approach is compared to (a) no screening, (b) initiating screening at age 45 and stopping at 60, and (c) DRE‐only as the existing German Statutory Early Detection Program.
2. Does incorporating MRI into PCa screening and diagnostic pathways add value? Factors considered will include reduction of overdiagnosis, improvements in HRQoL, and overall cost‐effectiveness.
3. Is extending PSA‐RAS to age 70, with or without MRI, cost‐effective? This was motivated by the DGU^20^ and widely evaluated screening stopping ages in the literature (69/70).^16^, ^21^

## 2 MATERIALS AND METHODS

A cost‐effectiveness analysis of 10 PCa screening strategies was conducted from the perspective of the German Statutory Health Insurance, including only direct healthcare costs. The base case analysis discounted future healthcare costs and health outcomes to net present value by 3%/year over a lifetime starting at the age of 45 years.

### 2.1 Study setting

Germany, with a population of approximately 85 million, is one of the most populous countries in Europe. Healthcare funding primarily comes from contributions to Statutory Health Insurance (SHI), collected by sickness funds and redistributed through the central reallocation pool—the Gesundheitsfonds—using a risk‐adjustment mechanism.^25^ Health economic evaluations in Germany are conducted on a case‐by‐case basis,^22^ which could explain the limited number of cost‐effectiveness studies specific to the Germany content. There is no universal willingness‐to‐pay (WTP) threshold; instead, evaluations must account for uncertainties, often including sensitivity analyses or comparisons with similar studies.^22^ For analyses involving multiple treatments, cost‐effectiveness ratios are depicted using efficiency frontiers.^22^

### 2.2 Competing strategies

**FIGURE 1:**
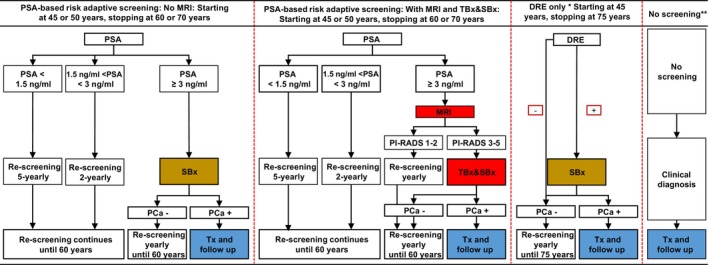
Illustrations of strategies. DRE, digital rectal examination; MRI, magnetic resonance imaging; PSA, prostate specific antigen; SBx, transrectal ultrasonography (TRUS)‐guided biopsy (standard biopsy); TBx and SBx, combined MRI targeted and standard biopsy. *DRE—only DRE is currently funded as an early PCa detection tool. **In the no screening arm, all clinically detected cases will have an MRI and combined biopsies.

Figure 1 and Table S1 in Section 1 of the Supporting Information highlights the PCa screening strategies evaluated. We evaluated 10 screening strategies: a ‘no screening’ baseline, a standalone DRE and eight PSA‐RAS strategies.

#### 2.2.1 Age‐related PSA risk‐adaptive strategies (PSA‐RAS)

The PSA‐RAS strategies were derived from the PROBASE trial^18^, ^26^—a large, prospective randomised study designed to assess the clinical effectiveness of PSA‐RAS stopping at 60—and the German Society of Urology (DGU) recommendations, which recommend extending PSA‐RAS to 70 years.

In 2014, eligible 45‐year‐old men were randomly assigned to two screening arms. Arm A received a baseline PSA test, stratifying participants into low‐ (<1.5 ng/mL), moderate‐ (1.5–3 ng/mL), and high‐risk (≥3 ng/mL) groups. Arm B deferred PSA testing until age 50 but included a family history assessment, biobanking, and optional annual DRE per Germany's Early Detection Program. Men with PSA ≥3 ng/mL in either arm were referred for mpMRI and biopsy. Rescreening was set at 2 or 5 years for moderate‐ and low‐ risk groups. The trial assesses Arm B's specificity and noninferiority for metastatic prostate cancer risk by age 60, with metastatic incidence (not mortality) as the primary endpoint. Full details are published elsewhere.^18^, ^26^

We modelled four primary strategies that closely followed PROBASE, using a PSA biopsy threshold of 3 ng/mL and rescreening intervals of 2 years for PSA 1.5–2.99 and 5 years for PSA <1.5 ng/mL (see Figure 1 and Table S1). Screening started at either age 45 or 50 and stopped at 60.

We included four additional PSA‐RAS strategies, starting at either 45 or 50 and stopping at 70, to compare with those modelled after the PROBASE trial. While current German guidelines^17^ recommend a combination of standard transrectal ultrasound (TRUS) guided systematic (SBx) and MRI‐guided targeted biopsies (TBx) [hereafter referred to as combined SBx and TBx], our model incorporated MRI as a reflex tool for biopsy decisions in all PSA‐RAS with MRI strategies. Patients with PIRADS 3–5 findings underwent combined SBx and TBx.

#### 2.2.2 Digital rectal examination (DRE) only

Given its role in Germany's current statutory early detection program, we also evaluated DRE as an independent screening strategy. We modelled annual DREs for men aged 45–75, with suspicious findings referred to SBx. We acknowledge the inherent variability and subjectivity in identifying and defining suspicious findings through DRE. Therefore, our model focused on the sensitivity and specificity values of the DRE test^27^ rather than the precise definition of ‘suspicious’ findings. The impact of these subjective interpretations was addressed in a sensitivity analysis.

#### 2.2.3 No screening

This strategy assumes no screening, with cases referred to diagnostic tests based on symptom presentation. All patients diagnosed symptomatically in the ‘no screening’ arm will incur the costs of an MRI and SBx and TBx.

#### 2.2.4 Management of symptomatic cases during screening rounds

We assumed that clinically identified cases during screening will follow the diagnostic procedures of the respective screening arm. Specifically, in PSA‐RAS strategies with MRI, combined SBx and TBx biopsies will be conducted; otherwise, standard SBx.

#### 2.2.5 Active surveillance

Active Surveillance (AS) is a conservative strategy for managing localised PCa, delaying radical treatment until necessary. It is recommended for low‐risk patients (PSA ≤10 ng/mL, Gleason score ≤6, tumour stage cT1 or cT2a, and limited tumour involvement in biopsies), and is being explored for selected Gleason 3 + 4 (7a) cases.

In our model, PCa patients are assigned to different treatment pathways, including AS, based on their Gleason score and age (Table S4, Supporting Information). AS follows German S3 and EAU guidelines, with a PSA test and DRE every 3 months for 2 years, then biannually, and periodic MRI and combined SBx and TBx biopsies. Resource use during AS assumes combined SBx and TBx biopsies, for consistency and rigorous monitoring.

### 2.3 Model selection and description

The Swedish Prostata microsimulation model (Figure S1, Section 2, Supporting Information), selected from six existing models (Table S2, Supporting Information) for its detailed staging criteria, refined Gleason grading system, open‐source availability, comprehensive documentation, and support from developers, was used.^28^

The model assumes PCa progresses through states (defined by increasing T stage and metastases) influenced by Gleason scores, age, and individual PSA levels. PSA levels increase log‐linearly, accelerating after PCa onset, influencing disease detectability and state transitions. Survival benefits associated with early diagnosis were modelled using a stage‐shift mechanism^29^ that assumes that cancers diagnosed at a later stage without screening can be detected earlier by screening and assigned cancer‐specific survival corresponding to the earlier stage, starting at the end of the lead time introduced by screening.^28^, ^30^, ^31^, ^32^, ^33^

The model details two core components: (1) longitudinal PSA growth and (2) transitions between 18 preclinical and clinical disease states, represented through mathematical and competing hazard functions to capture the underlying assumptions in PCa progression. Further details of the Swedish Prostata model have been published previously.^28^

### 2.4 Model re‐calibration and validation

The model's PCa onset and Gleason score distribution parameters were recalibrated using age‐specific PCa incidence and Gleason score distributions (2014–2019) from the German National and Saarland Cancer Clinical and Epidemiological Registries (Table S3 and Figure S2, Supporting Information). Since the registers included the influence of screening, we incorporated age‐dependent DRE test characteristics (Table S5, Supporting Information) observed in Germany.^34^ Model validation juxtaposed the observed German PCa mortality rates (2014–2019) with the model predictions.

Further details of model recalibration and validation are described in the Sections 3 and 4 of the Supporting Information.

### 2.5 Model input parameters

Table 1 details the model input parameters informed by publicly available German sources, the literature, and expert opinions.

**TABLE 1:** Model input parameters.

A. Participations rates	Estimate	DSA analysis	Notes
Screening participation rate	75%	50% or 100%	
Rescreening participation rate	95%	80% or 100%	PROBASE reported 67.9% after 5–6 years, 73.5% after 4 years, and 86.5% after 2 years, yielding an overall rate of 79.4%
Biopsy acceptance	65%	50% or 100%	PROBASE's overall biopsy acceptance was 63.6%, varying between 50.0% and 73.6% across different screening rounds.

B. DRE test characteristics				
PSA level	Sensitivity	Specificity	Distribution assumed in PSA	Notes
0–0.9	0.21	0.94	Beta	Schröder et al.^27^
1–1.9	0.24	0.92	Beta	Schröder et al.^27^
2–2.9	0.14	0.91	Beta	Schröder et al.^27^
3–3.9	0.39	0.89	Beta	Schröder et al.^27^
4–9.9	0.45	0.84	Beta	Schröder et al.^27^
≥ 10	0.52	0.83	Beta	Schröder et al.^27^
**C. Treatment distribution**	The base case analysis followed EUA guidelines, with sensitivity analysis on observed German treatment patterns detailed in the Supporting Information, Table S3 and Section 3.2, and Table S15, Section 8.			
**D. MRI test characteristics**				Hao et al.^30^

Parameter	Estimate	95% CI	Distribution assumed in PSA	Source/Notes
Specificity for mpMRI = Pr (mpMRI‐Healthy)	0.548	0.435–0.657	Log‐normal	Hao et al.^30^
Sensitivity for mpMRI (GS ≤ 6)	0.715	0.614–0.798	Log‐normal	Hao et al.^30^
Sensitivity for mpMRI (GS ≥ 7)	0.931	0.893–0.956	Log‐normal	Hao et al.^30^
Sensitivity for mpMRI‐SBx and TBx (GS ≤ 6)	0.753	0.568–0.875	Log‐normal	Hao et al.^30^
Sensitivity for mpMRI‐SBx and TBx (GS ≥ 7)	0.934	0.889–0.962	Log‐normal	Hao et al.^30^
Biopsy characteristics				Hao et al.^30^
Sensitivity for standard biopsy (GS ≤ 6)	0.860	0.824–0.889		Hao et al.^30^
Sensitivity for standard biopsy (GS ≥ 7)	0.897	0.809–0.947		Hao et al.^30^
E. Natural history				
Slope of log odds of GS = 7 at onset: beta7	0.09444911			Calibration
Slope of log odds of GS ≥8 at onset: beta8	0.2714898			Calibration
Cancer onset: Weibull onset shape	0.9273688		^c^	Calibration
Cancer onset: Weibull onset scale	187.1639			Calibration
**F. Utility estimates (measured by PORPUS‐U)**			Log‐normal	Hao et al.^32^ and duration based on Heijnsdijk et al.^36^

Health state	Estimate	95% CI	Duration	Unit
PSA test	0.99	(0.99–1.00)	1	Week
Biopsy (SBx, SBx and TBx)	0.90	(0.87–0.94)	3	Week
Cancer diagnosis	0.80	(0.75–0.85)	1	Month
Active surveillance	0.98	(0.97–0.99)	7	Year
Radical prostatectomy (part 1)	0.86	(0.76–0.96)	2	Month
Radical prostatectomy (part 2)	0.90	(0.84–0.97)	10	Month
Radiation therapy (part 1)	0.89	(0.87–0.91)	2	Month
Radiation therapy (part 2)	0.92	(0.90–0.94)	10	Month
Metastatic	0.80	NR	18	Month
Post recovery period	0.93	(0.91–0.95)	9	Year
Palliative therapy	0.68	(0.64–0.71)	12	Month
Terminal illness	0.40	NR	6	Month
**G. Utility estimates (measured by EQ‐5D)**	Sourced from Hao et al.^32^ and duration based on Heijnsdijk et al.^36^			

Health state	Estimate	95% CI	Duration	Unit
PSA test	0.99	(0.99–1.00)	1	Week
Biopsy (SBx, SBx and TBx)	0.90	(0.87–0.94)	3	Week
Cancer diagnosis	0.80	(0.75–0.85)	1	Month
Active surveillance	0.90	(0.85–0.95)	7	Year
Radical prostatectomy (part 1)	0.83	(0.73–0.91)	2	Month
Radical prostatectomy (part 2)	0.89	(0.88–0.91)	10	Month
Radiation therapy (part 1)	0.82	(0.75–0.88)	2	Month
Radiation therapy (part 2)	0.83	(0.81–0.85)	10	Month
Metastatic	0.73	(0.68–0.77)	18	Month
Post recovery period	0.86	(0.84–0.88)	9	Year
Palliative therapy	0.62	(0.58–0.66)	12	Month
Terminal illness	0.40		6	Month
**H. Background utilities**	Sourced from Janssen and Szende^35^ using EQ5D‐3 L and German Specific time trade off value set			

Age	Estimate			
0–18	1			
19–25	0.972			
26–35	0.973			
36–45	0.966			
45–55	0.945			
56–65	0.922			
66–75	0.891			
>75	0.839			
**I. Cost**	See detailed costing information in Section 5 of the Supporting Information			

Care phase	Estimate	DSA analysis	Distribution assumed in PSA	Notes/Sources
DRE test^a^	19.19	±50%	Gamma	EBM
PSA test^b^	45.67	±50%	Gamma	EBM
Multiparametric MRI^c^	521.26	±50% and €121.01	Gamma	Publicly available information online.
Biopsy (systematic/MRI targeted)	123.74	±50% and €+800	Gamma	EBM and assumptions
Staging: Assessing suspected prostate cancer	131.81		Gamma	EBM
Radical prostatectomy^e^	10,927.37		Gamma	DRG code M01B and M01A
Radiation therapy	8872.83		Gamma	Literature review and EBM
Active surveillance yearly^f^	691.71		Gamma	EBM, MRI assumptions and treatment guidelines
ADT/Chemotherapy	28,957.76	±50%	Gamma	Literature review
Post treatment follow up‐first year^g^	207.25		Gamma	Treatment guidelines and EBM valuations
Post treatment follow up‐second year onwards	79.71		Gamma	Treatment guidelines and EBM valuations
Palliative care	40,185.68		Gamma	Literature review
Terminal illness	20,092.84		Gamma	Model assumed 6 months prior to all death

Abbreviations: ADT, androgen deprivation therapy; DRE, digital rectal examination; DRG, diagnosis‐related group; DSA, deterministic sensitivity analysis; EBM, Einheitlicher Bewertungsmaßstab (German Uniform Evaluation Standard); EQ‐5D, EuroQol 5‐Dimension; GS, Gleason score; hWTP, hypothetical willingness‐to‐pay threshold; ISUP GG, International Society of Urological Pathology Gleason Grade Group; mpMRI, multiparametric magnetic resonance imaging; PORPUS‐U, Patient‐Oriented Prostate Utility Scale–Utility; PSA, prostate‐specific antigen; SBx, systematic biopsy; TBx, targeted biopsy.

^a^ A PSA test cost of €25.61, including PSA blood sample and lab analysis is added if DRE is positive.

^b^ PSA lab analysis cost is based on private market pricing.

^c^ mpMRI is not currently reimbursed by the SHI. Based on a crude estimate from publicly available information online https://www.primomedico.com/en/treatment/prostate‐mri/.

^d^ Based on expert opinion and a thorough review of the existing literature, we did not include prophylactic antibiotics, aligning our approach with the evolving guidelines and recommendations that are moving away from this practice.

^e^ Complication rates and duration of hospital stay, data source: Dissertation, University of Hamburg. https://ediss.sub.uni‐hamburg.de/handle/ediss/9176.

^f^ During active surveillance, biopsies should be taken every twelve to 18 months for the first three years, and later every three years if the findings are stable. We assumed combined SBx and TBx during active surveillance.

^g^ In asymptomatic patients after curative therapy, the determination of the serum PSA value should be used for follow‐up. Imaging techniques should only be used if therapeutic measures are possible and/or symptoms exist.

Schröder and colleagues^27^ provided DRE specificity and sensitivity. Hao and colleagues^30^ informed the sensitivity and specificity for MRI and combined SBx and TBx based on the STHLM3‐MRI trial.^10^

The fee for service (Einheitlicher Bewertungsmaßstab [EBM]) reimbursement catalogue for outpatient services and the German Diagnosis‐Related Groups (DRGs) for inpatient services, expert opinion, and previous studies were used to estimate costs (Section 5 of the Supporting Information, including Tables S6–S9 and Figure S5, Supporting Information). All costs are in 2023 Euro (€). Additional cost data, including those for androgen deprivation therapy (ADT), chemotherapy, palliative care, and informal care, were derived from previous studies and adjusted to 2023 levels using the Consumer Price Index.

Janssen and Szende^35^ provided age‐specific background utility values consistent with German societal norms. The base case analysis employed the PORPUS‐U disease‐specific health state utility values (HSUVs) derived from Hao and colleagues.^32^ The health state durations came from Heijnsdijk and colleagues.^36^ HRQoL reductions were calculated using a multiplicative model where each health state utility was multiplied by its age‐specific background utility.

### 2.6 Model outcomes

The total number of DRE or PSA screens, MRI events, biopsies, clinically and screen‐detected PCa, PCa‐related deaths, life‐years (LYs), quality adjusted life years (QALYs), overdiagnosis, and accumulated healthcare costs for each strategy were reported per 100,000 life histories from 10 million men, simulated from 35 to death.

### 2.7 Cost‐effectiveness analysis

Incremental cost‐effectiveness ratios (ICERs) were calculated only for efficient strategies^37^ (Section 6, Supporting Information). To be cost‐effective, a strategy must be clinically effective and either reduce costs or meet predefined ICER benchmarks when more costly (Figure S6, Supporting Information). Without standardised WTP thresholds in Germany, we used hypothetical thresholds of €20,000; €50,000 and €100,000 per QALY gained, strictly for evaluative purposes and not as suggested standards (Section 6, Supporting Information).

### 2.8 Sensitivity analyses

#### 2.8.1 Deterministic sensitivity analyses

Deterministic sensitivity analyses (DSA) evaluated key variables, including HSUVs, discount rates (0 and 5%), screening intervals, starting and stopping ages, and cost parameters.

MRI cost dynamics were considered, accounting for potential reductions due to economies of scale within screening programmes or shifting from mpMRI to bpMRI. Scenario 1 examined the 10 strategies with reduced MRI costs (€120 per scan). Scenario 2 excluded rescreening for low‐risk patients (PSA <1.5 ng/mL) using a biopsy threshold of PSA ≥4 ng/mL (Section 7.1.3, Supporting Information), while Scenario 3 excluded rescreening for low‐risk patients (PSA <1.5 ng/mL) while maintaining a biopsy threshold of PSA <3 ng/mL (Section 7.1.3, Supporting Information). We also analysed the impact of replacing PORPUS‐U with EQ‐5D HSUVs (Section 7.1.2, Supporting Information).

#### 2.8.2 Probabilistic sensitivity analysis

The probabilistic sensitivity analysis, conducted over 1000 iterations of a cohort of one million men, explored the impact of the joint distribution of test characteristics, costs, and HSUVs. The sampling ranges and distributions of these parameters are listed in Table 1. The results of these analyses are depicted using model outcome tables, cost‐effectiveness planes, and acceptability curves.

## 3 RESULTS

### 3.1 Calibration and Validation

Visually, the recalibrated model fit well, representing the increasing PCa incidence between 40 and 75 years of age in Germany. The model also successfully predicted age‐specific PCa‐specific mortality in Germany, notably for those over 65 years, where the disease burden is substantial (Figure S4, Panels A and B, Supporting Information).

### 3.2 Costs and health outcomes

Panel A of Table 2 details projected health outcomes for 100,000 men followed from age 45. Without screening, 10,474 males per 100,000 developed PCa. The intensive DRE‐only screening strategy detected the most cases, correlating with increased LYs and QALYs, and the lowest mortality. However, this was associated with the highest number of screen‐initiated biopsies (151,125 per 100,000) and substantial overdiagnosis, with 2468 of the 12,942 PCa cases detected per 100,000 men likely undetectable without screening.

**TABLE 2:** Predicted outcomes and health economic results from age 45 years by strategy.

	PSA‐Risk‐adaptive: 50–60: No MRI	No screening	PSA‐Risk‐adaptive: 45–60: No MRI	PSA‐Risk‐adaptive: 50–60: With MRI	PSA‐Risk‐adaptive: 50–70: No MRI	PSA‐Risk‐adaptive: 45–60: With MRI	PSA‐Risk‐adaptive: 45–70: No MRI	PSA‐Risk‐adaptive: 50–70: With MRI	PSA‐Risk‐adaptive: 45–70: With MRI	DRE only: 45–75: No MRI
A. Outcomes per 100,000 men										
Number of DRE test										1,872,494
Number of PSA tests	219,869	0	284,427	221,082	367,051	285,572	424,222	371,180	428,132	0
Number of MRI events	0	0	0	13,486	0	13,000	0	51,798	49,367	0
Total number of biopsies	29,235	19,672	28,965	20,714	62,169	20,701	60,230	26,069	25,792	161,126
Number of clinically‐initiated biopsies	16,508	19,672	16,683	17,312	12,985	17,437	13,334	14,073	14,365	10,001
Number of screen‐initiated biopsies	12,727	NA	12,282	3402	49,184	3264	46,897	11,995	11,427	151,125
Absolute reduction in screen‐initiated biopsies^a^	138,398	NA	138,843	147,723	101,941	147,861	104,228	139,130	139,698	Reference
Percentage reduction in screen‐initiated biopsies (%)^a^	92%	NA	92%	98%	67%	98%	69%	92%	92%	Reference
Absolute reduction in screen‐initiated biopsies^b^	9325	NA	9018	Reference 1	37,189	Reference 2	35,470	Reference 3	Reference 4	NA
Percentage reduction in screen‐initiated biopsies (%)^b^	73%	NA	73%	Reference 1	76%	Reference 2	76%	Reference 3	Reference 4	NA
Number of diagnosed PCa	10,788	10,474	10,771	10,675	11,714	10,665	11,654	11,377	11,330	12,942
Number of clinically diagnosed PCa	8805	10,474	8897	9223	6930	9289	7115	7492	7647	5329
Number of screen diagnosed PCa	1983	0	1874	1452	4784	1376	4539	3885	3683	7613
Number of localised and GS <7	3840	3628	3828	3732	4414	3727	4378	4098	4072	5115
Number of localised and GS = 7	3509	3416	3504	3505	3778	3500	3759	3766	3748	4123
Number of localised and GS >7	3439	3430	3439	3438	3522	3438	3518	3513	3510	3704
Number of metastatic PCa diagnosed	1731	2018	1745	1795	1373	1805	1405	1448	1476	1036
Estimated overdiagnosed PCa	314	Reference	297	202	1240	191	1180	903	857	2468
Absolute reduction in overdiagnosed PCa^a^	2154	NA	2170	2266	1228	2277	1287	1565	1611	Reference
Percentage reduction in overdiagnosed PCa (%)^a^	87%	NA	88%	92%	50%	92%	52%	63%	65%	Reference
Absolute reduction in overdiagnosed PCa^b^	112	NA	106	Reference 1	337	Reference 2	324	Reference 3	Reference 4	NA
Percentage reduction in overdiagnosed PCa (%)^b^	36%	NA	36%	Reference 1	27%	Reference 2	27%	Reference 3	Reference 4	NA
Prostate cancer deaths	3321	3657	3336	3409	3010	3417	3041	3117	3142	2718
LY, undiscounted	3,462,414	3,458,500	3,462,290	3,461,555	3,464,970	3,461,489	3,464,724	3,464,031	3,463,836	3,467,354
LY, discounted at 3%	2,092,631	2,091,200	2,092,592	2,092,335	2,093,452	2,092,317	2,093,375	2,093,138	2,093,078	2,094,220
QALYs, undiscounted	3,122,116	3,119,139	3,122,016	3,121,523	3,123,221	3,121,468	3,123,069	3,122,885	3,122,757	3,123,430
QALYs, discounted at 3%	1,913,406	1,912,587	1,913,379	1,913,295	1,913,502	1,913,279	1,913,470	1,913,548	1,913,518	1,913,043
QALYs, discounted at 5%	1,474,928	1,474,640	1,474,915	1,474,922	1,474,859	1,474,914	1,474,850	1,474,964	1,474,952	1,474,339
B. Costs (M€) per 100,000 men										
Healthcare perspective, undiscounted	405	426	409	417	408	420	411	436	438	443
Healthcare perspective, discounted at 3%	165	166	168	170	172	173	175	186	188	203
Healthcare perspective, discounted at 5%	97	94	100	101	104	104	107	113	115	131
C. Incremental changes per 100,000 men										
Prostate cancer deaths	335	Reference	321	248	647	240	616	540	514	939
LY, discounted at 3%	1430	Reference	1392	1134	2252	1117	2174	1938	1877	3019
QALYs, discounted at 3%	820	Reference	793	708	915	692	884	961	931	456
Healthcare perspective (M€), discounted at 3%	−1,2	Reference	1.9	4.1	6.5	7.1	9.3	20.0	22.3	37.4
Impact of HRQOL assumptions	0.573	Reference	0.569	0.624	0.406	0.620	0.406	0.496	0.496	0.151
D. Incremental cost‐effectiveness ratios (ICERs)										
Compared to no screening										
Cost/Death averted	Dominant	Reference	6040	16,653	10,038	29,728	15,011	36,979	43,323	39,842
Cost/LY gained	Dominant	Reference	1392	3646	2883	6381	4255	10,300	11,872	12,384
Cost/QALY gained	Dominant	Reference	2444	5843	7095	10,296	10,472	20,762	23,940	81,987
ICERs on efficient frontier										
Cost/Death averted	First on the frontier	Dominated	Dominated	Dominated	24,695	Dominated	Dominated	Dominated	Dominated	105,889
Cost/LY gained	First on the frontier	Dominated	Dominated	Dominated	9351	Dominated	Dominated	Dominated	Dominated	40,269
Cost/QALY gained	First on the frontier	Dominated	Dominated	Dominated	80,581	Dominated	Dominated	290,344	Dominated	Dominated

*Note*: A. Health outcomes for 100,000 men; B. Cumulative direct medical cost from the statutory health insurance perspective per 100,000 men; C. ICERs (€ per QALY gained). Overdiagnosis refers to detecti*o*n of PCa through screening that would not have been detected during the patient's lifetime without screening. Columns are arranged in increasing order of health care perspective cost discounted at 3%. With MRI means MRI is used to inform biopsy decisions followed by combined systemic (SBx) and targeted (TBx) biopsies. Strategies without MRI, ‘No MRI’, do not include MRI use to triage biopsy decision, suspicious cases are followed by systemic biopsies.

Abbreviations: DRE, digital rectal examination; GS: Gleason score; LY: life years; MRI, magnetic resonance imaging; PCa: prostate cancer; PSA, prostate specific antigen; QALYs: quality‐adjusted life‐years.

^a^ The comparison was made with the reference strategy.

^b^ Each strategy was compared with a closely related strategy with the same age range but differing in MRI usage, indicated as Reference 1 to Reference 4. For example, PSA‐risk adaptive screening (ages 50‐60) without MRI was compared with the same age range that included MRI.

Transitioning from DRE‐only to PSA‐RAS reduced the number of screen‐initiated biopsies and the overdiagnosis rates by 95% and 90%, respectively. Notably, incorporating MRI triaging into PSA‐RAS further reduced these numbers by 76% and 36%, respectively (References 1 and 2, Table 2, panel A).

However, all MRI‐based strategies cost more than the non‐MRI strategies (Table 2, panel B). Due to higher biopsy, overdiagnosis, and treatment expenses, DRE screening costs the most.

### 3.3 Cost‐effectiveness

**FIGURE 2:**
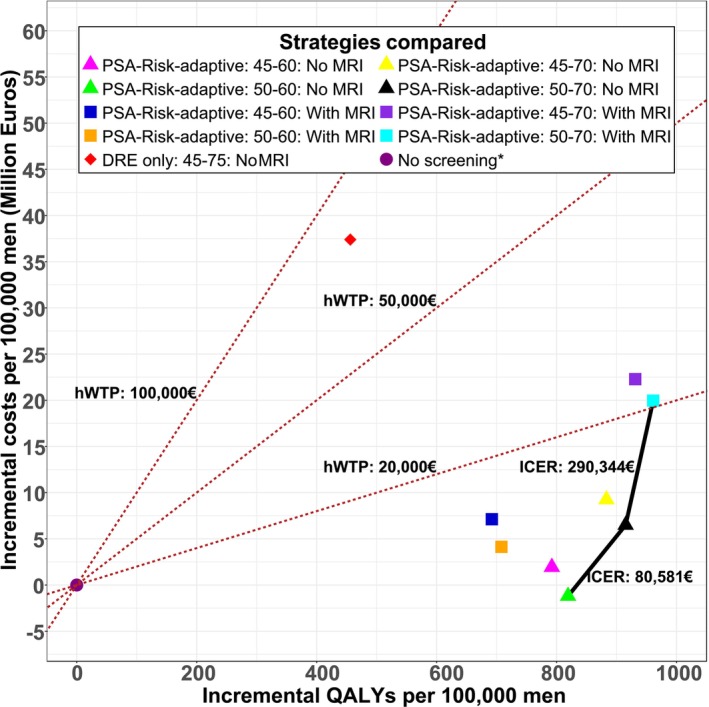
Cost‐effectiveness plane: Base case analysis for Germany. Costs and QALYs are discounted by 3%. Differences in costs and QALYs comparing screening strategies with no screening; health‐state utility value set measured by PORPUS‐U. Solid line indicates cost‐efficiency frontier, with the lowest cost strategy, PSA‐Risk‐adaptive: 50–60: No MRI, being the first strategy on the frontier. With MRI means MRI is used to inform biopsy decisions followed by combined systemic (SBx) and targeted (TBx) biopsies. Strategies without MRI, ‘No MRI’, do not include MRI use to triage biopsy decisions; suspicious cases are followed by systemic biopsies. *In the base case analysis, all symptomatically detected cancers were assumed to be further investigated using MRI imaging followed by combined biopsies; the red dotted lines represent the various hypothetical willingness to pay thresholds (hWTP) used as the decision rule to interpret the cost‐effectiveness ratios. MRI, magnetic resonance imaging; PSA, prostate‐specific antigen; QALYs, quality‐adjusted life‐years; DRE, digital rectal examination.

Figure 2 showcases the cost‐effectiveness of 10 base case strategies evaluated on the CE plane. When screening stopped at age 60, MRI‐based strategies significantly reduced screen‐initiated biopsies by 63%–75% (Table 2, panel A). PSA‐RAS (50–60 years) without MRI was the dominant strategy, saving approximately €1.2 million per 100,000 men compared to no screening (Table 2, panel C).

When screening was extended to age 70, PSA‐RAS strategies showed greater benefits compared to screening stopped at 60. The MRI‐based PSA‐RAS (50–70 years) strategy achieved highest QALY gains, positioning it on the efficient frontier with an ICER of €290,344 per QALY gained compared to PSA‐RAS (50–70 years) without MRI (Table 2, panel D). The latter strategy, also on the efficient frontier, demonstrated an ICER of €80,581 per QALY gained compared to PSA‐RAS (50–60 years) without MRI.

### 3.4 Sensitivity analysis

#### 3.4.1 Deterministic sensitivity analysis

**FIGURE 3:**
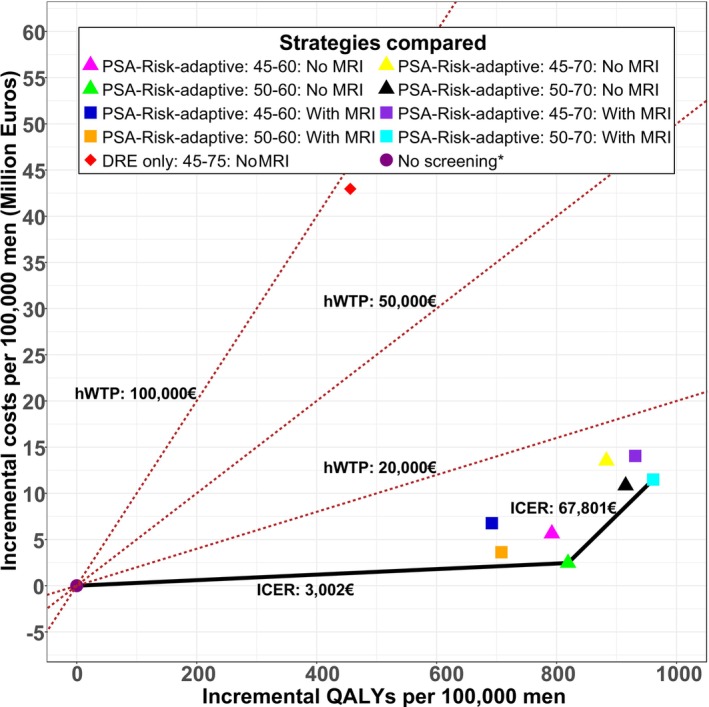
Results of deterministic sensitivity analysis: Assessing the impact of reducing MRI cost to 120€ per MRI scan (Scenario 1). Solid line indicates cost‐efficiency frontier, with the lowest cost strategy, PSA‐Risk‐adaptive: 50–60: No MRI, being the only strategy on the frontier. With MRI means MRI is used to inform biopsy decisions followed by combined systemic (SBx) and targeted (TBx) biopsies. Strategies without MRI, ‘No MRI’, do not include MRI use to triage biopsy decision; suspicious cases are followed by systemic biopsies. *In the base case analysis, all symptomatically detected cancers were assumed to be further investigated using MRI imaging followed by combined biopsies. The red dotted lines represent the various hypothetical willingness to pay threshold (hWTP) used as the decision rule to interpret the cost‐effectiveness ratios. DRE, digital rectal examination; MRI, magnetic resonance imaging; PSA, prostate specific antigen; QALYs, quality‐adjusted life‐years.

Figure 3 demonstrates the improved ICER of PSA‐RAS (50–70 years) with MRI, which decreased to €67,801 per QALY when the MRI costs were reduced from €500 used in the base case to €120 per scan in Scenario 1.

Increasing ADT/chemotherapy costs reduced the incremental cost of all screening strategies compared to no screening, further improving the ICER of PSA‐RAS (50–70) without MRI relative to PSA‐RAS (50–60) with MRI (Figure S7, Supporting Information).

Using EQ‐5D values, as recommended by the National Institute for Health and Care Excellence (NICE), only PSA‐RAS (50–60) with or without MRI remained on the efficient frontier and eligible for reimbursement based on the government's WTP threshold (Figure S8 and Table S10, Supporting Information). Notably, PORPUS‐U values are generally higher than EQ‐5D. Further details on the DSA results, including scenario 2 and 3 which reduced the intensity of screening for low risk groups (Figure S9 and Tables S11 and S12), lowering DRE stopping age to 70 years (Figure S10 and Tables S13 and S14), using a discount rate of 5% (Figure S11), adjusting treatment distributions to observed patterns (Figures S3 and S12 and Table S15), reducing MRI cost by 50% (Figure S13), and varying participation rates (Tables S16 and S17) are provided in Section 7 of the Supporting Information.

As a summary, Figure S14 (Supporting Information) compares the 10 base case strategies with selected variations explored in the DSA, highlighting the trade‐offs between cost and effectiveness across different screening approaches.

#### 3.4.2 Probabilistic sensitivity analysis

**FIGURE 4:**
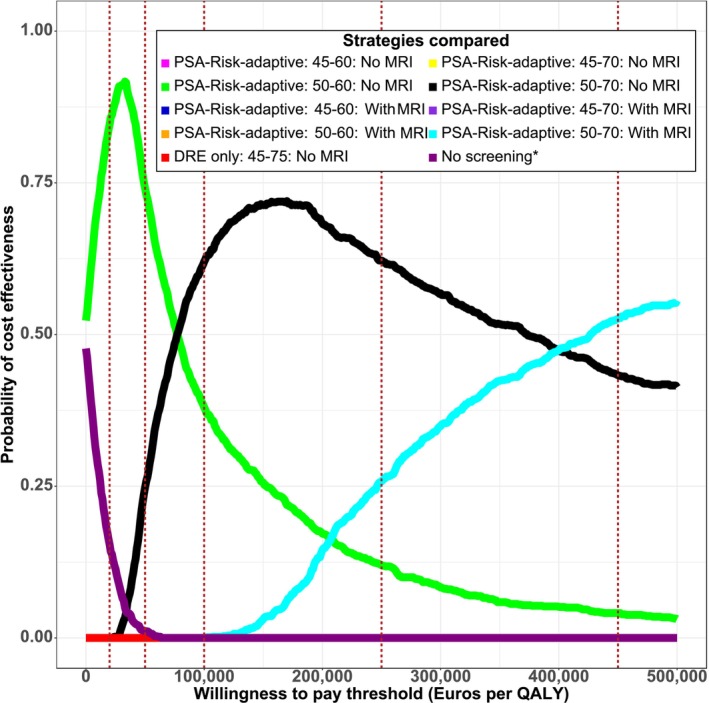
Cost‐effectiveness acceptability curves: Results of probabilistic sensitivity analysis. All 10 strategies included—lines not appearing on the curve exhibited zero probability of being cost‐effective within the willingness to pay threshold displayed: Horizontal red dashed line indicates the arbitrary cost‐effectiveness threshold (k) of 20,000€, 50,000€, 100,000€, 250,000€, and 450,000€ per QALY gained. With MRI means MRI is used to inform biopsy decisions followed by combined systemic (SBx) and targeted (TBx) biopsies. Strategies without MRI, ‘No MRI’, do not include MRI use to triage biopsy decisions; suspicious cases are followed by systemic biopsies. *In the base case analysis, all symptomatically detected cancers were assumed to be further investigated using MRI imaging followed by combined biopsies. DRE, digital rectal examination; MRI, magnetic resonance imaging; PSA, prostate specific antigen; QALYs, quality‐adjusted life‐years.

Figure 4 illustrates the likelihood of each of the 10 base‐case strategies being cost‐effective across various hypothetical WTP thresholds on the cost‐effectiveness acceptability curve. PSA‐RAS (50–60 years) without MRI has the highest probability of cost‐effectiveness at WTP thresholds below €75,000 per QALY. Between €75,000 and €400,000 per QALY, PSA‐RAS (50–70 years) without MRI is most likely cost‐effective. Beyond €400,000 per QALY, PSA‐RAS (50–70 years) with MRI is the most cost‐effective strategy, with a probability exceeding 50%.

Figure S15 in the Supporting Information explores the same probabilistic analysis, assuming a reduced mean MRI cost of €120 (from 500€) to reflect potential cost reductions. In this scenario, PSA‐RAS (50–60 years) without MRI retains the highest probability of cost‐effectiveness at WTP thresholds below €75,000 per QALY. Notably, the likelihood of PSA‐RAS (50–70 years), with MRI being cost‐effective, increases significantly, exceeding a 60% probability at WTP thresholds below €100,000 per QALY.

## 4 DISCUSSION

Germany introduced its Statutory Early Detection Program in 1971, offering annual DRE to men aged 45 and older. This made Germany one of the first countries globally to implement a cancer detection initiative.^38^ Despite widespread opportunistic PSA testing in Europe, organised PSA screening exists only in Lithuania and Sweden, while it remains unfunded in Germany.^4^, ^16^, ^39^ Germany has arguably been cautious in adopting modern screening technologies.^22^ Recently, the European Commission recommended updated cancer screening strategies, including prostate cancer screening, urging Germany to consider revising its early cancer detection approach.^20^, ^38^ This study evaluated the cost‐effectiveness of PSA‐RAS with or without MRI within the context of annual DRE‐only and the absence of screening, providing an economic rationale for considering smarter and more innovative screening methods.

### 4.1 Main findings

Of the 10 strategies evaluated in the base case, DRE‐only was costly and prone to high overdiagnosis, overtreatment, and reduced HRQoL. Consistent with the level A clinical evidence,^8^, ^10^, ^40^ PSA‐RAS with MRI significantly reduced biopsy events and overdiagnosis. However, the high frequency of use of MRI and high MRI costs make PSA‐RAS with MRI less cost‐effective compared with PSA‐RAS without MRI, if screening is limited to 60 years.

In contrast, extending screening to age 70 as recommended by the EU guidelines and GDU favoured MRI‐based strategies, adding approximately 80 QALYs per 100,000 men over no screening (in addition to the base 813 QALYs per 100,000, when screening stops at 60 years), with an ICER of approximately €290,000 per QALY compared to the same strategy without MRI. Extending screening to 70 years while reducing MRI costs to €120—assuming potential reimbursement within the screening setting—further reduced the ICER for PSA‐RAS with MRI (ages 50–70) to approximately €68,000 per QALY. Compared to no screening, assuming exceptionally high ADT and chemotherapy costs further improved the cost savings stemming from PSA‐RAS without MRI.

Another key takeaway from the DSA is that the choice of HSUVs significantly impacts cost‐effectiveness conclusions. Using EQ‐5D, as recommended by NICE, would have led to different screening recommendations, highlighting the need for careful utility selection in economic evaluations.

### 4.2 Comparison with previous studies

To our knowledge, this is the first study to compare the cost‐effectiveness of DRE and PSA‐RAS, with or without MRI. Compared with no screening, focusing on a younger population (ages 45–60) yielded lower ICERs for PSA‐RAS, ranging from being dominant for PSA‐RAS (50–60) without MRI to €10,296–€13,438 per QALY for PSA‐RAS (45–60) with MRI. These ICERs are lower than those reported in previous studies,^31^, ^32^, ^33^, ^36^, ^41^ likely due to the limited screening duration (e.g., 50–60 years) and risk stratification, which reduced screening events, overdiagnosis, and overall costs.

Similar trends were observed in a previous review,^24^ noting the cost‐effectiveness of a single screen at 55 years and annual to biannual screening starting at 55–59/63 years,^36^ whereas most PSA strategies extended to 70 or 75 were dominated.^41^

An unexpected finding in our study is that PSA‐RAS with MRI in young men (45–60) was dominated by strategies without MRI, despite reductions in biopsy rates and overdiagnosis. This contrasts with the conclusions from Vynckier and colleagues,^21^ who reported PSA‐RAS with MRI could be cost‐effective. This discrepancy may stem from differences in screening algorithms, PSA thresholds, population characteristics, MRI costs, and costing methods. Notably, Vynckier and colleagues^21^ did not consider tailored rescreening based on current PSA values for younger men aged 50–60. Their study^21^ which included 14 European studies, of which only two^31^, ^42^ were risk‐adapted with pre‐biopsy MRI starting at ages 55 to 70—highlights significant heterogeneity in screening algorithms, further highlighting the need for caution when generalising the conclusions regarding the cost‐effectiveness of PSA‐RAS followed by MRI.

In our study, the modestly lower QALY gains for MRI strategies when screening is limited to 60 years may result from the limited benefit of targeted biopsies in younger men with smaller prostate volumes. The model assumes that ISUP grade group 1 cancer has a 15‐year cause‐specific survival of approximately 0.95; therefore, there is some long‐term harm from such cancers. Consequently, delays in diagnosing such cancers, although reducing unnecessary biopsies, may offset the benefits due to the potential harms of delayed treatment and increased mortality risk.^40^

When screening is extended to age 70, aligning with EU recommendations, DGU guidelines, and literature norms,^16^, ^21^ our findings support those from previous studies suggesting that PSA‐RAS (50–70), with MRI, could be cost‐effective at higher WTP thresholds. However, the ICER in our analysis (~290,000 per QALY gained) is higher than those reported in other settings,^30^, ^32^, ^33^ likely because of the high MRI costs (€500 per scan). Expert input indicated that MRI costs in the PROBASE trial were approximately €400 per scan and could decrease further in the screening context. Reducing MRI costs to €120 per scan in our model significantly improved the ICER from approximately €290,000 per QALY to €68,000 per QALY, aligning more closely with findings from Swedish^30^, ^32^ and UK studies.^33^ For reference, in the Netherlands, one of the few countries alongside the UK that use explicit WTP thresholds, the acceptable range is €20,000 to €80,000 per QALY, with a weighting factor of up to 4 for interventions targeting the most severe conditions.^43^

### 4.3 Policy relevance

Given these observations, a pertinent question persists: what is a sustainable approach for PCa screening in Germany? Foregoing population‐based organised screening while allowing shared decision‐making might lead to unnecessary repeated testing, biopsies, overdiagnosis, and loss of HRQoL, particularly in wealthy, educated, young, low‐risk men and those with limited life expectancy.^39^, ^44^

Similarly, intensive DRE, which is unpopular^19^ due to its invasive nature, inherent variability in the interpretation of findings^45^ and considerable patient discomfort,^6^ resulted in increased costs for less QALY gains.^6^, ^19^ Our results suggest that transitioning from DRE to PSA‐RAS may reduce these costs and enhance the HRQoL.

The clinical significance of MRI in triaging biopsy decisions must be considered. Our study aligns with high‐level evidence from clinical trials^10^, ^46^ and systematic reviews,^8^ showing that MRI significantly reduces the number of biopsy procedures—by approximately 75%—and enhances diagnostic precision by more accurately differentiating between likely indolent and potentially aggressive forms of prostate cancer (PCa), thus reducing overdiagnosis. Nonetheless, substantial MRI costs and HRQoL assumptions could impact its cost‐effectiveness. Similar concerns exist regarding MRI reporting quality and interpretation variability.^47^

While PSA‐RAS (50–60) without MRI dominated no screening, strategies extending screening to the age of 70 years, with or without MRI, achieve greater QALY gains, albeit at marginally higher costs. PSA‐RAS (50–70), with MRI, may be more cost‐effective as WTP increases. Furthermore, the value of MRI extends beyond cost considerations. Patient preferences, acceptability, and attitudes towards screening pathways must also be factored into decision‐making.

Structured interviews conducted by Merriel and colleagues^48^ found that MRI was generally acceptable to patients (45–80‐year age groups), despite concerns about burdens such as travel, parking, and out‐of‐pocket expenses. Similarly, Krilaviciute and colleagues^49^ and a previous systematic review^8^ reported that incorporating MRI into the diagnostic pathway in Germany improved adherence to subsequent screening rounds and increased biopsy rates, potentially enhancing the overall effectiveness of PCa screening.

### 4.4 Study strengths

Our study leveraged a well‐validated microsimulation natural history model previously applied in other European settings.^30^, ^31^, ^32^, ^33^, ^50^ The model recalibration process benefitted immensely from a collaborative environment involving clinicians, epidemiologists from the PROBASE trial, and model developers from the Karolinska Institutet. This collaboration ensured that the model conceptualisation, including the framing of strategies, was aligned with the German context, and that model implementation was thoroughly checked and validated at each phase of the project.

Although this study would have benefited from access to real‐world health insurance claims data, the cost analysis was comprehensive. It included detailed cost components derived from German diagnostic‐related groups (DRG), fee for service (EBM) codes, and clinical guidelines. Importantly, a practising urologist validated the outpatient cost codes and parameters to enhance accuracy.

Furthermore, the pre‐biopsy MRI and combined SBx and TBx characteristics were based on robust, high‐level evidence, including a large sample of 1532 participants with PSA levels ≥3 ng/mL who were randomised to either a standard biopsy group or an experimental group receiving MRI followed by targeted and standard biopsies.^10^, ^30^

### 4.5 Study limitations

While our model is well calibrated and validated, it has limitations typical of such models. We simplified the DRE testing assumptions and lacked Germany‐specific PSA testing data during the model recalibration. The DRE test characteristics were based on an older study and most reports pertain to clinical rather than screening cohorts, possibly limiting their generalisability.

Excluding opportunistic PSA testing might underestimate the cost implications without altering the clinical outcomes. Most PSA testing occurs with annual DREs recommended by the Early Cancer Detection Program, often outside the recommended age range of 55 to 70 years.^51^ Research indicates that individuals outside this age range may be less likely to benefit from screening.^44^ Due to a lack of robust data to model opportunistic screening, we used a no‐screening approach for comparability with previous studies, which also predominantly used the no‐screening approach as the base comparison.

Our study focused on a limited set of strategies primarily aligned with the PROBASE trials,^18^ the German Society of Urology's recommendations,^20^ and the PRAISE‐U consortium's re‐screening and biopsy thresholds.^16^ For future work, one could more fully explore the policy landscape, including varying re‐screening and biopsy thresholds.

Another key limitation is the relevance and applicability of HSUVs to the German context. The Institute for Quality and Efficiency in Health Care (IQWiG) Methods document recommends that QALY calculations use HSUVs based on patient valuations. Utilities from the general population may be used only if they align closely with patient valuations.^22^ Ideally, HSUVs should be derived from the country in which the decisions are made.

Although the PORPUS‐U is a widely used disease‐specific instrument that comprehensively captures the critical dimensions of PCa (including social relationships) and has been validated in multiple settings,^52^ most studies informing the HSUVs in this study were conducted in Canada (five out of nine studies), with none from Germany. Studies have shown that HSUVs are sensitive to cultural and contextual factors, raising questions about the applicability of these values to the German setting.^53^

Our study does not represent recent cost increases in mPCa costs, especially with recent advances in anti‐hormonal treatments such as apalutamide and darolutamide, which may increase mPCa cost^50^—albeit without improving quality of life (dominated).^54^ Nonetheless, our findings (Figures S7, Supporting Information) suggest that accounting for increased mPCa costs could render screening more cost effective. The focus of cost‐effectiveness analyses on overall survival and HRQoL (QALY) may not fully capture the broader clinical value of screening (e.g., prevention of metastatic disease).

Finally, Germany does not apply WTP thresholds to funding decisions for public health initiatives, making it challenging to establish firm decision rules based on the conventional cost‐effectiveness paradigm. Instead, arbitrary WTP thresholds were used in this study based on standards from other settings. Even in settings with defined WTP thresholds, these are typically expressed as cost per QALY rather than cost per death averted or cost per life‐year gained and may vary by intervention type. For example, the WTP for cancer and severe cases can be higher; NICE, for instance, has a separate funding mechanism for such cases, as their ICERs often exceed the standard acceptable limits. Currently, there is no specific WTP threshold for screening interventions.^43^

### 4.6 Recommendations for future research

Given our findings, further research on the cost‐effectiveness of MRI screening is warranted. This entails exploring combined risk assessment tools including blood biomarkers, PSA, PSA density, and DRE as preliminary ‘triaging tests’ prior to MRI, and the optimal ages to use MRI, aiming to decrease the frequency and cost associated with the procedure. Additionally, research on the health state utility values in PCa is necessary, focusing on how emerging technologies affect screening, biopsy, and treatment outcomes. Artificial intelligence is already gaining traction in radiation oncology—significantly improving biopsy accuracy^55^ —and warrants further health economic evaluation.

## 5 CONCLUSIONS

Taken together, our study, focusing on the German context, suggests that annual DRE, as a standalone, is more costly and less effective than PSA‐RAS for ages 45 to 70. Offering young men (age 50–60) PSA‐RAS without MRI could be cost effective. PSA‐RAS with MRI could be cost‐effective if screening is extended to 70, WTP is high or MRI cost is reduced. Future studies should investigate the effective integration of MRI in screening, including the use of ancillary tests to reduce MRI events and costs, and to potentially improve the cost‐effectiveness of MRI. While the German HTA emphasises clinical added benefit when informing reimbursement decisions, particularly overall survival, incorporating cost‐effectiveness analysis can support such clinical evidence.

## AUTHOR CONTRIBUTIONS

**Muchandifunga Trust Muchadeyi:** Conceptualization; methodology; data curation; formal analysis; investigation; writing – original draft; writing – review and editing; visualization; project administration; validation; software. **Shuang Hao:** Conceptualization; methodology; data curation; investigation; formal analysis; writing – review and editing; visualization; validation. **Karla Hernandez‐Villafuerte:** Conceptualization; methodology; data curation; investigation; formal analysis; writing – review and editing; validation; visualization. **Shah Alam Khan:** Conceptualization; data curation; formal analysis; writing – review and editing; validation. **Nikolaus Becker:** Conceptualization; data curation; writing – review and editing. **Agne Krilaviciute:** Conceptualization; data curation; writing – review and editing. **Petra Seibold:** Conceptualization; data curation; writing – review and editing. **Roman Gulati:** Data curation; writing – review and editing. **Peter Albers:** Conceptualization; data curation; writing – review and editing; project administration. **Michael Schlander:** Conceptualization; data curation; writing – review and editing; validation; supervision; project administration; methodology; funding acquisition; resources. **Mark Clements:** Conceptualization; supervision; methodology; software; data curation; investigation; validation; visualization; formal analysis; project administration; writing – review and editing; funding acquisition.

## FUNDING INFORMATION

The project was part of the first author's PhD thesis, fully funded by the German Cancer Research Centre. Research visits to the Karolinska Institutet (Sweden) were partially funded by the Swedish Cancer Society (Cancerfonden; No. CAN21/1512) and the Swedish Research Council (Vetenskapsrådet; No. 2022‐00684 and the Swedish eScience Research Centre).

## CONFLICT OF INTEREST STATEMENT

The authors declare no conflicts of interest.

## Supporting information


**Data S1.** Supporting Information.

## Data Availability

All processed data used in the analysis is included in the Supporting Information. Raw data can be requested directly from the respective cancer registries. The Swedish Prostata and analytical codes can be accessed on GitHub at https://github.com/mclements/prostata and https://github.com/mclements/prostata/blob/develop/inst/doc/Trust.zip. Further information is available from the corresponding author upon request.
