# Supplementary material for: Cost effectiveness analysis of prostate cancer screening strategies in Germany: A microsimulation study
Source: Int J Cancer. 2025 Jul 16;157(8):1662–79. doi: 10.1002/ijc.35513 (PMC12375846; doi:10.1002/ijc.35513)
Supplement: Supplementary file 1 — Data S1. Supporting Information. [file IJC-157-1662-s001.pdf]

ELECTRONIC SUPPLEMENTARY MATERIAL (ESM)

Title: Cost effectiveness analysis of prostate cancer screening strategies in Germany: A microsimulation study

Authors

Muchandifunga Trust. Muchadeyi; Shuang Hao; Karla Hernandez-Villafuerte; Shah Alam Khan; Nikolaus Becker; Agne Krilaviciute; Petra Seibold; Roman Gulati; Peter Albers; Michael Schlander; Mark Clements

Table of contents

Supplementary materials and methods ... 4
1 Competing strategies ... 4
2 Model selection ... 5
3 Model recalibration and validation ... 9
3.1 Gleason score distribution ... 10
3.2 Treatment distribution ... 11
3.3 Calibration methods ... 13
3.4 Model validation ... 13
4 Model calibration results and validation results ... 13
5 Resource use and cost estimation ... 15
5.1 Targeted literature review ... 17
5.2 Data extraction ... 22
5.3 Expert opinion ... 22
5.4 Special consideration to the costing approach ... 32
5.4.1 Consultation or assessment cost ... 32
5.4.2 Metastatic prostate cancer treatment cost ... 32
5.4.3 Palliative and terminal care cost ... 35
6 Cost-effectiveness analysis ... 37
Supplementary results ... 39
7 Sensitivity analysis ... 39
7.1 Deterministic sensitivity analysis ... 39
7.1.1 Impact of varying the cost of mPCa care ... 39
7.1.2 Impact of health states utility values ... 41
7.1.3 Scenario 2 and 3: Different PSA biopsy threshold and rescreening intervals: ... 44
7.1.4 Effects of varying stopping ages for DRE and PSA risk adaptive screening ... 48
7.1.5 Discounting cost and health at 5% instead of 3% ... 51
7.1.6 Impact of treatment patterns ... 52
7.1.7 Impact reducing MRI cost from €500 ... 54
7.1.8 Summary of the results of the DSA ... 55
8 Supplementary references ... 60

List of supplementary figures

Figure S.1: Schematic representation of the Swedish Prostata microsimulation model 8
Figure S.2: Age-specific Gleason score and ISUP grade distribution 11
Figure S.3: Treatment distribution by Gleason category in Germany (2014–2020). 12
Figure S.4: Simulated vs. observed age-specific prostate cancer rates in Germany. 14
Figure S.5: Resource use along the prostate cancer care continuum 16
Figure S.6: The cost-effectiveness plane and the decision rules 38
Figure S.7: DSA: Impact of uncertainty on cost of ADT/chemotherapy 40
Figure S.8: DSA: Impact of using EQ5D health states utility values. 42
Figure S.9: DSA: Panel A: Scenario 2 and Panel B: Scenario 3 45
Figure S.10: DSA: Impact of Lowering Stopping Age in DRE-Only Strategy (**75 to 70**)............... 49
Figure S.11: DSA: Impact of varying the discount rates on cost and QALYs 51
Figure S.12: DSA: Impact of using observed treatment distributions form the Saarland registry. 53
Figure S.13: DSA: Impact of reducing MRI cost by 50%.. 54
Figure S.14: DSA: Ten base case strategies compared with selected variations during the DSA 56
Figure S.15: CEAC: Results of probabilistic sensitivity analysis: mean MRI cost=120€ 57

List of supplementary tables

Table S.1: Overview of the competing strategies... 4
Table S.2: Overview of currently existing prostate cancer models 5
Table S.3: Treatment and Gleason Scores, 2014–2020 (Saarland & Hamburg) 10
Table S.4: Base case analysis treatment distribution 12
Table S.5: Assumed probabilities for the 1st DRE test by age group 13
Table S.6: Costing and cost-effectiveness analysis studies specific for Germany setting. 18
Table S.7: Resources and unit costs characterization 23
Table S.8: Conservative estimated treatment cost for metastatic prostate cancer in Germany.......... 34
Table S.9: Estimation of treatment cost for bone metastasis 34
Table S.10: Model outcomes by strategy from age 45: Use of EQ-5D values 43
Table S.11: Model outcomes by strategy from age 45: Scenario 2 46
Table S.12 Model outcomes by strategy from age 45: Scenario 3.. 47
Table S.13: Selected outcomes comparing DRE only stopping at 75 or 70................................... 48
Table S.14: Model outcomes by strategy from age 45: Reduce DRE stopping age to 70 years 50
Table S.15: Treatment Distribution by Gleason Score from the Saarland Registry, 2014-2019 52
Table S.16: Model outcomes by strategy from age 45: Using 100% screening participation rate..... 58
Table S.17: Model outcomes by strategy from age 45: Using 50% screening participation rate....... 59

Supplementary materials and methods

1 Competing strategies

Table S.1: Overview of the competing strategies

Strategy Name	Starting and stopping age	Screening test (test positivity threshold)	MRI triaging	Biopsy type (SBx or TBx/SBx)	MRI for symptomatic diagnosis**
PSA-Risk-adaptive: 45-60: No MRI	45-60	PSA ≥ 3ng/ml	No	SBx	No
PSA-Risk-adaptive: 45-70 No MRI	45-70	PSA ≥ 3ng/ml	No	SBx	No
PSA-Risk-adaptive: 50-60: No MRI	50-60	PSA ≥ 3ng/ml	No	SBx	No
PSA-Risk-adaptive: 50-70: No MRI	50-70	PSA ≥ 3ng/ml	No	SBx	No
PSA-Risk-adaptive: 45-60: With MRI	45-60	PSA ≥ 3ng/ml	Yes	TBx&SBx	Yes
PSA-Risk-adaptive: 45-70: With MRI	45-70	PSA ≥ 3ng/ml	Yes	TBx&SBx	Yes
PSA-Risk-adaptive: 50-60: With MRI	50-60	PSA ≥ 3ng/ml	Yes	TBx&SBx	Yes
PSA-Risk-adaptive: 50-70: With MRI	50-70	PSA ≥ 3ng/ml	Yes	TBx&SBx	Yes
DRE only: 45-75: No MRI	45-75	DRE only*	No	SBx	No
No screening***	NA	NA	No	TBx&SBx	Yes

PSA: Prostate specific antigen; SBx: transrectal ultrasonography (TRUS) guided biopsy (standard biopsy); MRI: magnetic resonance imaging; TBx&SBx: combined MRI targeted and standard biopsy; DRE: Digital rectal examination; * DRE is currently the only funded early PCa detection tool in Germany. ** If MRI for symptomatic diagnosis is "Yes", this means all cases detected through symptoms and not screening during the screening period, will also be referred to MRI. *** In the base case analysis, all symptomatically detected cancers were assumed to be further investigated using MRI imaging followed by combined biopsies

2 Model selection

Table S.2: Overview of currently existing prostate cancer models

	ERASMUS MC (PROSTATE)	PCSIM (FHCRC)	PSAPC (FHCRC)	SCANS (MICHIGAN)	ONCOTYROL PCOP	SWEDISH PROSTATA
	**CISNET MODELS**				Adapted ERASMUS Prostate	Adapted the PSAPC (FHCRC)
**Research group**	Erasmus medical centre	Fred Hutchinson Cancer Research Center	Fred Hutchinson Cancer Research Centre	University of Michigan	UMIT-University for Health Sciences, Medical Informatics and Technology	Karolinska Institutet
**Modelled age groups (years)**	55-74	50-84	>55	Not specified		55-69
**Description of health states**	18 preclinical detectable states:<br>¬ **T-stage (T1, T2 and T3)**<br>¬ Gleason grade <7, =7, and >7<br>¬ **Gleason grade is determined at prostate cancer onset and does not change.**	9 disease states based on the AUS system[4]:	Not clearly specified:<br>¬ **Low-moderate (Gleason score 2-7)**<br>¬ **high (Gleason score 8-10)**<br>¬ **Gleason grade is determined at prostate cancer onset and does not change.**	No states defined, the model is an event or process based defined by a series of closed form mathematical functions:	Same as ERASMUS MC (PROSTATE)	18 preclinical detectable states:<br>¬ **T-stage (T1-2, T3-4 and Metastatic)**<br>¬ Gleason grade ≤6, 7and ≥8<br>¬ **Gleason grade is determined at prostate cancer onset and does not change.**
**PSA growth assumptions**	**No assumptions on PSA growth**	PSA trajectories with different growth rate depending on whether one has cancer or not.	PSA trajectories with different growth rate depending on whether one has cancer or not.	**No assumptions on PSA growth**	**No assumptions on PSA growth**	PSA trajectories with different growth rate depending on whether one has cancer or not

**Screening tests**	¬PSA based screening<br>¬PSA> 4ng/ml<br>¬No DRE modelled	¬PSA based screening<br>¬PSA> 4ng/ml<br>¬No DRE modelled	¬PSA based screening<br>¬PSA> 4ng/ml<br>¬**DRE detections modelled**	PSA screening	¬PSA based screening<br>¬PSA> 3ng/ml<br>¬No DRE modelled	¬PSA based screening<br>¬PSA> 3ng/ml<br>¬No DRE modelled
**Treatments**	¬Radical prostatectomy (RP)<br>¬Radiation therapy (RT)<br>¬Active surveillance (AS)	**No treatments modelled**	¬Conservative management (None)<br>¬Radical prostatectomy<br>¬Radiation therapy<br>¬Radiation therapy combined with hormones	**No treatments modelled**	¬Radical prostatectomy (RP)<br>¬Radiation therapy (RT)<br>¬Androgen deprivation therapy (ADT)<br>**Did not consider treatment heterogeneity**	¬Radical prostatectomy (RP)<br>¬Radiation therapy (RT)<br>¬Active surveillance (AS)<br>¬Androgen deprivation therapy (ADT)
**Model outcomes**	¬Disease characteristics<br>¬Stage and grade distribution<br>¬Overall survival<br>¬Life years and QALYs<br>¬Disease specific and all-cause mortality<br>¬Screening effects<br>¬False and True positives<br>¬Total biopsies performed<br>¬Unnecessary biopsies	¬Overall survival<br>¬Life year gains<br>¬QALYs<br>¬All cause specific mortality<br>¬Overdiagnosis	¬Overall survival<br>¬Life year gains<br>¬QALYs<br>¬All cause specific mortality<br>¬Overdiagnosis	¬Overall survival<br>¬Life year gains<br>¬QALYs<br>¬All cause specific mortality<br>¬Overdiagnosis	¬Overall survival<br>¬Life year gains<br>¬QALYs<br>¬All cause specific mortality	¬Disease characteristics<br>¬Stage and grade distribution<br>¬Overall survival<br>¬Life years and QALYs<br>¬Disease specific and all-cause mortality<br>¬Screening effects<br>¬False and True positives<br>¬False and True negatives<br>¬Total biopsies performed<br>¬Unnecessary biopsies

¬ Overdiagnosis and overtreatment	¬ Overdiagnosis and overtreatment

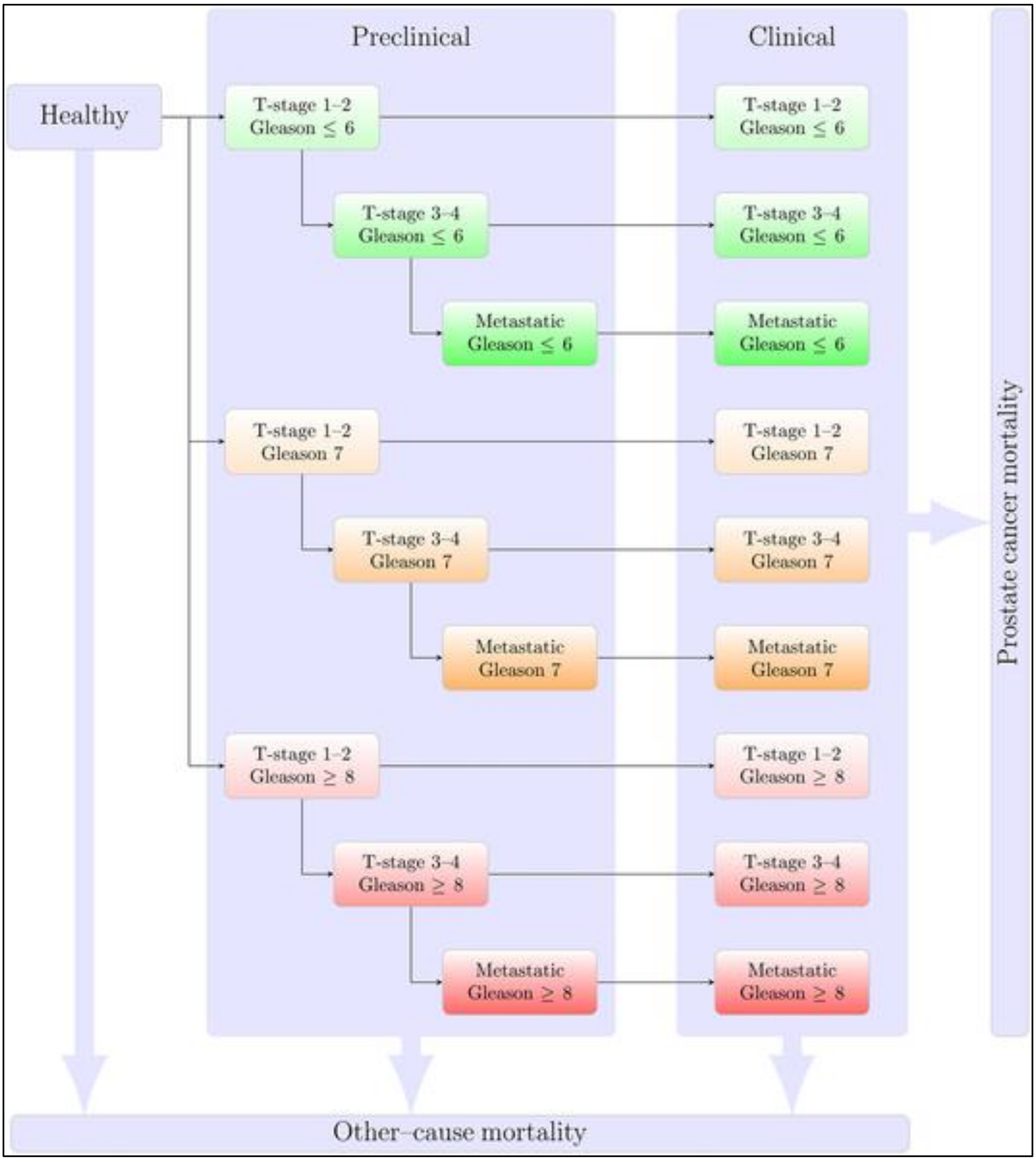

Figure S.1: Schematic representation of the Swedish Prostata microsimulation model
Source: Karlsson et al. (2019) [1]
The figure represents the prostate cancer natural history reflecting disease onset, progression and survival in the absence of screening. Note that there are no arrows connecting different tumour grades (Gleason score) as Gleason score de-differentiation is not allowed in the model. Upon diagnosis people receive various treatment depending on age, and Gleason score including radical prostatectomy, radiation therapy, active surveillance and androgen deprivation or chemotherapy. T-stage: tumour stage

3 Model recalibration and validation

Natural history models play a crucial role in assessing prostate cancer screening strategies and optimizing healthcare resources. These models are vital for simultaneously evaluating clinical and cost-effectiveness across different screening interventions. To ensure accuracy and relevance, it is essential to calibrate and validate these models carefully for the specific context, ensuring reliable, context-specific predictions.

Description of data sources

Modelling the natural history of prostate cancer requires tracing the disease progression from its onset to death. Inadvertently, certain stages of this progression such as the exact onset of cancer are unobservable, yet understanding these stages is crucial. We rely on observable epidemiological trends like incidences, stage distributions and prevalence to infer these hidden processes. These trends act as calibration targets for mathematical models, helping infer the unobserved processes.

Unsurprisingly, clinical and epidemiological cancer registry data are indispensable in informing health economic model parameters. They serve a dual purpose: as direct inputs and as calibration targets to refine both observable and unobservable parameters, thereby enhancing model performance.

Under German law, anonymized electronic medical records can be used for research without patient consent or ethical committee approval [2]. Capitalising on this provision, we accessed data from three prominent cancer registries in Germany:

- German Federal Cancer Registry–Robert Koch-Institut (RKI)
- Hamburg State Cancer Registry
- Saarland Sate Cancer Registry

The German Federal Cancer Registry consolidates data from 1,127,953 prostate cancer cases across all 16 federal states, dating from January 15, 1970, to December 15, 2019. This dataset, however, has limitations, notably in missing information on T and M stages [3]. For instance, missing prostate cancer T stage data averaged 33% from 1970 to 2019, 31% from 2010 to 2019, and 32% from 2014 to 2019. Similarly, missing data for M stages averaged 44% from 1970 to 2019, 41% from 2010 to 2019, and 40% from 2014 to 2019.

The federal registry's lack of critical information on treatments, Gleason scores, and PSA values was supplemented by state registries. We turned to the Hamburg and Saarland State registries for detailed information on Gleason scores, PSA values, and treatments. Due to the substantial missing Gleason scores in the Hamburg registry, we focused our Gleason score and treatment distribution analysis on the Saarland Cancer Registry, which contained data on 6,207 prostate cancer cases diagnosed between January 15, 2014, and December 15, 2020 (see Table S.3).

Table S.3: Treatment and Gleason Scores, 2014–2020 (Saarland & Hamburg)

Year of diagnosis	Hamburg Cancer Registry (N = 6297)					Saarland Cancer Registry (N = 6207)				
	Received at least one treatment	Treatment unknown	Total	Treatment unknown %	Gleason score missing %	Received at least one treatment	Treatment unknown	Total	Treatment unknown %	Gleason score missing %
**2014**	443	350	793	44.1%	95.8%	101	656	757	86.70%	16.1%
**2015**	558	312	870	35.9%	94.5%	201	556	757	73.40%	12.5%
**2016**	569	337	906	37.2%	91.6%	481	406	887	45.80%	11.5%
**2017**	600	305	905	33.7%	77.1%	595	288	883	32.60%	9.1%
**2018**	661	328	989	33.2%	32.8%	617	362	979	37.00%	8.1%
**2019**	629	312	941	33.2%	15.7%	699	323	1022	31.60%	6.8%
**2020**	585	308	893	34.5%	12.3%	636	286	922	31.00%	9.3%
**Totals**	4045	2252	6297	35.8%	58.6%	3330	2877	6207	46.40%	10.2%

3.1 Gleason score distribution

Figure S.2, panel A presents the Germany age-specific (50-80 years) Gleason score distribution. A notable observation is that German prostate cancer patients predominantly fall into the intermediate Gleason score category (Gleason score = 7). This contrasts with Sweden, where patients are more frequently found in the lower (Gleason score ≤ 6) category [1].

Figure S.2, panel B illustrates the corresponding distribution of the ISUP Gleason Grades, highlighting the distinction between ISUP grade 2 (GS 3+4 = 7) and ISUP grade 3 (GS 4+3 = 7). In Germany, a higher concentration of patients is observed in ISUP GG 3 (4+3), considered less favourable compared to ISUP GG2 (3+4).

These findings underscore significant differences in population characteristics between Germany and Sweden, further emphasizing the necessity for model recalibration. We attribute these disparities primarily to the different diagnostic approaches in the two countries. In Germany, DRE is the primary screening tool, whereas Sweden largely relies on PSA-based testing.

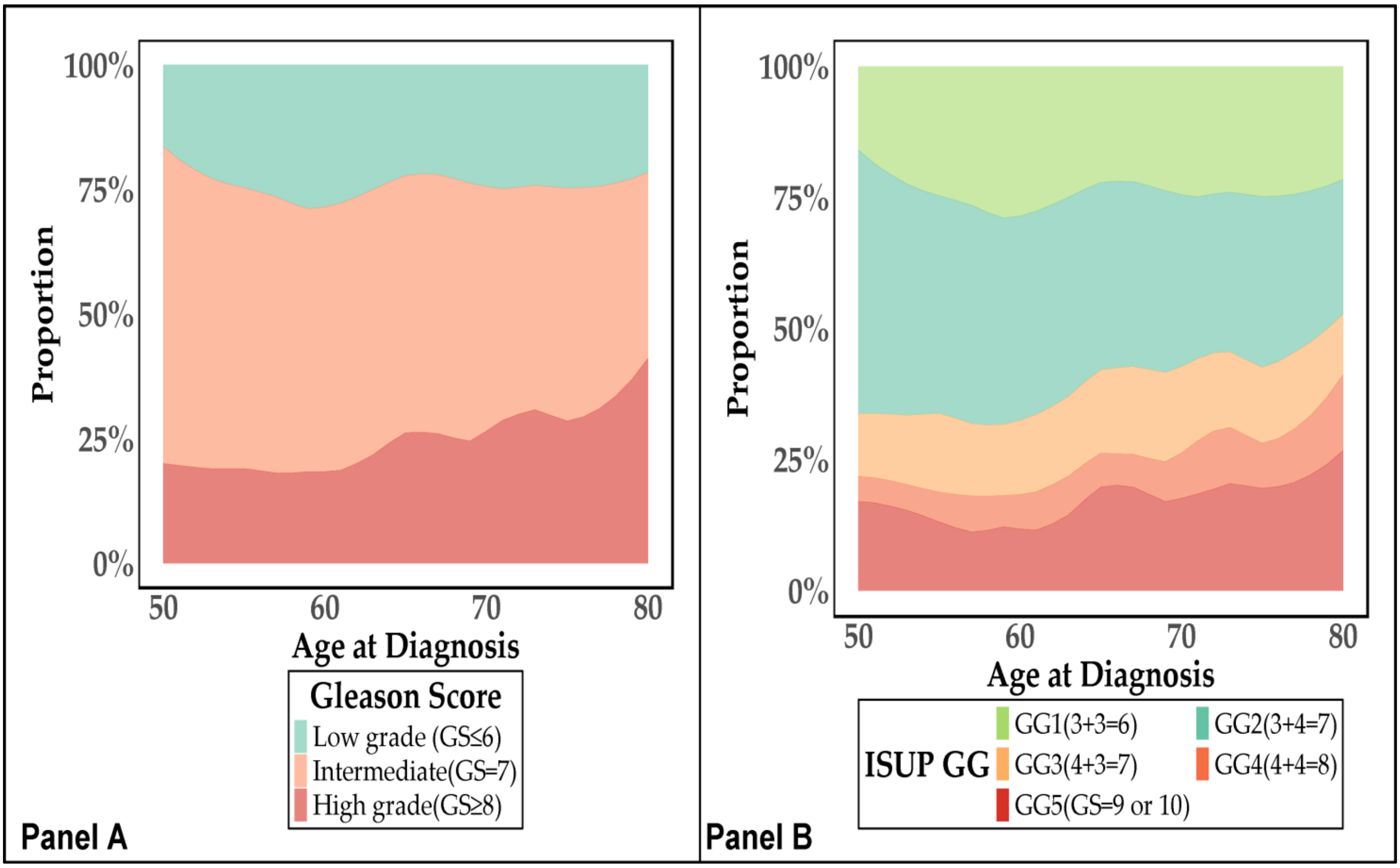

Figure S.2: Age-specific Gleason score and ISUP grade distribution
Panel A: Gleason score distribution by age (50–80 years). **Panel B:** ISUP Gleason Grade (GG) distribution by age (50–80 years
Data represent prostate cancer cases in Germany from 2014 to 2020. GS: Gleason score. GS: Gleason score

3.2 Treatment distribution

Figure S.3 illustrates the Germany age-specific treatment distribution by Gleason score category in, as observed in the Saarland clinical and epidemiological cancer registry. Contrary to recent guidelines that favour more conservative approaches like Active Surveillance (AS) and Watchful Waiting (WW), and delayed radical treatments like radical prostatectomy and radiation therapy, real-world evidence from Germany suggests a different practice. Notably, there is a significant inclination towards radical prostatectomy in younger men with favourable Gleason scores (≤ 6) and in older men with reduced life expectancy, where the benefits of such aggressive treatments are uncertain. More importantly, this trend differs significantly from the Swedish approach [1], where conservative management is more prevalent, further emphasizing the need for context-specific models.

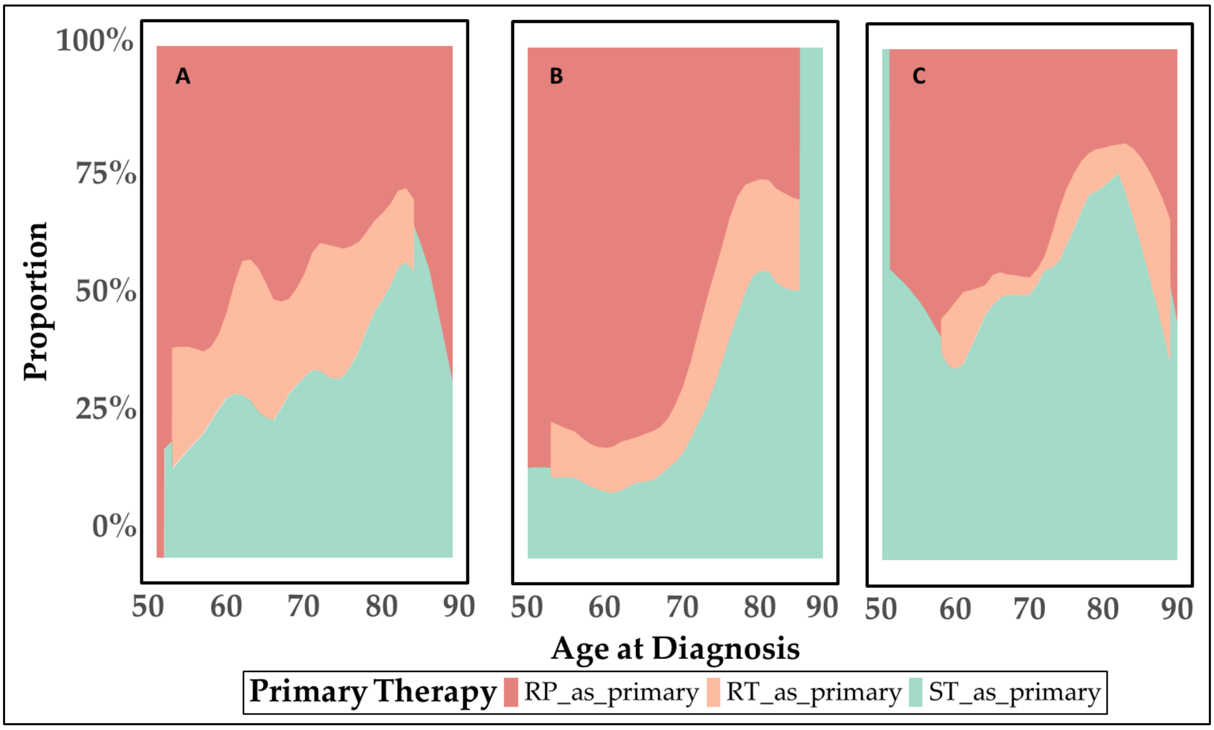

Figure S.3: Treatment distribution by Gleason category in Germany (2014–2020).
Relative frequency of primary treatments (N = 4,045) by Gleason score category: **Panel A:** Low grade (Gleason score ≤ 6); **Panel B:** Intermediate grade (Gleason score = 7) and **Panel C:** High grade (Gleason score ≥ 8)
RP: Radical prostatectomy; RT: radiation therapy and ST: Systemic therapy
Notably, there is a significant inclination towards radical prostatectomy in men with favourable and intermediate Gleason scores (≤ 6 and ==7) across all age groups

In our model, treatment options include Radical Prostatectomy (RP), Radiotherapy (RT), Conservative Management (CM) [Active Surveillance (AS)], and Androgen Deprivation Therapy/Chemotherapy (ADT/Chemo). ***Given the quality of the available data, we decided to align our base case treatment distribution with current guidelines and reserved the observed treatment distribution for the deterministic sensitivity analysis***. prostatectomy

Table S.4 outlines the treatment distribution for the base case analysis, aligned with EUA guidelines, striking a balance between data constraints and the need for a contextually relevant, rigorous model

Table S.4: Base case analysis treatment distribution

Age	Gleason score	Conservative management	Radical prostatectomy	Radiation therapy
**50-74**	≤6	1	0	0
**50-74**	7	0	0,5	0,5
**50-74**	≥8	0	0,5	0,5
**≥75**	≤6	1	0	0
**≥75**	7	1	0	0
**≥75**	≥8	1	0	0

3.3 Calibration methods

The model was calibrated to German age-specific prostate cancer incidences (2014 to 2019) from the National Cancer registry supplied by RKI and age specific Gleason score distribution from the Saarland Cancer registry data.

Since prostate cancer detection is already widely used in Germany, we assumed DRE testing at various intensities by age derived from a nationally representative study on early prostate cancer detection in Germany. Table S.5 shows the age wise probabilities for having a 1st DRE. Thereafter an individual could be tested annually or every 2.5 years with probabilities 0,254, and 0,134 respectively, else could never be retested again.

Table S.5: Assumed probabilities for the 1st DRE test by age group

**Age group**	**45-49**	**50-54**	**55-59**	**60-64**	**65-70**
**Cumulative probability of DRE testing**	0,489	0,622	0,713	0,782	0,819

Model calibration was done in a single step. Using the age-wise prostate cancer incidences queried from the national cancer registry for periods 2014 to 2019, a Poisson likelihood estimation was used to fit for the log shape and scalar parameters to the Weibull distribution describing the rate of cancer onset. Concurrently, a multinomial likelihood estimation was employed to fit for the individual specific slope parameter for Gleason Score (GS) distribution (log odds of G7 and log odds of G $\geq$ 8 at onset). Refer to Karlsson et al 2019 for the mathematical narration of the cancer onset and Gleason score distributions.

3.4 Model validation

The model's validation involved comparing observed prostate cancer mortality rates in Germany from 2014 to 2019 with the predicted mortality rates from the model.

4 Model calibration results and validation results

Figure S.4 Panel A depicts the results of the model predictions juxtaposed to observed Germany age-specific prostate cancer incidences in Germany from 2014 to 2019. A visual inspection reveals that the model effectively forecasts the observed trend of increasing prostate cancer incidences from age 40 to 75 among the German population. Panel B displays the results of this comparison, showing the model's predictions to observed German mortality data. Upon visual inspection, the model appears to accurately predict age-specific mortality rates for individuals older than 65 in Germany, where the majority of prostate cancer deaths typically occur

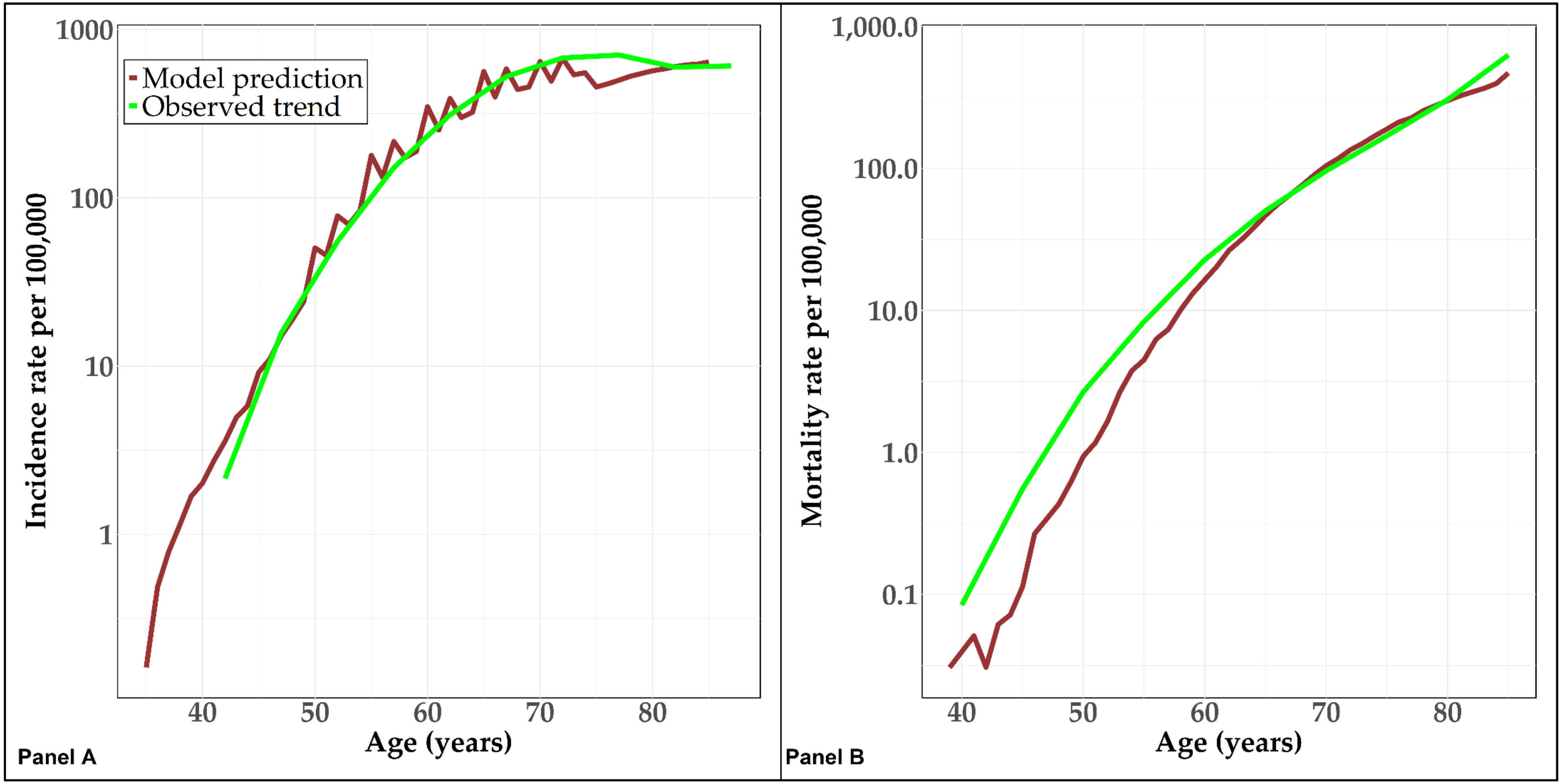

Figure S.4: Simulated vs. observed age-specific prostate cancer rates in Germany.
Panel A: Incidence rates. **Panel B:** Mortality rates.
Model prediction represents simulated incidence or mortality rates under current practices. Observed trends are based on data from the Robert Koch-Institut (RKI), 2014–2019) for incidence or *The Global Cancer Observatory* (2014–2019) for mortality.

5 Resource use and cost estimation

In exploring the nuances of cost in health economics, we reflect on AC Blades' perspective.

> *"Costs are not immutable facts lying ripe in the field waiting merely to be garnered, or even selectively winnowed, by diligent clerical officers. The nature of the decision for which a study is a managerial input will dictate the likely opportunity costs"*

Costing for this study, conducted from the Germany Statutory Health Insurance (SHI) perspective, included all direct costs incurred along the prostate cancer care continuum (from screening to death), valued in 2023 currency**.** ***Indirect costs such as co-payments, out-of-pocket expenses, informal care, and productivity losses were excluded***.

Figure S.5 adapted from Hao and colleagues [4], provided the analytical framework for conducting the costing along the prostate cancer care continuum. The direct healthcare cost estimation process involved:

1. Identifying resources used throughout prostate cancer care.
2. Quantifying resource usage.
3. Assigning unit prices to resources.
4. Calculating total costs for each resource as model inputs:

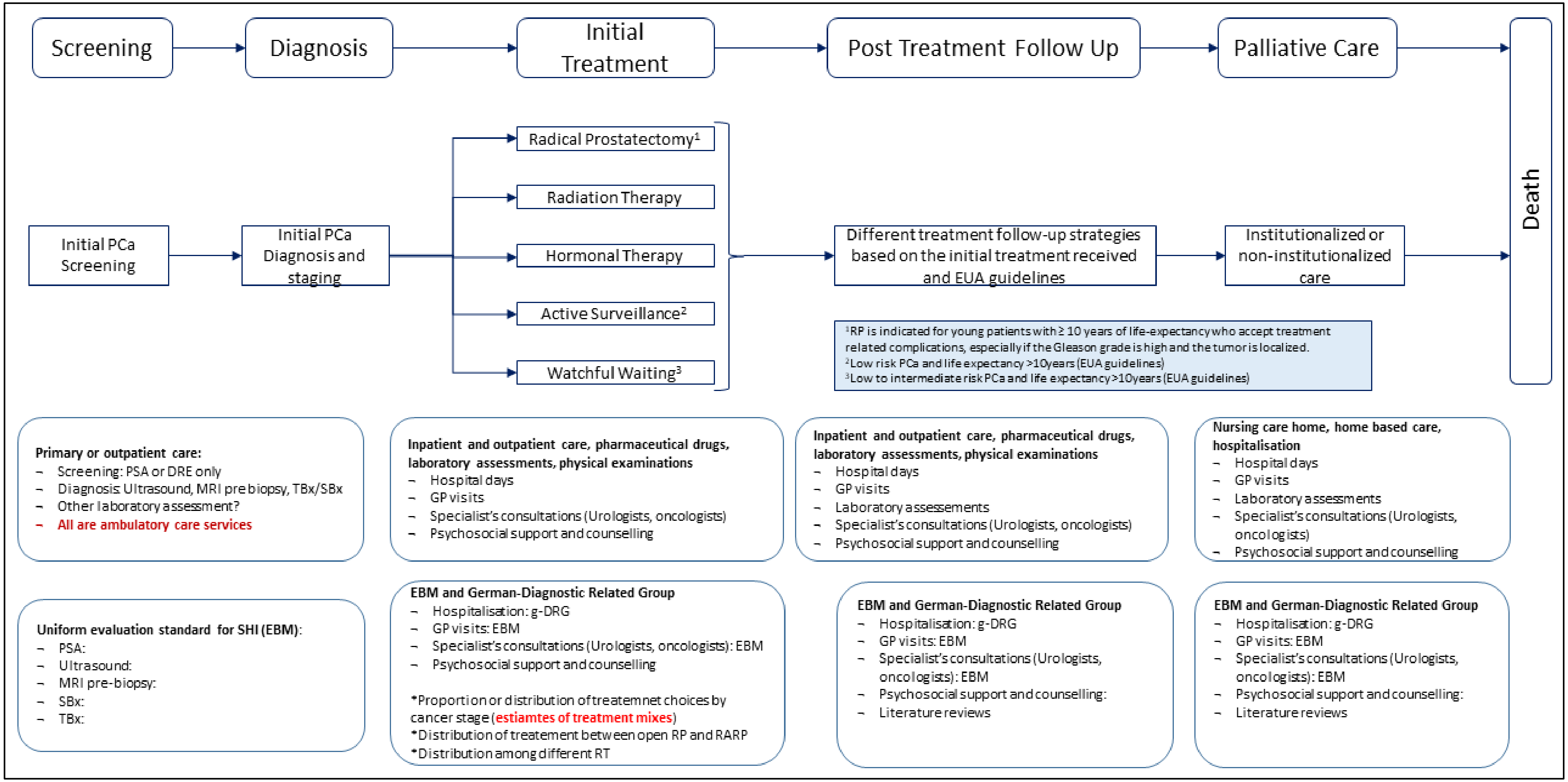

Figure S.5: Resource use along the prostate cancer care continuum

PCa: prostate cancer, EUA: European Urology Association, PSA: prostate specific antigen, DRE: digital rectal examination, GP: General Practitioner, SBx Standard transrectal ultrasound scan (TRUS) biopsy, MRI magnetic resonance imaging, g-DGR: Germany Diagnostic Related Group, EBM (Germany fee for service catalogue- the Einheitlicher Bewertungsmaßstab (EBM).

Source: Authors elaboration: Adapted from Hao et al 2020

5.1 Targeted literature review

To inform this estimation, we executed a rapid review using PubMed database, targeting prior cost-of-illness and cost-effectiveness analyses relevant to prostate cancer care in Germany. Using MeSH terms and keywords, we identified 12 key studies selected from 95 relevant to the Germany setting. Among these, five studies [5-9] provided valuable data informing the three-staged cost estimation process across the prostate cancer care continuum. Details of the 12 studies are provided in Table S.6

Additionally, German S3 and European Urology Association guidelines were reviewed for resource usage and quantities along the care continuum.

Table S.6: Costing and cost-effectiveness analysis studies specific for Germany setting.

Author and Year	Title	Objective(s)	Language (DE/ENG)	Considered informative (Yes/No)	Additional comments
**Stadlbauer et al 2011 [5]**	Health Economics Evaluation of Magnetic Resonance Imaging for the Staging of Prostate Cancer for Austria and Germany	To conduct a health economics analysis of MR imaging in the preoperative staging of patients with prostate carcinoma (prostate cancer).	DE	Yes	2011 costs of radical prostatectomy and radiation therapy, combined with hormone therapy and treatment side effects<br>Not much stated on how these were arrived at.
**Niklas et al 2016 [6]**	da Vinci and Open Radical Prostatectomy: Comparison of Clinical Outcomes and Analysis of Insurance Costs	To assess clinical outcomes and reimbursement costs of open and robotic-assisted radical prostatectomies in Germany	ENG	Yes	Total insurance benefit costs as paid by the SHI since 2009 to 2013, including sick pay and dental<br>T stage and Gleason score distribution for validation<br>OPS codes for open radical prostatectomy and robotic assisted radical prostatectomy
**Koerber et al 2014 [7]**	The cost-utility of open prostatectomy compared with active surveillance in early localised prostate cancer	To develop a decision-analytic model comparing the cost-utility of active surveillance (AS) and radical prostatectomy (PE) for a cohort of 65-year-old men with newly diagnosed low risk prostate cancer.	ENG	Yes	Cost of active surveillance, radical prostatectomy and radiation therapy based on g-DRG and EBM system.<br>Year specific DRG codes<br>Costing for metastatic prostate cancer

					A pivotal paper in the current study
**Fourcade et al 2010 [10]**	Treatment costs of prostate cancer in the first year after diagnosis: a short-term cost of illness study for France, Germany, Italy, Spain and the UK	To calculate the total cost per patient of prostate cancer treatment and the economic cost burden by stage, in the first year after diagnosis, for five European countries.	ENG	No	Mean direct costs per patient for initial PCA treatment radiation therapy radical prostatectomy, chemotherapy, hormone therapy, active surveillance or watchful waiting<br><br>Stage-wise percentage of initial prostate cancer treatments received<br><br>The cost distribution for all stages; initial treatment phase.
**Roehrborn et al 2011 [11]**	The economic burden of prostate cancer	To discuss expenditure on prostate cancer diagnosis, treatment and follow-up and evaluate the cost of prostate cancer and its management in different countries	ENG	No	Based on analysis by Fourcade et al 2010
**Reinhold et al 2016 [8]**	Treatment costs of localized prostate cancer in Germany: Economic results from the HAROW observational study	To determine the treatment costs associated with specific treatments of the locally limited prostate cancer for Germany. The patient-related follow-up costs for HT, AS, RT, RP and WW were examined.	DE	Yes	Annual cost along the prostate cancer continuum are presented from the societal, patient and SHI perspective based on observational data, EBM and g-DRG valuation<br><br>Could be useful as a comparative study with our current approach<br><br>g-DRGs codes relevant for billing were initially determined: M01A, M01B,

					M02A, M02B, M09A, M09B and M38Z<br>Could be a good estimate for Drug cost during follow up.
**Covers et al 2019 [12]**	Cost-effectiveness of SelectMDx for prostate cancer in four European countries: a comparative modelling study	To assess the cost-effectiveness of SelectMDx as a reflex test for biopsy decision within France, Germany, Italy, and Spain.	ENG	No	Treatment distribution of significant and insignificant prostate cancer<br>Costs associated with diagnosis, treatment and complications per patient<br>For Germany, most input parameters were informed by Koerber at al. 2014
**Schiffer et al 2012 [13]**	Urinary proteome analysis for prostate cancer diagnosis: Cost-effective application in routine clinical practice in Germany	To report treating physicians'' experience with routine application of Capillary electrophoresis mass spectrometry urinary proteome analysis for prostate cancer in clinical practice and its cost-effectiveness	ENG	No	Not much information on how the cost for biopsy and PSA were arrived at.<br>Could be useful for some other parameters.
**Herecomer et al 20 [14]**	Radical prostatectomy for men aged <56 years with prostate cancer	To analyse the cost of illness for patients <56 years after radical prostatectomy in the first 3 years after the operation	DE	No	
**Thomas Michaeli1 & Daniel Michaeli1 2020 [15]**	Prostate cancer follow-up costs in Germany from 2000 to 2015	To estimate and evaluate 10-year follow-up costs after prostate cancer treatment with curative (surgery, radiotherapy) and non-curative intent (hormone, androgen deprivation) per patient in Germany in 2000, 2008, and 2015.	ENG	No	10-year cumulative cost for curative treatment, hormone therapy with metastasis, hormone therapy without metastasis and ADT are reported form SHI, Patient and Societal perspective

					frequency of recommended consultations, examinations, and diagnostic tests was extracted from EAU guidelines<br><br>Reimbursement rates for private and statutory health insurance were extracted from the respective reimbursement catalogue: “Gebührenordnung für Ärzte (GOÄ)” and “Einheitlicher Bewertungsmaßstab (EBM)”
**Palmer et al 2019 [16]**	Literature review of the burden of prostate cancer in Germany, France, the United Kingdom and Canada	To characterize the clinical, economic and humanistic burden of disease associated with prostate cancer in France, Germany, the UK and Canada.	ENG	No	Only one study is listed from German regarding costs (Fourcade et al)
**Kreis et al 2020 [9]**	Treatment-Related Healthcare Costs of Metastatic Castration-Resistant Prostate Cancer in Germany: A Claims Data Study	To compare healthcare resource utilization (HRU) and costs in patients with metastatic castration resistant prostate cancer treated with cabazitaxel, docetaxel, abiraterone, enzalutamide, and best supportive care.	ENG	Yes	The analysis includes healthcare resource utilization and pharmaceutical costs (ready-to-use drugs and cytostatic agents), outpatient and inpatient care, and sick leave payments<br><br>Monthly cost for treatment on Cabazitaxel and docetaxel were estimated at €6343 and €1580 respectively

DE: Germany language, ENG: English language, ADT: androgen deprivation therapy, PSA: prostate specific antigen g-DGR: Germany Diagnostic Related Group, EBM (Germany fee for service catalogue- the Einheitlicher Bewertungsmaßstab (EBM)

5.2 Data extraction

One investigator (MTM) extracted relevant German Diagnosis-Related Groups (G-DRG) codes for inpatient services, the Einheitlicher Bewertungsmaßstab (EBM) codes for outpatient care and quantities of resources used. In some cases, where either the EBM or DRG code could not be established, cost values were extrapolated from previous studies and adjusted to 2023 rates using the consumer price index.

To assign unit prices for outpatient services, we used the 2023 physician's fee catalogue, whereas hospital-based resource usage was valued based on the federally agreed base case value (BBFW) for the year 2023, set at €4,000.71.

5.3 Expert opinion

To enhance the validity of our costing approach, an in-person interview was organised with an experienced urologist in Germany. One researcher (MTM) developed an interview guide (available upon request) for the consultations with the urologist based on the updated framework by Hao and colleagues and findings from the initial review. An in-person interview was then conducted by two researchers (MTM and MS) on 06.06.2023 to clarify our understanding of the EBM system pertaining to prostate cancer screening, diagnosis, staging, and post-treatment follow-up. Detailed summary based on the notes taken during the conversation were written by the one interviewer (MTM) [no audio recordings were made]. The notes and summary of the interview were sent to the expert for review and correction. A summary of the insights garnered from this interview which was used to refine our cost estimates is also available on request.

Table S.7 enumerates a comprehensive list of resource items, their corresponding values, and data sources.

Table S.7: Resources and unit costs characterization

Prostate cancer care pathways resource use and unit costs								
**Module/Procedure**	**Unit cost (€)**	**Resource use**		**Total item costs (€)**		**Source**	**EBM or DRG code**	**Additional notes**
**Screening component**								
**DRE plus PSA for positive DRE (Germany 2021-DRE + PSA)**								
Basic flat rate per insured person per quarter	21,26	0,2		4,25		EBM	26211<br>26212	Unweighted average of the basic fixed rate for each registered insured person, categorized by age groups: 6 to 59 years old (EBM 26211) and 60 years and above (EBM 26212), per quarter<br>This include as optional services that cannot be billed separately:<br>1) 01600 and 01601 per case of treatment: Medical report according to fee schedule item<br>2) 01436 in the same session: Consultation fee
Cancer screening test in men	16,55	0,5		8,28		EBM	01731	Mainly Colorectal and prostate cancer:<br>Need to apportion this by weighting with incidence of prostate cancer + CRC
PSA test	4,80	1		4,80		EBM	32351	Only for DRE positive:
PSA laboratory test analysis	20,81	1		20,81		Assumption<br>price based on private lab online price: https://city-labor.de/profile-125.html		Only for DRE positive: No need for adjustment. Private sector charges 4.99€ for Blood sampling which cost 4.80 to SHI. Therefore, adjustment = 20.81/4.99*4.80 =20.01€
Advice on early cancer detection in men	13,33	0,5		6,67			01740	One-time consultation based on information provided to insured persons about the aim and purpose of the program for the early detection of cancer
**Total costs**		**19,19**		**44,81**				
**PSA only**								
Invitation to participate	0,86	1		0,86		EBM	40110	Flat-rate fee for sending or transporting a letter and/or written documents

Prostate cancer care pathways resource use and unit costs								
**Module/Procedure**	**Unit cost (€)**	**Resource use**		**Total item costs (€)**		**Source**	**EBM or DRG code**	**Additional notes**
Basic flat rate per insured person per quarter	21,26	0,2		4,25		EBM	26211<br>26212	Unweighted average of the basic fixed rate for each registered insured person, categorized by age groups: 6 to 59 years old (EBM 26211) and 60 years and above (EBM 26212), per quarter<br>This include as optional services that cannot be billed separately:<br>1) 01600 and 01601 per case of treatment: Medical report according to fee schedule item<br>2) 01436 in the same session: Consultation fee
Cancer screening test in men	16,55	0,5		8,28		EBM	01731	Mainly colorectal and prostate cancer:<br>Need to apportion this by weighting with incidence of prostate cancer + CRC
PSA test	4,80	1		4,80		EBM	32351	As a primary test
PSA laboratory test analysis	20,81	1		20,81				As a primary test
Advice on early cancer detection in men	13,33	0,5		6,67			01740	One-time consultation based on information provided to insured persons about the aim and purpose of the program for the early detection of cancer
**Total costs**				**45,67**				
MRI								
MRI	121,01	1		121,01		EBM	34442	
Standard biopsy: Transrectal ultrasound (TRUS) guided punch biopsy at outpatient care:								
Basic flat rate per insured person per quarter	21,26	1		21,26		EBM	26211<br>26212	Unweighted average of the basic fixed rate for each registered insured person, categorized by age groups: 6 to 59 years old (EBM 26211) and 60 years and above (EBM 26212), per quarter<br>This include as optional services that cannot be billed separately:<br>1) 01600 and 01601 per case of treatment: Medical report according

Prostate cancer care pathways resource use and unit costs								
**Module/Procedure**	**Unit cost (€)**	**Resource use**		**Total item costs (€)**		**Source**	**EBM or DRG code**	**Additional notes**
								to fee schedule item<br>2) 01436 in the same session: Consultation fee
Urogenital sonography	9,42	1		9,42		EBM	33042	
Surcharge for transcavitary examination	6,55	1		6,55			33090	
Standard prostate biopsy	19,65	1		19,65		EBM	26341	This includes optional services such as additional punctures (Note only 6 cores are reimbursed) and local anaesthesia that cannot be billed separately
Pathology	9,54	1		9,54		EBM	19310	Reported value and in agreement with what used before in Koerber et al 2014<br>Will use ±50% and a high value of €500 in the DSA to align with regional prices
**Total costs**				**66,42**				
prostate cancer staging								
Bone scintigraphy	65,04	1		65,04		EBM	17311	
CT Scan	66,77	1		66,77		EBM	34342	
**Total costs**				**131,81**				
**Active surveillance: standard biopsy**		**Per year (<2yrs)**	**Per year (>2yrs)**	**Per year (<2yrs)**	**Per year (>2yrs)**			
1. PSA and DRE monitoring								

Prostate cancer care pathways resource use and unit costs								
**Module/Procedure**	**Unit cost (€)**	**Resource use**		**Total item costs (€)**		**Source**	**EBM or DRG code**	**Additional notes**
Basic flat rate per insured person per quarter	21,26	0,8	0,4	17,01	8,50	EBM	26211 26212	Unweighted average of the basic fixed rate for each registered insured person, categorized by age groups: 6 to 59 years old (EBM 26211) and 60 years and above (EBM 26212), per quarter<br>This include as optional services that cannot be billed separately:<br>1) 01600 and 01601 per case of treatment: Medical report according to fee schedule item<br>2) 01436 in the same session: Consultation fee
Additional flat rate oncology	21,95	4	2	87,80	43,90	EBM	26315	Additional flat-rate treatment and/or care of a patient with a confirmed oncological disease during ongoing oncological therapy or care as part of aftercare<br>In the event of treatment, the fee schedule item 26315 cannot be billed in addition to the flat-rate fees 86510, **86512**, 86514, 86516 and 86520 in accordance with Annex 2 of the "Oncology Agreement" (Annex 7 to the Federal Shell Contract for Physicians (BMV-Ä))
PSA test sampling	4,8	4	2	19,20	9,60	Updated Koerber et al. 2014 using 2023 EBM values	32351	
PSA laboratory test analysis	20,81	4	2	83,25	41,63	Assumption		
2. Prostate re-biopsies during AS								
Urogenital sonography	9,42	1	0,33	9,42	3,11	EBM	33042	
Surcharge for transcavitary examination	6,55	1	0,33	6,55	2,16		33090	
Standard prostate biopsy	19,65	1	0,33	19,65	6,48	EBM	26341	This includes optional services such as additional punctures (Note only 6 cores are reimbursed) and local anaesthesia that cannot be billed separately
Pathology	9,54	1	0,33	9,54	3,15	EBM	19310	
**Total cost *(Annual cost)***		1		**252,42**	**118,53**			Assume average prostate cancer diagnosis is 67 years in Germany and the average life expectancy is 80Yeras

Prostate cancer care pathways resource use and unit costs								
**Module/Procedure**	**Unit cost (€)**	**Resource use**		**Total item costs (€)**		**Source**	**EBM or DRG code**	**Additional notes**
**Active surveillance MRI and combined biopsies**		**per year (<2yrs)**	**Per year (>2yrs)**	**per year (<2yrs)**	**Per year (>2yrs)**			
1. PSA and DRE monitoring								
Basic flat rate per insured person per quarter	21,26	0,8	0,4	17,01	8,50	EBM	26211<br>26212	Unweighted average of the basic fixed rate for each registered insured person, categorized by age groups: 6 to 59 years old (EBM 26211) and 60 years and above (EBM 26212), per quarter<br>This include as optional services that cannot be billed separately:<br>1) 01600 and 01601 per case of treatment: Medical report according to fee schedule item<br>2) 01436 in the same session: Consultation fee
Additional flat rate oncology	21,95	4	2	87,80	43,90	EBM	26315	Additional flat-rate treatment and/or care of a patient with a confirmed oncological disease during ongoing oncological therapy or care as part of aftercare<br>In the event of treatment, the fee schedule item 26315 cannot be billed in addition to the flat-rate fees 86510, **86512**, 86514, 86516 and 86520 in accordance with Annex 2 of the "Oncology Agreement" (Annex 7 to the Federal Shell Contract for Physicians (BMV-Ä))
PSA test sampling	4,8	4	2	19,20	9,60	Updated Koerber et al. 2014 using 2023 EBM values	32351	
PSA laboratory test analysis	20,81	4	2	83,25	41,63	Assumption		
2. Prostate re-biopsies during AS								
MRI	121,01	1	0,333	121,01	40,34	Updated Koerber et al. 2014 [7] using 2023 EBM values	34442	This was used in the sensitivity analysis with a base cost of 500 euros based of information available online in Germany.

Prostate cancer care pathways resource use and unit costs								
**Module/Procedure**	**Unit cost (€)**	**Resource use**		**Total item costs (€)**		**Source**	**EBM or DRG code**	**Additional notes**
Combined prostate biopsy	71,24	1	0,333	71,24	23,75	EBM	26341	This includes optional services such as additional punctures (Note only 6 cores are reimbursed) and local anaesthesia that cannot be billed separately
Pathology (sample assessment)	9,54	1	0,333	9,54	3,18	Assumption (Units based on Koerber et al 2014)		
**Total cost *(Annual cost)***				**409,05**	**170,89**			
**Radical prostatectomy (RP)**		**Per Year**		**Per Year**				
Specialist and nurse consultation	119,63	1		119,63		Updated Koerber et al. 2014 using 2023 EBM values	25211	
RP surgery without complication	9.349,66	0,69		6.404,52		g-DRG base rate and complication rates from Riedler et al. 2020	M01B	In previous study, specifically Koeber et al. (2014) [7], two Diagnosis-Related Group (DRG) codes are utilized for open radical prostatectomy: M01A, which is used for cases with complications, and M01B, for those without complications. It is also noted that there is no distinct DRG for robot-assisted prostatectomy. Complication rates are documented in Rödler's dissertation and are also available in the S3 guidelines
RP surgery with complication	13.978,48	0,32		4.403,22		g-DRG base rate and complication rates from Riedler et al. 2021	M01A	
**Total cost**		**1**		**10.927,37**				
**Radiation therapy**		**Per Year**						
Oncologist consultation - new visit	119,63	1		119,63		Updated Koerber et al. 2014 using 2023 EBM values	25211	
CT Planning	40,68	1		40,68		Updated Koerber et al. 2014 using 2023 EBM values	34360	
Radiation plans	545,16	2		1.090,32		Updated Koerber et al. 2014 using 2023 EBM values	25342	

Prostate cancer care pathways resource use and unit costs								
**Module/Procedure**	**Unit cost (€)**	**Resource use**		**Total item costs (€)**		**Source**	**EBM or DRG code**	**Additional notes**
Lump sum/radiation field	164,13	15		2.461,91		Updated Koerber et al. 2014 using CPI	40840	
Radiation	110,32	40		4.412,80		Updated Koerber et al. 2014 using 2023 EBM values	25321	
> 2 fields	7,60	40		303,87		Updated Koerber et al. 2014 using CPI		
3D planning	11,09	40		443,61		Updated Koerber et al. 2014 using CPI		
**Total cost**				**8.872,83**				
Metastatic prostate cancer treatment								
Annual medical treatment costs (ADT/Chemo/HT)	26.293,40	1		26.293,40		Updated Kreis et al 2021		See section 5.1.3 of the ESM for more detail
Annual treatment cost -Bone metastasis	3.806,23	0,7		2.664,36		Updated Koerber et al. 2014		See section 5.1.3 of the ESM for more detail
**Total cost *(Annual cost)***				**28.957,76**				
**Post treatment follow-up: first year**		**per year (<2yrs)**	**Per year (>2yrs)**	**per year (<2yrs)**	**Per year (>2yrs)**			Given MRI patient=TRUE: This means post Treatment follow up uses MRI
Post radical prostatectomy and radiation therapy								
Basic flat rate per insured person per quarter	21,26	0,8		17,01		EBM	26211<br>26212	Unweighted average of the basic fixed rate for each registered insured person, categorized by age groups: 6 to 59 years old (EBM 26211) and 60 years and above (EBM 26212), per quarter<br>This include as optional services that cannot be billed separately:<br>1) 01600 and 01601 per case of treatment: Medical report according to fee schedule item<br>2) 01436 in the same session: Consultation fee

Prostate cancer care pathways resource use and unit costs								
**Module/Procedure**	**Unit cost (€)**	**Resource use**		**Total item costs (€)**		**Source**	**EBM or DRG code**	**Additional notes**
Additional flat rate oncology	21,95	4	2	87,80	43,90	EBM	26315	Additional flat-rate treatment and/or care of a patient with a confirmed oncological disease during ongoing oncological therapy or care as part of aftercare<br>In the event of treatment, the fee schedule item 26315 cannot be billed in addition to the flat-rate fees 86510, **86512**, 86514, 86516 and 86520 in accordance with Annex 2 of the "Oncology Agreement" (Annex 7 to the Federal Shell Contract for Physicians (BMV-Ä))
PSA test sampling	4,80	4	2	19,20	9,60		32351	
PSA test analysis	20,81	4	2	83,25	41,63			
2. Prostate re-biopsies during post treatment follow up (combined biopsy)								
MRI	121,01	1	0,333	121,01	40,34	Updated Koerber et al. 2014 using 2023 EBM values	34442	This was used in the sensitivity analysis with a base cost of 500 euros based of information available online in Germany.
Combined prostate biopsy	71,24	1	0,333	71,24	23,75			
Pathology	9,54	1	0,33	9,54	3,15	EBM	19310	
**Total cost *(Annual cost)***				**294,70**	**115,31**			
**Palliative therapy**								
Yearly cost/patient	40.185,68	1	1	40.185,68	40.185,68	Updated Rusche et al., 2016 using CPI		
**Total cost *(Annual cost)***				**40.185,68**	**40.185,68**			
**Terminal illness**	40.185,68	0,5	0,5	20.092,84	20.092,84	Updated Rusche et al., 2016 using CPI		Assumed end of life is for 6 months
Yearly drug cost/patient				**20.092,84**	**20.092,84**			

Prostate cancer care pathways resource use and unit costs								
**Module/Procedure**	**Unit cost (€)**	**Resource use**		**Total item costs (€)**		**Source**	**EBM or DRG code**	**Additional notes**
GP: General Practitioner; MRI: Magnetic Resonance Imaging; PSA: Prostate-specific Antigen; RT: Radiation Therapy: All prices are at 2023 prices								

5.4 Special consideration to the costing approach

5.4.1 Consultation or assessment cost

The fee for service catalogue in German (Einheitlicher Bewertungsmaßstab [EBM]) serves as a framework for billing outpatient services, determining the content and value using a point-based system. Billing schedules are separately constructed for individual services, bundled services, fixed (flat) rates for insured persons, basic, additional flat rates and other categories such as structural flat rates and quality-based surcharges. Given its multi-faceted nature, the EBM is a complex system with significant variations in its application across different service providers.

It is worth noting that consultations with urologists and general practitioners are billed on a per-case and per-quarter basis. This means that a patient insured under the same health insurance fund can only be billed once within a calendar quarter by the same medical practitioner or practice. For the purpose of our study, we considered the following elements in the calculation of doctors' consultation fees:

- The unweighted average of the basic fixed rate for each registered insured person, categorized by age groups: 6 to 59 years old (EBM 26211) and 60 years and above (EBM 26212), per quarter.
- An additional fixed rate applied specifically to the management of cancer patients once prostate cancer (prostate cancer) is confirmed (EBM 26315), which is predominantly relevant to post-diagnostic follow-up visits.

5.4.2 Metastatic prostate cancer treatment cost

Our base case analysis cost of €28,957.76 for androgen deprivation therapy and or chemotherapy therapy (ADT/Chemo) was based on the comprehensive analysis by Kreis et al. [9] adjusted for 2023 values. This figure encompasses a range of therapies for patients receiving ADT/Chemotherapy, including chemotherapeutic agents (Docetaxel + Prednisolone) and androgen receptor targeted agents [ARTA] (Enzalutamide, Abirateron). Additionally, we incorporated the cost of zoledronic acid to address the expected bone metastasis in 70% of prostate cancer patients, drawing from prior cost-effectiveness research (ref). This methodological choice ensures our cost estimates cover a broad spectrum of treatment scenarios, even though it excludes the more recent and costlier treatment options, such as Apalutamide and Darolutamide, which were not available during the timeframe of the studies we referenced

Metastatic prostate cancer (mPCa) is an advanced stage where the cancer spreads to distant body parts like bones and lungs. This stage significantly impacts both the cost and quality of life, with many patients diagnosed at this stage or rapidly progressing to it [17].

The primary treatment for mPCa has been castration—the reduction of testosterone levels, typically achieved either through surgical removal of the testes (surgical castration—orchiectomy) or through medication (androgen deprivation therapy—ADT), to manage the progression of the disease by depriving cancer cells of the androgens required for growth [18]. Nevertheless, most patients become non-responsive (resistant) to castration, typically with two to three years of treatment initiation [17]. As a result, two types of mPCa are described in literature: 1)

metastatic disease sensitive (responsive) to ADT, known as metastatic castration-sensitive prostate cancers (mCSPCa), and 2) metastatic castration resistant prostate cancer (mCRPCa) [7, 9, 17, 19].

ADT remains a key treatment, but recent years have seen the introduction of expensive chemotherapeutic agents such as Docetaxel and Cabazitaxel, and innovative of Androgen-Receptor-Targeted Agents (ARTA) such as Enzalutamide, Abirateron, Apalutamide and Darolutamide) [7, 9, 17, 19, 20]. Notably, a significant proportion of patients also rely on best supportive care (BSC) for symptom management. In Germany, approximately 60% of mCRPCa patients were on BSC from 2014 to 2017 [9].

Treatment decisions for mPCa are influenced by various guidelines and factors, including symptoms, therapy side effects, patient preferences, comorbidities, life expectancy, and tumour burden. While, the chemotherapeutic agents such as Docetaxel and Cabazitaxel, and ARTA agents—Abiraterone, Enzalutamide, Apalutamide and Darolutamide have been approved by the US Food and Drug Administration (FDA), the European Medicines Agency (EMA), the National Comprehensive Cancer Network (NCCN) and the European Society Medical Oncology (ESMO) for mCSPCa treatment, uptake at local levels depends on physician and patient's preference as well as local guidelines. This complexity and patient heterogeneity mean that no model can fully capture these decision-making factors.

Therefore, our cost estimation for mPCa is primarily informed by a real-world analysis of prostate cancer costs in Germany [9]. The study elucidated adjusted and unadjusted monthly cost for different for chemotherapies (docetaxel and cabazitaxel), hormonal drugs abiraterone and enzalutamide, and best supportive care (BSC) using 2014–2017 claims data from a large German statutory health insurance fund, the Techniker Krankenkasse [9]

To calculate annual mPCa treatment costs, we first calculated the expected mean monthly cost for the 4 treatment options based on the proportional representation of the patients receiving a specific treatment. We then compounded the monthly mean to a yearly average. Since the cost in this study were not adjusted to a single year currency, and include cost from 2014 to 2017 we used the last reporting year as the base year and inflated the yearly average to 2023 based values using the World Bank CPI inflation calculator. These computations are shown in Table S.8. However, these costs focus on mCRPCa.

Koerber and colleagues reported that 70% of mPCa patients develop bone metastases, treated primarily with radiation therapy and bisphosphonates. In their calculation they used radiation therapy and bisphosphates, in particular Zoledronic acid as the primary treatment of bone metastasis. We included these costs in our total annual treatment estimation. As a conservative approach, we will add 0.7 * treatment cost of bone metastases to arrive at the total annual treatment cost of mPCa per patient. Table S.9 highlights the estimation of the total treatment cost for bone metastatic cancer.

Table S.8: Conservative estimated treatment cost for metastatic prostate cancer in Germany

Drug/Treatment path	Size	Expectation	Unadjusted cost*	Adjusted cost**	Expected cost 1†	Expected cost 2‡
**Cabazitaxel**	240	0,06	7.631	6.343	464,36	385,98
**Docetaxel**	539	0,14	2.392	1.580	326,90	215,93
**Abiraterone**	486	0,12	5.226	4.579	643,97	564,25
**Enzalutamide**	351	0,09	5.079	4.416	452,01	393,01
**BSC**	2.328	0,59	959	438	566,06	258,54
Aggregation/monthly average cost	**3.944**	**1,00**			**2.453,31**	**1.817,70**
**Number of months in a year**					12	12
Yearly average cost (2017 base value)					29.439,69	21.812,42
**Yearly average cost (2023 base value)**					**35.487,56**	**26.293,40**

Table S.9: Estimation of treatment cost for bone metastasis

Module/Procedure	Unit cost (€)	Resource use per year	Total costs (€)	Source	EBM or DRG code
**Radiation therapy**					
Oncologist consultation - new visit	119,63	1	119,63	Updated Koerber et al. 2014 using 2023 EBM values	25211
CT Planning	40,68	1	40,68	Updated Koerber et al. 2014 using 2023 EBM values	34360
Radiation plans	545,16	2	1.090,32	Updated Koerber et al. 2014 using 2023 EBM values	25342
Radiation	110,32	14	1.544,48	Updated Koerber et al. 2014 using 2023 EBM values	25321
**Medication**					
Zoledronic acid	84,26	12	1.011,12	Germany drug catalogue	PZN = 09929186
**Sub-total cost**			**3.806,23**		
Factor in probability of occurrence			**0,70**	Koerber et al. 20140	
**Expected annual drug cost for bone metastasis**			**2.664,36**		

The G-BA benefit assessment document for Apalutamide for use in patients with mCSPCa (Module 3A)[1] provides a range of annual therapy costs for patients receiving ADT, in combination combinations with Docetaxel + Prednisolone **(€12,697 per year),** Abiraterone + Prednisolone **(€49,187.21 per patient per year**) or Apalutamide **(€54,000 per patient per year)**, suggesting a broad cost spectrum based on treatment regimens. Similarly, a Swiss budget impact analysis reflects this cost range, with price per patient per year ranging between €6,953 for ADT alone to €36,229 for ADT in combination with Apalutamide [21].

Given this variability, adopting an average cost of **€28,957.76,** informed by the best form of real-world evidence available for ADT and chemotherapy appears reasonable within the German context. This estimate considers multiple factors affecting treatment decisions, underscoring the need for a detailed medical insurance data analysis to inform accurate utilisation rates—which did not materialise in our case and can be considered as future research to update model input parameters

We acknowledge the limitation in our cost estimation for this disease stage. First, there is a potential for double counting some cost that has already been included in the study by Kreis et al [9]. Second, the inflation calculator used in the cost inflation is not the best proxy for price changes with time. Changes in pharmaceuticals prizes since introduction on to the market is derived not just by inflation, but by other factors such as patents and availability of generics, expansion of therapeutic indication (market size), which usually reduce (instead of escalate) the prices with time. For instance, Wang and colleagues [17] noted that docetaxel and abiraterone acetate has become generically available in the United States, while enzalutamide and Apalutamide remain under patent protection. A similar occurrence is also expected in Europe since the patent for abiraterone acetate expired in 2022.

To mitigate potential underestimations in our cost analysis, we conducted a deterministic sensitivity analyses, increasing and reducing ADT costs by 50% and 100% (See section 8.1.9), and subsequently a 95% CI of ± 20% in the probabilistic sensitivity analysis.

5.4.3 Palliative and terminal care cost

Palliative care is multi-faceted, specialized healthcare approach aimed at enhancing the well-being of patients with ***progressive or advanced disease who have limited life expectancy***. It also extends support to their families [2, 22]. The core objective of palliative care is to alleviate symptoms, pain, and stress associated with severe illnesses to allow patients to die with dignity and minimal discomfort. Typically, patients receiving palliative care have exhausted curative treatments or have become unresponsive to available treatments, leaving them without prospects of cure [2, 22].

While the terms "palliative care," "end-of-life care," and "terminal care" are often used interchangeably, they differ in key areas such as timing, scope, and objectives. All these approaches aim to improve the quality of life for patients with serious or terminal illnesses, but they differ in timing, scope, and objectives. Palliative care may begin at any stage of illness and can coexist with curative treatments [23]. In contrast, end-of-life and terminal

[1] https://www.g-ba.de/downloads/92-975-3559/2020-02-24_Modul3A_Apalutamid.pdf (Accessed 22 Feb 2024

care specifically focus on the final stages of life, generally when life expectancy is ***less than six months***, and solely ***emphasize patient comfort and quality of life***.

In Germany and globally, the organization of palliative and terminal care varies based on settings and service types and often categorized as follows [2, 22, 24, 25]:

1. Specific palliative or hospice inpatient units
2. Hospital-based advisory services (inpatient or outpatient)
3. Community-based services (home [usual place of residence], inpatient hospice-based, inpatient and outpatient and advisory services)

In Germany, specialized outpatient palliative care was introduced in 2007 to complement general outpatient palliative care. Specialized care primarily targets severe cases and is usually provided at home by palliative physicians to a predominantly oncology patients. Approximately 55% of the total costs for both types of outpatient palliative care stem from inpatient care costs [2, 26].

A key assumption underlying our cost analysis is that palliative and terminal care needs are generally independent of disease type [2]. While cancer is a common diagnosis leading to outpatient palliative care, the needs of these patients are considered similar to those with non-oncologic conditions [2]. Our study extrapolates this assumption, asserting that the costs and resource use for palliative care are dictated not by disease type, but by disease stage and the form of palliative care received.

Our targeted review found no specific data on resource and cost use for palliative care among prostate cancer patients in Germany. Furthermore, there is considerable variation in reported average palliative care costs relevant to the German setting. For instance, one study estimated the mean cost of general and specialized palliative care in the last year and three months of life to be €24,981.71 and €31,743.60 respectively [26]. Another study reported yearly total cost of €2737 for various forms of palliative care combined [24]. These studies, albeit analysing similar data sources[2], diverged significantly in the cost components they considered. Specifically, the study by Rusche and colleagues [26] included inpatient palliative care in hospitals which were not included by the other study [24].

Given this backdrop, our cost estimates for palliative and terminal care were informed by the more comprehensive study which estimated the combined mean cost of general and specialized palliative care at €31,743.60 for the year 2012 [26]. After adjusting for inflation using an online CPI calculator specific to Germany, this figure equates to **€40,185.68** for the year 2023[3]

Finally, we assumed that the cost of terminal care in the last six months of life would be half the annual cost of palliative care.

---

[2] Both studies retrospectively analysed real word (routine) data from insured persons provided by two major medical insurance providers in German (Krankenkasse (DAK) and BARMER)
[3] https://www.officialdata.org/germany/inflation/2012?amount=31743.60 [Accessed 01.09.2023]

6 Cost-effectiveness analysis

The aggregated costs and health outcomes, including prostate cancer deaths, life-years, and QALYs for the seven screening strategies, discounted to their net present values were compared with a no-screening baseline.

Following established guidelines [27], incremental cost-effectiveness ratios (ICERs) were computed exclusively for efficient strategies. Efficient strategies were identified by excluding those "strongly dominated" or "extendedly dominated" from the list of competing options. A strategy is classified as strongly dominated if it is less effective and costlier than an alternative or extendedly dominated if the additional health benefits are achieved at a prohibitively higher marginal cost—when a strategy have a higher ICERs than a more effective strategy.

The ICER for the remaining efficient strategies was calculated as follows:

$$ICER = \frac{Cost_A - Cost_B}{QALYs_A - QALYs_B} \quad (1)$$

In this equation, A and B represent the sets of strategies that remain after the dominated options are removed.

Cost effectiveness planes were used to represent the results of base case analyses. These planes delineate the effectiveness of compared interventions across four quadrants as shown in Figure S.6

Strategies in the SE quadrant are straightforward decisions since they are dominant (more effective and less costly), while those in the NW quadrant are typically rejected because they are dominated (costlier and less effective).

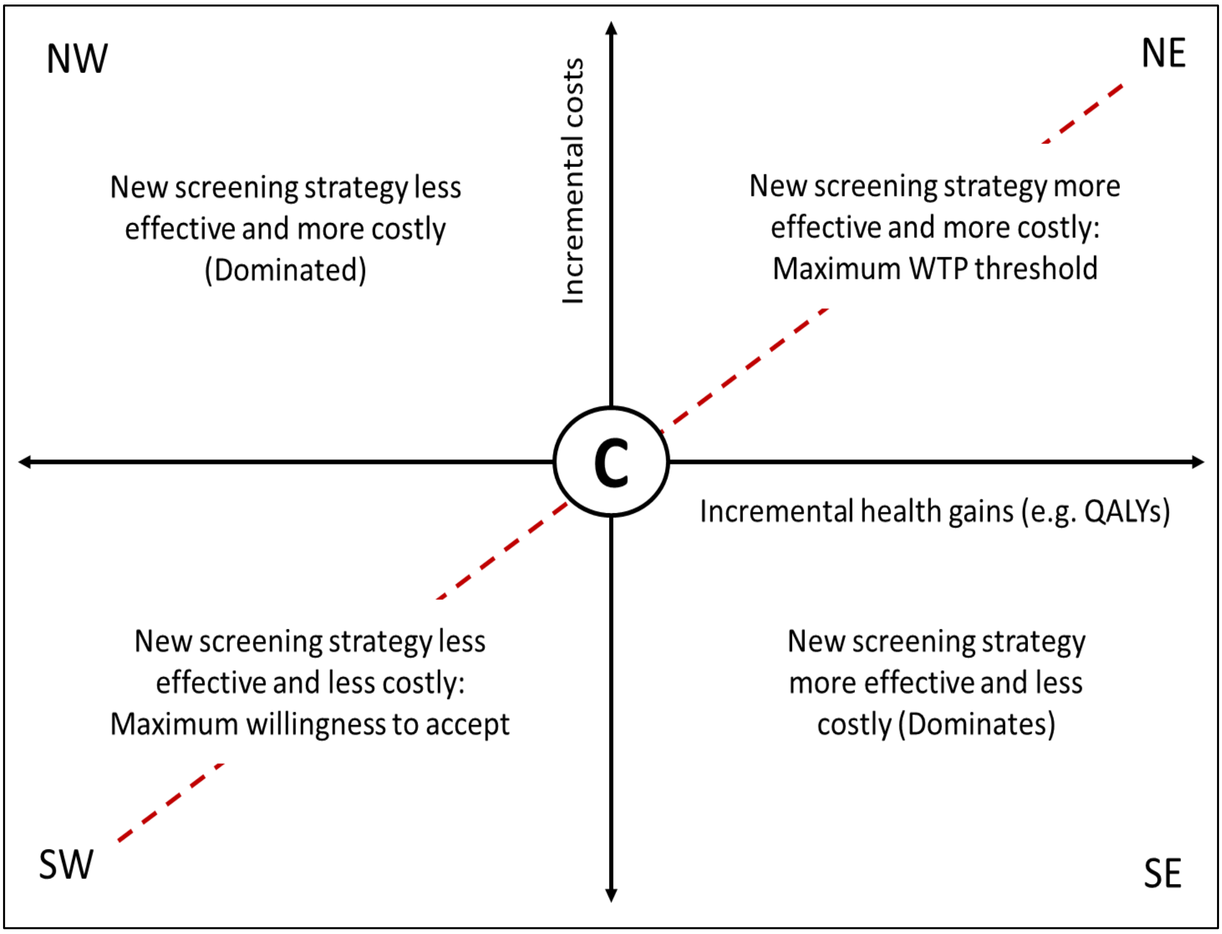

Figure S.6: The cost-effectiveness plane and the decision rules
C: Baseline Comparator — "No Screening"
Each strategy is compared to No Screening, positioned at the origin (0,0). Note that No Screening entails costs (see Section 5.3 for assumptions). The red dotted line indicates the hypothetical willingness-to-pay (hWTP) threshold, which guides interpretation of cost-effectiveness ratios. An optimal strategy maximizes health gains (extends along the x-axis) while minimizing incremental costs (minimal increase along the y-axis).

Decision rules are required to help decide on those strategies in the NE quadrant that offers additional health gains (effective) at increased costs and those in the SW quadrant that have less health gains for less cost. As a result, the relevance of an ICER is contingent on a benchmark indicating decision makers' willingness to pay (WTP) per incremental unit of projected health improvement [61]. Some countries such as UK have a clearly defined WTP threshold of 30,000 per QALY gained. As Germany lacks standardised WTP thresholds, we adopted arbitrary (hypothetical) WTP values of 20,000; 50,000 and 100,000 euros per QALY gained.

Crucially, in any cost-effectiveness analysis, a strategy does not necessarily need to be cost-saving to be considered cost-effective. More often, new interventions result in increased costs and effectiveness (NE quadrant), necessitating various decision rules or frameworks to determine if the incremental costs are justified by the incremental health gains.

Supplementary results

7 Sensitivity analysis

7.1 Deterministic sensitivity analysis

7.1.1 Impact of varying the cost of mPCa care

The landscape of metastatic prostate cancer care in Europe and worldwide is rapidly evolving, driven by the introduction of innovative therapies and their subsequent approval by the European Medical Agency. For metastatic castration-resistant prostate cancer (mCRPC), the use of second-generation antihormonal agents, such as Apalutamide, Enzalutamide, and Abiraterone, alongside androgen deprivation therapy (ADT), represents a notable leap forward in care, significantly enhancing life expectancy.

Similarly, the approval of these therapies in combination with androgen deprivation therapy (ADT) for metastatic hormone-sensitive prostate cancer (mCSPCa) signifies a significant shift in treatment approaches. Notably, the management of metastatic prostate cancer is increasingly moving towards personalised approaches, which consider patient-specific factors and preferences in a context marked by a lack of direct comparisons between existing therapies.

However, there is a notable gap in the availability of cost information for these treatments to inform health economic evaluations. Rapid advancements in therapy have surpassed the current scope of economic assessments, highlighting a gap in our understanding. Our cost analysis for metastatic prostate cancer (mPCa) relies on real-world data from 2014 to 2017, provided by Techniker Krankenkasse, a major German health insurance fund. This analysis detailed the costs associated with chemotherapies such as docetaxel and cabazitaxel, hormonal therapies such as abiraterone and enzalutamide, and best supportive care (BSC). However, recent approvals of drugs, such as Apalutamide, have not been adequately represented.

To mitigate potential underestimations in our cost analysis, we conducted sensitivity analyses, increasing and reducing ADT costs by 50% and 100% from the baseline cost of **€28,957.76.**

This cost increase causes a downward vertical shift in all screening strategies compared to no screening, indicating reduced incremental costs (Figure S.7). Notably, non-MRI-based strategies, such as PSA risk-adaptive screening starting at ages 45-60 without MRI, become cost-saving relative to no screening. Similarly, PSA risk-adaptive screening without MRI starting at ages 50-60 shows even greater cost savings under these conditions.

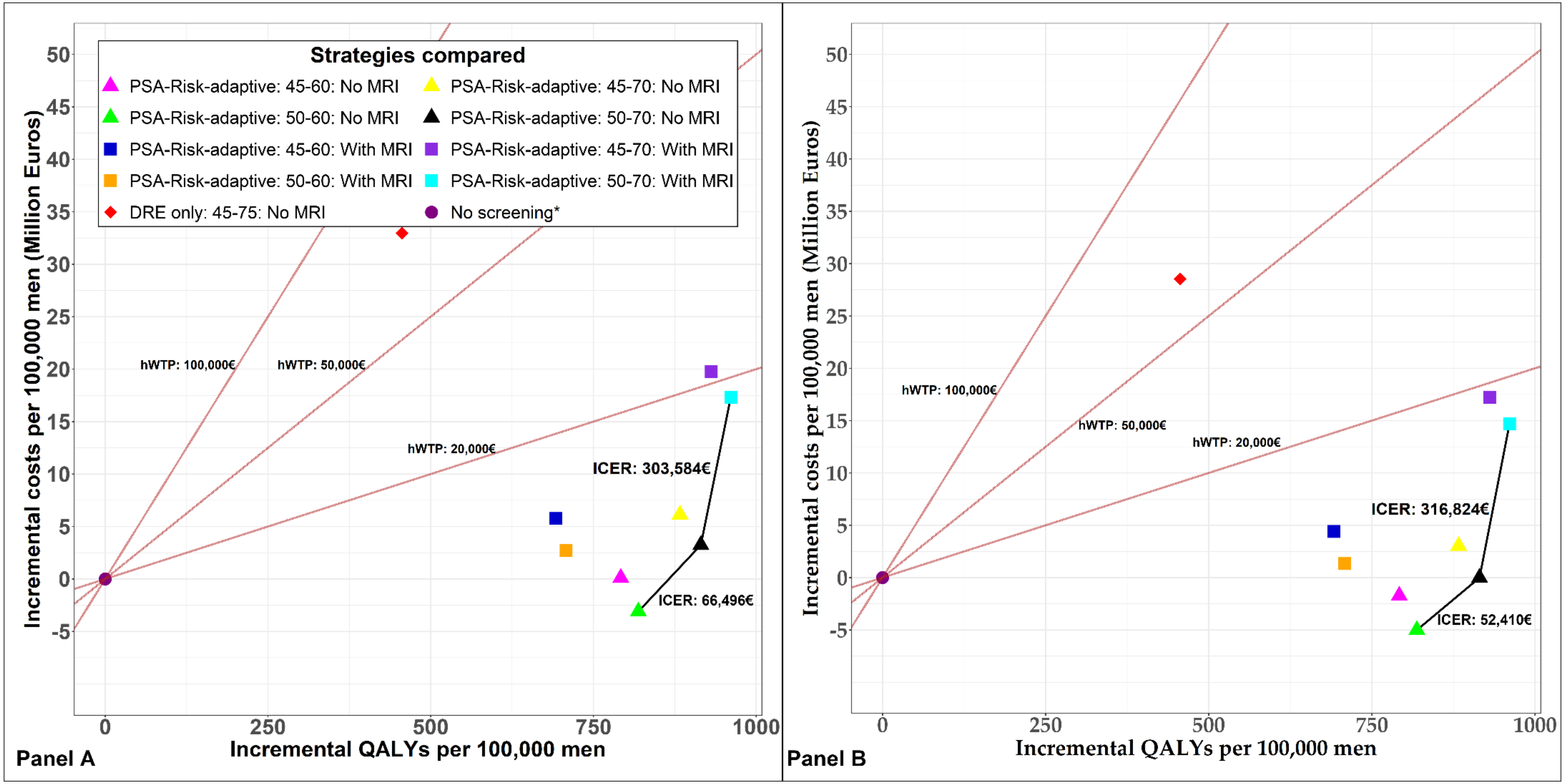

Figure S.7: DSA: Impact of uncertainty on cost of ADT/chemotherapy
Panel A: Impact of increasing ADT/chemotherapy cost by 50% **Panel B:** Impact of increasing metastatic prostate cancer care cost by 100%
DRE, digital rectal examination; with MRI means MRI is used to inform biopsy decisions followed by combined systemic (SBx) and targeted (TBx) biopsies. Strategies without MRI, "No MRI", do not include MRI use to triage biopsy decision, suspicious cases are followed by systemic biopsies. *In the base case analysis, all symptomatically detected cancers were assumed to be further investigated using MRI imaging followed by combined biopsies, the red dotted lines represent the various hypothetical willingness to pay threshold (hWTP) used as the decision rule to interpret the cost-effectiveness ratio
Source: Author's elaboration

7.1.2 Impact of health states utility values

The sensitivity of cost-effectiveness analyses to utility values is well-documented. Research by Sanghera and colleagues [28] and studies by Hao and colleagues [33-35] demonstrated the considerable impact of different utility value sets on model outcomes. Specifically, Hao et al. found significant sensitivity in their model to EQ-5D derived values, contrasting with the stability seen with Heijnsdijk et al.'s [36] utility values.

In line with Hao et al.'s [35] methodology, our analysis integrates their comprehensive systematic review and meta-analysis of health state utility values along the prostate cancer continuum, in which they extended Magnus et al.'s [37] work to include data up to 2020. This review yielded two key sets of utility values: generic EQ-5D and disease-specific Patient-Oriented Prostate Utility Scale-Utility (PORPUS-U) values. Despite EQ-5D's broad acceptance by many standard practice guidelines and HTA bodies, we contend that it falls short in capturing the nuanced utility changes associated with prostate cancer progression, such as those related to sexual dysfunction or urinary incontinence, areas where PORPUS-U demonstrates superior specificity. Therefore, our base case analysis employed the PORPUS-U disease-specific utility values reported by Hao et al, acknowledging a trade-off in comparability with other diseases.

Figure S.8 illustrates the outcomes using EQ-5D utilities—and we caution against its straightforward interpretation. While EQ5D utilities appear to yield higher QALY gains for MRI-based strategies when screening is limited to 60 years, a general QALY loss is observed across all strategies compared to base case results using PORPUS-U utilities (refer to Figure 2 in the main text). Notably, there's a correlation between QALY loss per strategy and the number of biopsy events per screening. For instance, "DRE only" followed by "PSA-RAS without MRI" stopping screening at 70, then "PSA-RAS with MRI" stopping at 70, exhibited the largest QALY losses in that order. Hao et al.'s [35] findings, though involving different strategies, align with ours, particularly noting the discrepancies in utilities for radical prostatectomy and metastatic states between PORPUS-U and EQ5D. Table S.10 further elucidates the impacts of this extension on clinical and cost outcomes.

This result is unsurprising—the EQ5D reported utilities are notably lower than those from PORPUS-U, especially for radical prostatectomy—part 1 (0.90 for PORPUS-U vs 0.82), metastatic disease state (0.803 vs 0.727), and post-recovery period (0.93 vs 0.861), underscoring the need for caution in utility selection (see Table 1: Model input parameters, for further comparison).

Furthermore, while the uncertainty in these values can be mitigated to a significant extent through probabilistic sensitivity analysis, this highlights the importance of carefully considering utility value selection in CEA. Given these significant differences and the changing prostate cancer treatment landscape—highlighted by innovations such as robotic-assisted surgeries and MRI diagnostics— transforming what was once daunting into possibly positive encounters—we urge further research into health state utility values across the prostate cancer care continuum.

We advocate for a dual research approach: first, a multi-country empirical elicitation of health state utility values across the prostate cancer continuum to capture diverse patient experiences and clinical practices; second, the development of quality appraisal tools for assessing these studies, addressing a current gap in the literature. Such

efforts will ensure utility values accurately reflect the benefits of contemporary clinical practices and patient experiences.

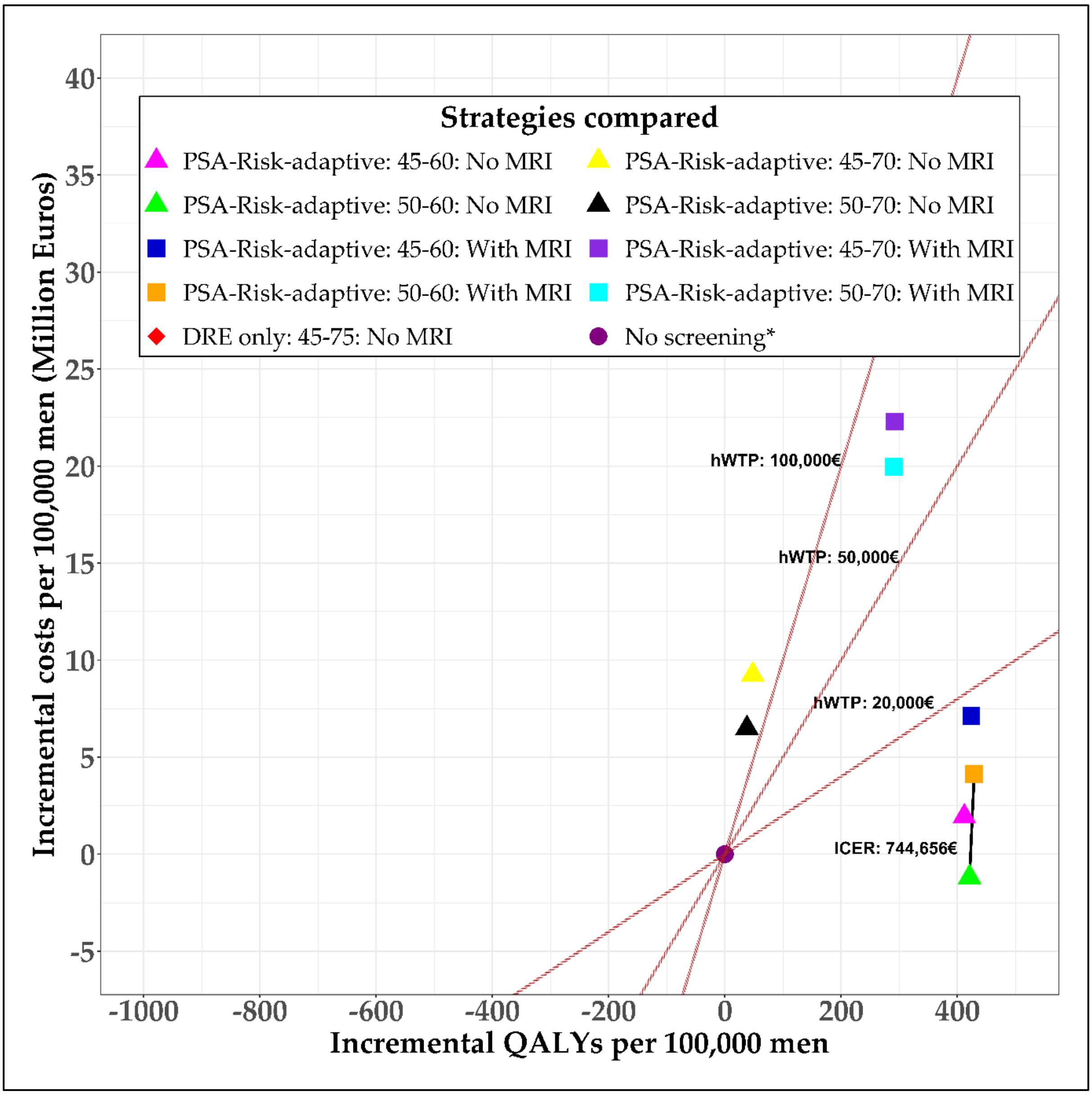

Figure S.8: DSA: Impact of using EQ5D health states utility values.
Due to significant overdiagnosis and overtreatment, the is a substantial loss in QALYs for DRE only.
DRE, digital rectal examination; with MRI means MRI is used to inform biopsy decisions followed by combined systemic (SBx) and targeted (TBx) biopsies. Strategies without MRI, "No MRI", do not include MRI use to triage biopsy decision, suspicious cases are followed by systemic biopsies*In the base case analysis, all symptomatically detected cancers were assumed to be further investigated using MRI imaging followed by combined biopsies, the red dotted lines represent the various hypothetical willingness to pay threshold (hWTP) used as the decision rule to interpret the cost-effectiveness ratios.
Source: Author's elaboration

Table S.10: Model outcomes by strategy from age 45: Use of EQ-5D values

	PSA-Risk-adaptive: 50-60: No MRI	No screening*	PSA-Risk-adaptive: 45-60: No MRI	PSA-Risk-adaptive: 50-60: With MRI	PSA-Risk-adaptive: 50-70: No MRI	PSA-Risk-adaptive: 45-60: With MRI	PSA-Risk-adaptive: 45-70: No MRI	PSA-Risk-adaptive: 50-70: With MRI	PSA-Risk-adaptive: 45-70: With MRI	DRE only: 45-75: No MRI
Outcomes per 100,000 men										
Number of DRE test	0	0	0	0	0	0	0	0	0	1876319
Number of PSA tests	219869	0	284427	221082	367051	285572	424222	371180	428132	0
Number of MRI events	0	0	0	13486	0	13000	0	51798	49367	0
Total number biopsy	29235	19672	28965	20714	62169	20701	60230	26069	25792	161126
Number of clinically-initiated biopsies	16508	19672	16683	17312	12985	17437	13334	14073	14365	10001
Number of screen-initiated biopsies	12727	0	12282	3402	49184	3264	46897	11995	11427	151125
Number of diagnosed PCa	10788	10474	10771	10675	11714	10665	11654	11377	11330	12942
Number of clinically diagnosed PCa	8805	10474	8897	9223	6930	9289	7115	7492	7647	5329
Number of screen diagnosed PCa	1983	0	1874	1452	4784	1376	4539	3885	3683	7613
Number of Localised and G < 7	3840	3628	3828	3732	4414	3727	4378	4098	4072	5115
Number of Localised and G = 7	3509	3416	3504	3505	3778	3500	3759	3766	3748	4123
Number of Localised and G > 7	3439	3430	3439	3438	3522	3438	3518	3513	3510	3704
Number of metastatic PCa diagnosed	1731	2018	1745	1795	1373	1805	1405	1448	1476	1036
Estimated overdiagnosed PCa	314	0	297	202	1240	191	1180	903	857	2468
Prostate cancer deaths	3321	3657	3336	3409	3010	3417	3041	3117	3142	2718
LY, undiscounted	3462414	3458500	3462290	3461555	3464970	3461489	3464724	3464031	3463836	3467354
LY, discounted at 3%	2092631	2091200	2092592	2092335	2093452	2092317	2093375	2093138	2093078	2094220
QALYs, undiscounted	3117193	3114637	3117115	3116725	3117469	3116684	3117378	3117417	3117337	3116737
QALYs, discounted at 3%	1911125	1910704	1911116	1911133	1910742	1911128	1910752	1910995	1910996	1909765
QALYs, discounted at 5%	1473468	1473528	1473470	1473566	1473074	1473567	1473095	1473340	1473351	1472195
Costs (M€) per 100,000 men										
Healthcare perspective, undiscounted	405	426	409	417	408	420	411	436	438	443
Healthcare perspective, discounted at 3%	165	166	168	170	172	173	175	186	188	203
Healthcare perspective, discounted at 5%	97	94	100	101	104	104	107	113	115	131
ICERs (€ per QALY): Compared with No screening	Dominant	**Reference case**	4701	9656	170222	16796	193956	68725	76370	Dominated
ICERs on Efficient Frontier (€ per QALY)	**First on Frontier**	Dominated	Dominated	744656	Dominated	Dominated	Dominated	Dominated	Dominated	Dominated

Columns are arranged in order of increasing healthcare perspective cost discounted at 3%. PSA: prostate specific antigen; MRI: magnetic resonance imaging; DRE: digital rectal examination; PCa: prostate cancer; LY: life years; GS: Gleason score; ICER: incremental cost-effectiveness ratio; QALY. Quality adjusted life years

7.1.3 Scenario 2 and 3: Different PSA biopsy threshold and rescreening intervals:

Sanghera and colleagues [28] suggested that screening men at very high risk (PSA ≥ 4 ng/ml) every four years could enhance the cost-effectiveness of PSA-based screening. However, opinions vary regarding the optimal age to start and stop screening, with Heijnsdijk and colleagues [29] recommending 55-59/60, while other studies propose cessation between ages 70 to 71 [30-32].

Scenario 2 tested exempting low-risk men (PSA < 2 ng/ml) from re-screening, offering a single screening round to reduce interventions and overdiagnosis while maintaining clinical benefits. Moderate-risk (PSA 2–4 ng/ml) and high-risk (PSA ≥ 4 ng/ml) groups were re-screened every five years starting at age 55 until the age of 70. This approach reduced biopsies by 52% but increased overdiagnosis by 18%. MRI-based strategies reduced biopsies by 135% and overdiagnosis by 2%. DRE-only strategies reduced biopsies by 45% and overdiagnosis by 53% compared to starting DRE at 45 and stopping at 75. Cost savings were significant, with PSA screening (55–70), without MRI becoming cost-saving, achieving €4.3 million per 100,000 men over a lifetime.

Scenario 3 evaluated the impact of halting further screenings for individuals in low PSA risk groups (<1.5 ng/ml), while instituting screenings every five years for those in moderate (PSA > 1.5 ng/ml and < 3 ng/ml) and high PSA risk groups (PSA ≥ 3 ng/ml), recommending biopsies at a PSA level of 3 ng/ml. Re-screening concludes at age 70 for all participants, including those with negative MRI and biopsy results who are also screened every five years until this age. ***The Digital Rectal Examination (DRE) screening stopping age remained at 75 years***.

MRI-based strategies reduced MRI events by up to 94% and biopsies by 92%. Overdiagnosis decreased by up to 93%, but all strategies saw increases in PCa-specific deaths, life years lost, and QALYs lost. Despite these drawbacks, PSA screening (45/50–70) without MRI became cost-saving due to reduced resource use and overdiagnosis.

Figure S.9 Panels A and B, and Tables S.11 and S.12 details the clinical and cost effects of this scenario. Notably the QALY gains in both scenarios are less than those obtained in the base case scenarios

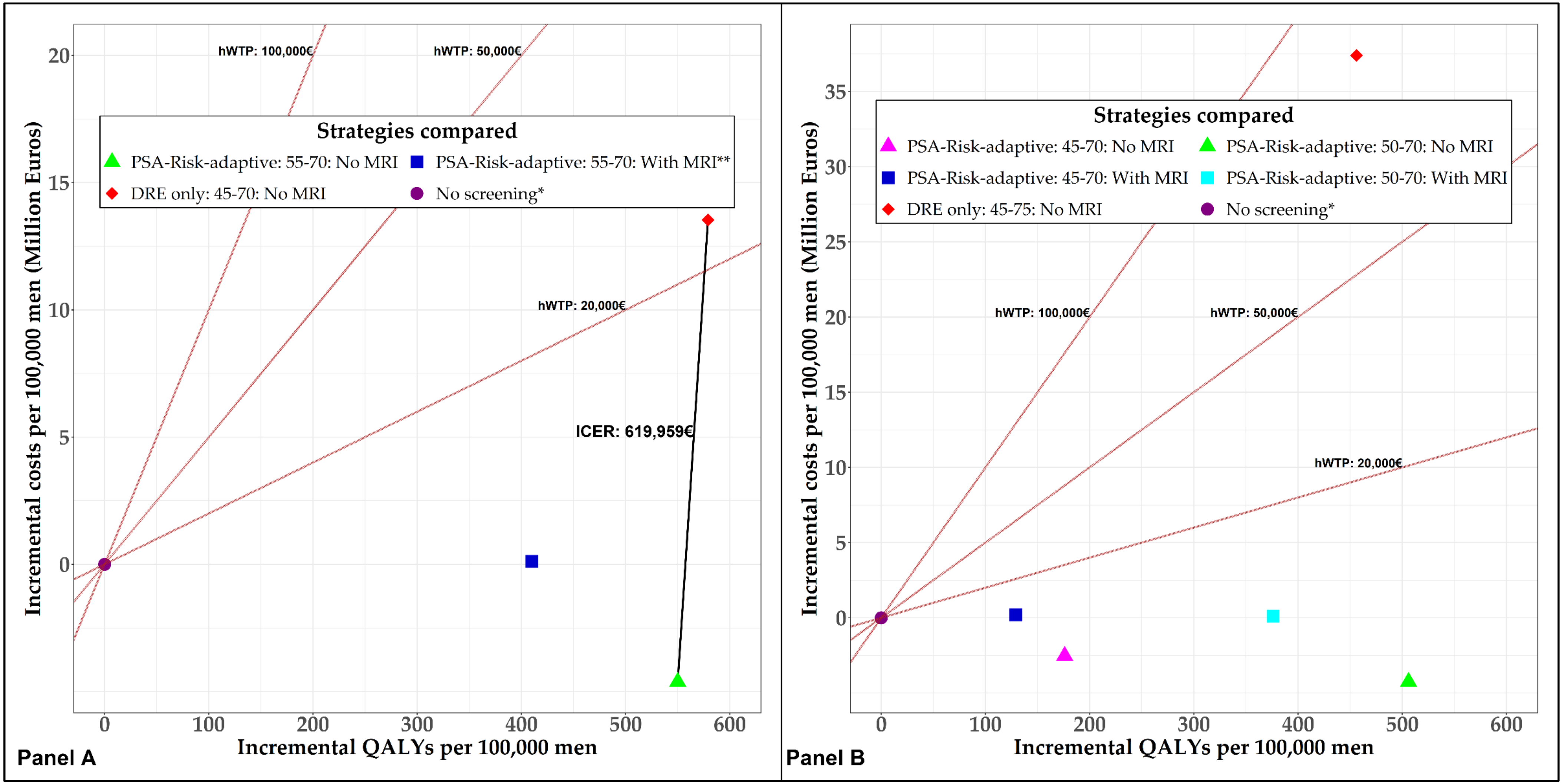

Figure S.9: DSA: Panel A: Scenario 2 and Panel B: Scenario 3

Note: Each panel displays fewer than ten strategies because, as the starting and stopping ages are adjusted, certain strategies converge into unified approaches. PSA, prostate specific antigen; DSA: deterministic sensitivity analysis; QALYs, quality-adjusted life-years; DRE, digital rectal examination; with MRI means MRI is used to inform biopsy decisions followed by combined SBx and TBx. Strategies without MRI, "No MRI", do not include MRI use to triage biopsy decision, suspicious cases are followed by systemic biopsies. *In the base case analysis, all symptomatically detected cancers were assumed to be further investigated using MRI imaging followed by combined SBx and TBx, the red dotted lines represent the various hypothetical willingness to pay threshold (hWTP) used as the decision rule to interpret the cost-effectiveness ratios.

Source: Author's elaborations

Table S.11: Model outcomes by strategy from age 45: Scenario 2

	**PSA-Risk-adaptive: 55-70: No MRI**	**No screening***	**PSA-Risk-adaptive: 55-70: With MRI**	**DRE only: 55-75: No MRI**
**Outcomes per 100,000 men**				
Number of DRE test	0	0	0	952720
Number of PSA tests	84881	0	85449	0
Number of MRI events	0	0	8504	0
Total number biopsy	24278	19672	19869	93968
Number of clinically initiated biopsies	16138	19672	17387	11592
Number of screen-initiated biopsies	8140	0	2482	82376
Number of diagnosed PCa	10856	10474	10689	12226
Number of clinically diagnosed PCa	8613	10474	9263	6198
Number of screen diagnosed PCa	2243	0	1426	6029
Number of Localised and G < 7	3895	3628	3736	4770
Number of Localised and G = 7	3520	3416	3514	3906
Number of Localised and G > 7	3440	3430	3439	3550
Number of metastatic PCa diagnosed	1727	2018	1816	1252
Estimated overdiagnosed PCa	382	0	215	1752
Prostate cancer deaths	3375	3657	3477	2924
LY, undiscounted	3461495	3458500	3460504	3465449
LY, discounted at 3%	2092257	2091200	2091920	2093562
QALYs, undiscounted	3121266	3119139	3120602	3122893
QALYs, discounted at 3%	1913137	1912587	1912997	1913166
QALYs, discounted at 5%	1474825	1474640	1474798	1474595
**Costs (M€) per 100,000 men**				
Healthcare perspective, undiscounted	403	426	416	416
Healthcare perspective, discounted at 3%	161	166	166	179
Healthcare perspective, discounted at 5%	93	94	96	110
ICERs (€ per QALY): Compared with No screening	Dominant	Reference case	283	23347
ICERs on Efficient Frontier (€ per QALY)	**First on the frontier**	Dominated	Dominated	619959

Columns are arranged in order of increasing healthcare perspective cost discounted at 3%. PSA: prostate specific antigen; MRI: magnetic resonance imaging; DRE: digital rectal examination; PCa: prostate cancer; LY: life years; GS: Gleason score; ICER: incremental cost-effectiveness ratio; QALY. Quality adjusted life years

Table S.12 Model outcomes by strategy from age 45: Scenario 3

	PSA-Risk-adaptive: 50-70: No MRI	PSA-Risk-adaptive: 45-70: No MRI	No screening*	PSA-Risk-adaptive: 50-70: With MRI	PSA-Risk-adaptive: 45-70: With MRI	DRE only: 45-75: No MRI
**Outcomes per 100,000 men**						
Number of DRE test	0	0	0	0	0	1876319
Number of PSA tests	83521	76300	0	84010	76400	0
Number of MRI events	0	0	0	5922	818	0
Total number biopsy	23022	19938	19672	19840	19610	161126
Number of clinically-initiated biopsies	17413	19187	19672	18183	19335	10001
Number of screen-initiated biopsies	5609	751	0	1656	275	151125
Number of diagnosed PCa	10662	10494	10474	10582	10486	12942
Number of clinically diagnosed PCa	9286	10216	10474	9686	10294	5329
Number of screen diagnosed PCa	1376	277	0	896	192	7613
Number of Localised and G < 7	3763	3641	3628	3686	3634	5115
Number of Localised and G = 7	3466	3422	3416	3463	3422	4123
Number of Localised and G > 7	3434	3430	3430	3433	3430	3704
Number of metastatic PCa diagnosed	1839	1979	2018	1893	1989	1036
Estimated overdiagnosed PCa	188	20	0	108	12	2468
Prostate cancer deaths	3464	3615	3657	3531	3628	2718
LY, undiscounted	3460812	3459150	3458500	3460086	3458969	3467354
LY, discounted at 3%	2092052	2091460	2091200	2091795	2091390	2094220
QALYs, undiscounted	3120905	3119667	3119139	3120373	3119518	3123430
QALYs, discounted at 3%	1913093	1912763	1912587	1912963	1912716	1913043
QALYs, discounted at 5%	1474832	1474718	1474640	1474797	1474698	1474339
**Costs (M€) per 100,000 men**						
Healthcare perspective, undiscounted	407	415	426	417	420	443
Healthcare perspective, discounted at 3%	162	163	166	166	166	203
Healthcare perspective, discounted at 5%	93	94	94	96	95	131
ICERs (€ per QALY): Compared with No screening	Dominant	Dominant	Reference case	307	1597	81987
ICERs on Efficient Frontier (€ per QALY)	Dominant	Dominant	Dominated	Dominated	Dominated	Dominated

Columns are arranged in order of increasing healthcare perspective cost discounted at 3%. PSA: prostate specific antigen; MRI: magnetic resonance imaging; DRE: digital rectal examination; PCa: prostate cancer; LY: life years; GS: Gleason score; ICER: incremental cost-effectiveness ratio; QALY. Quality adjusted life years

7.1.4 Effects of varying stopping ages for DRE and PSA risk adaptive screening

Exploring an alternative hypothesis, we considered whether lowering the cessation age for DRE screening could improve its cost-effectiveness. The rationale is that lowering the age for cessation of DRE screenings to 70 could reduce the absolute number of tests (DRE examinations and screen-initiated biopsies) and, consequently, the rates of overdiagnosis and overtreatment in older individuals are unlikely to benefit from such interventions. To explore this idea, we adjusted the cessation age for DRE screening to 70 years while maintaining the stopping age at 60 years for PSA risk-adaptive strategies, which are grounded in trialled strategies.

Table S.13 compares the outcomes of DRE screenings halting at age 75 (as per the base case) and 70 (as explored in the sensitivity analysis). The anticipated benefits were realised with a significant decrease in screening-initiated biopsies (16%) and overdiagnosis (-30%). However, these advantages were observed at a cost: a 6% increase in prostate cancer-specific mortality and a 22% increase in metastatic cancer cases at diagnosis. The analysis revealed negligible changes in health-related quality of life and a modest 3% reduction in healthcare costs.

Table S.13: Selected outcomes comparing DRE only stopping at 75 or 70

Metric description	DRE screening up to 75	DRE screening up to 70	Absolute change	% Change
Screen initiated biopsies	151,125	127,644	23,481	-16%
Number of prostate cancer cases diagnosed	12,942	12,191	751	-6%
Number of metastatic cancer cases diagnosed	1,036	1,261	-225	22%
Estimated overdiagnosed prostate cancer	2,468	1,717	751	-30%
Prostate cancer deaths	2,718	2,881	-163	6%
QALYs, discounted at 3%	1,913,043	1,913,073	-30	0%
Healthcare perspective, discounted at 3%	2,032	1,979	53	-3%

Figure S.10 presents the corresponding ICERs on the cost-effectiveness plane following this age adjustment. Despite the reduced stopping age, the DRE-only strategy remains less economically favourable (dominated), incurring higher costs for fewer QALYs per 100,000 men than the other evaluated strategies.

Further details of the clinical and cost outcomes of these two variations are provided in Table S.14.

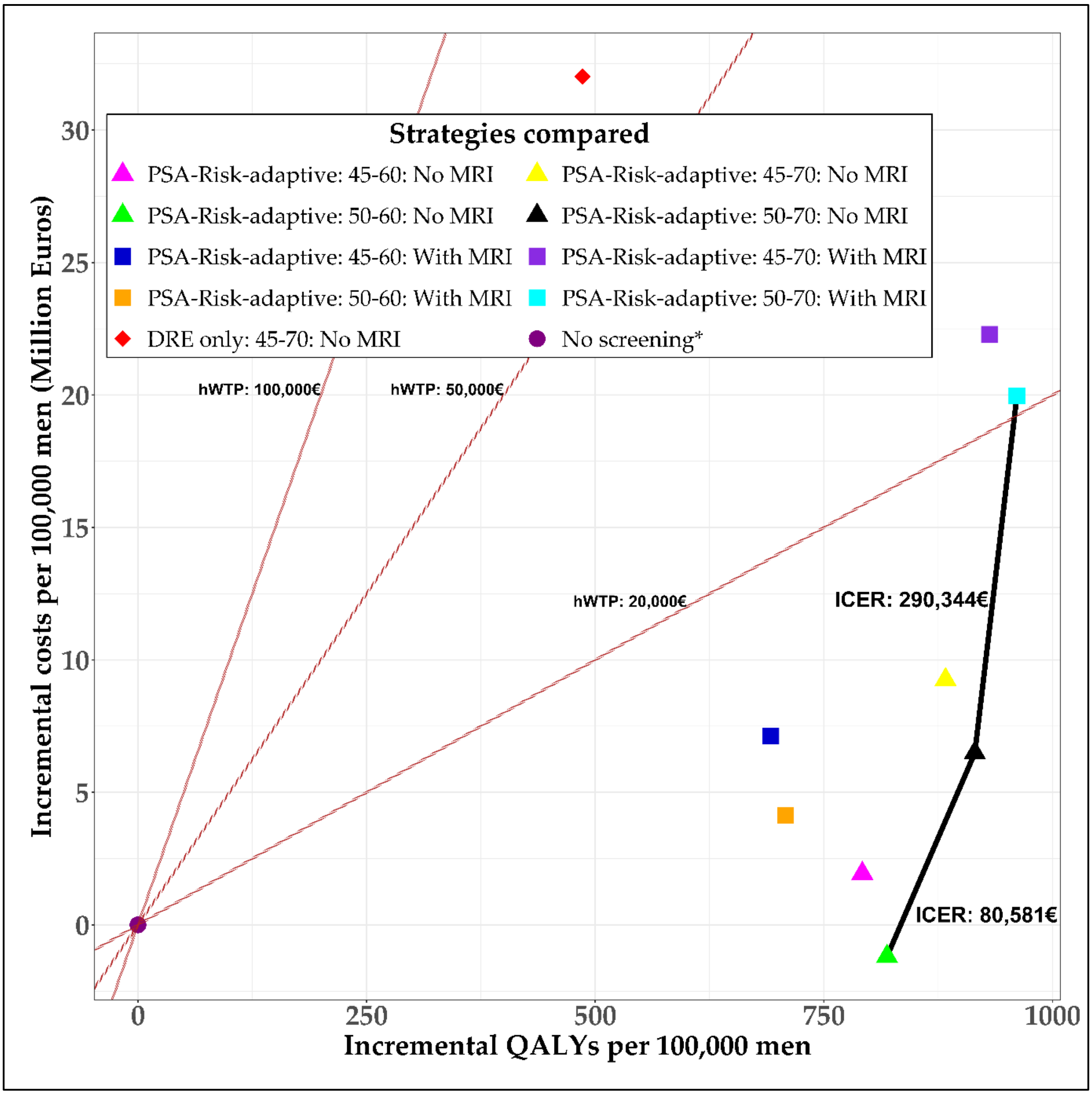

Figure S.10: DSA: Impact of Lowering Stopping Age in DRE-Only Strategy (**75 to 70**)
DRE, digital rectal examination; with MRI means MRI is used to inform biopsy decisions followed by combined SBx and TBx. Strategies without MRI, "No MRI", do not include MRI use to triage biopsy decision, suspicious cases are followed by systemic biopsies. *In the base case analysis, all symptomatically detected cancers were assumed to be further investigated using MRI imaging followed by combined SBx and TBx, the red dotted lines represent the various hypothetical willingness to pay threshold (hWTP) used as the decision rule to interpret the cost-effectiveness ratios
Source: Author's elaboration

Table S.14: Model outcomes by strategy from age 45: Reduce DRE stopping age to 70 years

	PSA-Risk-adaptive: 50-60: No MRI	No screening*	PSA-Risk-adaptive: 45-60: No MRI	PSA-Risk-adaptive: 50-60: With MRI	PSA-Risk-adaptive: 50-70: No MRI	PSA-Risk-adaptive: 45-60: With MRI	PSA-Risk-adaptive: 45-70: No MRI	PSA-Risk-adaptive: 50-70: With MRI	PSA-Risk-adaptive: 45-70: With MRI	DRE only: 45-70: No MRI
**Outcomes per 100,000 men**										
Number of DRE test	0	0	0	0	0	0	0	0	0	1647703
Number of PSA tests	219869	0	284427	221082	367051	285572	424222	371180	428132	0
Number of MRI events	0	0	0	13486	0	13000	0	51798	49367	0
Total number biopsy	29235	19672	28965	20714	62169	20701	60230	26069	25792	139286
Number of clinically-initiated biopsies	16508	19672	16683	17312	12985	17437	13334	14073	14365	11642
Number of screen-initiated biopsies	12727	0	12282	3402	49184	3264	46897	11995	11427	127644
Number of diagnosed PCa	10788	10474	10771	10675	11714	10665	11654	11377	11330	12191
Number of clinically diagnosed PCa	8805	10474	8897	9223	6930	9289	7115	7492	7647	6228
Number of screen diagnosed PCa	1983	0	1874	1452	4784	1376	4539	3885	3683	5963
Number of Localised and G < 7	3840	3628	3828	3732	4414	3727	4378	4098	4072	4746
Number of Localised and G = 7	3509	3416	3504	3505	3778	3500	3759	3766	3748	3899
Number of Localised and G > 7	3439	3430	3439	3438	3522	3438	3518	3513	3510	3546
Number of metastatic PCa diagnosed	1731	2018	1745	1795	1373	1805	1405	1448	1476	1261
Estimated overdiagnosed PCa	314	0	297	202	1240	191	1180	903	857	1717
Prostate cancer deaths	3321	3657	3336	3409	3010	3417	3041	3117	3142	2881
LY, undiscounted	3462414	3458500	3462290	3461555	3464970	3461489	3464724	3464031	3463836	3466191
LY, discounted at 3%	2092631	2091200	2092592	2092335	2093452	2092317	2093375	2093138	2093078	2093872
QALYs, undiscounted	3122116	3119139	3122016	3121523	3123221	3121468	3123069	3122885	3122757	3123213
QALYs, discounted at 3%	1913406	1912587	1913379	1913295	1913502	1913279	1913470	1913548	1913518	1913073
QALYs, discounted at 5%	1474928	1474640	1474915	1474922	1474859	1474914	1474850	1474964	1474952	1474389
**Costs (M€) per 100,000 men**										
Healthcare perspective, undiscounted	405	426	409	417	408	420	411	436	438	435
Healthcare perspective, discounted at 3%	165	166	168	170	172	173	175	186	188	198
Healthcare perspective, discounted at 5%	97	94	100	101	104	104	107	113	115	128
ICERs (€ per QALY): Compared with No screening	Dominant	**Reference case**	2444	5843	7095	10296	10472	20762	23940	65799
ICERs on Efficient Frontier (€ per QALY)	**First on Frontier**	Dominated	Dominated	Dominated	80581	Dominated	Dominated	290344	Dominated	Dominated

Columns are arranged in order of increasing healthcare perspective cost discounted at 3%. PSA: prostate specific antigen; MRI: magnetic resonance imaging; DRE: digital rectal examination; PCa: prostate cancer; LY: life years; GS: Gleason score; ICER: incremental cost-effectiveness ratio; QALY. Quality adjusted life years

7.1.5 Discounting cost and health at 5% instead of 3%

The discounting of costs and health outcomes remains a contentious issue in health economics, with ongoing debate about appropriate methods, rates, and assumptions. In this study, we evaluated the impact of applying a higher discount rate to both costs and health outcomes on the reported ICERs. Additionally, we examined the effect of not applying discounting. The results are presented on a cost-effectiveness plane in Figure S.11

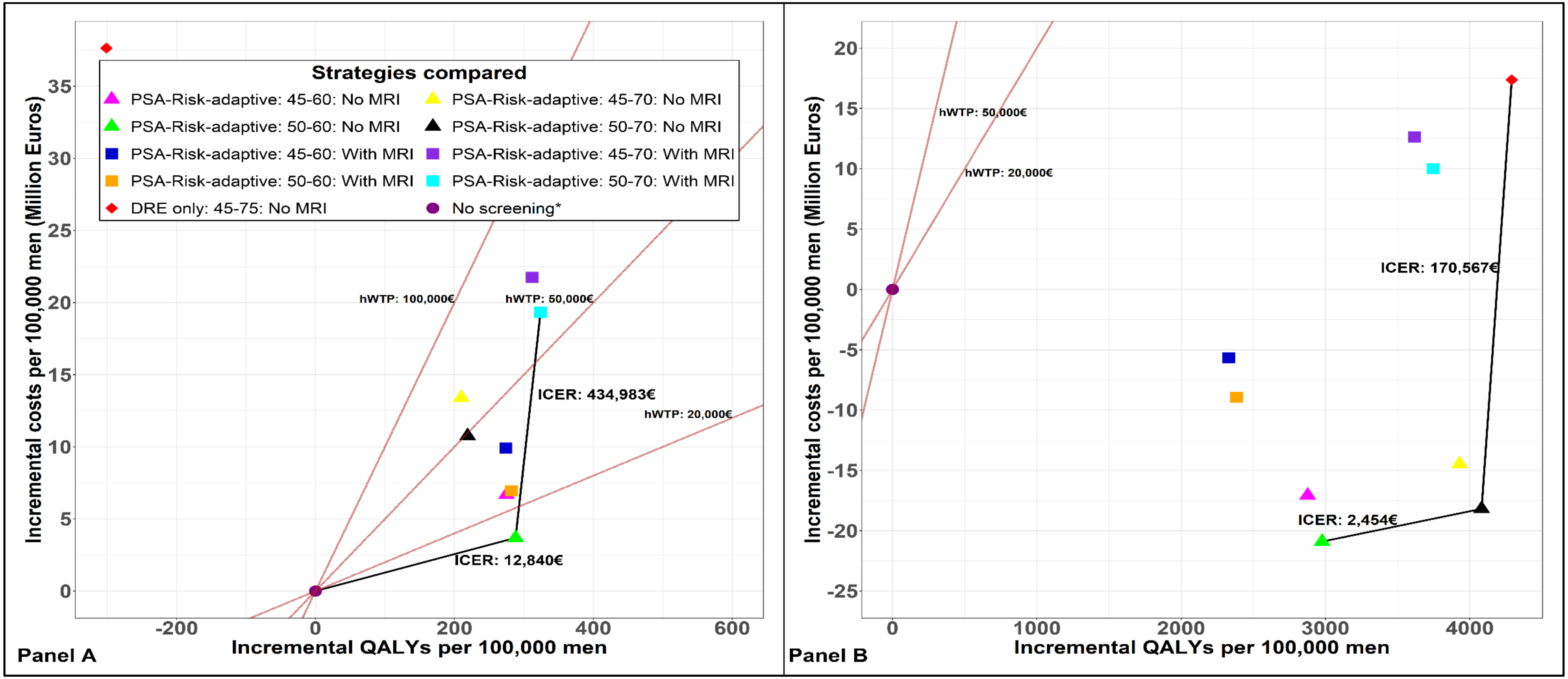

Figure S.11: DSA: Impact of varying the discount rates on cost and QALYs

Panel A: Discounting using 5% for both cost and health outcomes. **Panel B**: No discounting for both cost and health

DRE, digital rectal examination; with MRI means MRI is used to inform biopsy decisions followed by combined systemic (SBx) and targeted (TBx) biopsies. Strategies without MRI, “No MRI”, do not include MRI use to triage biopsy decision, suspicious cases are followed by systemic biopsies. *In the base case analysis, all symptomatically detected cancers were assumed to be further investigated using MRI imaging followed by combined biopsies, the red dotted lines represent the various hypothetical willingness to pay threshold (hWTP) used as the decision rule to interpret the cost-effectiveness ratios.

Source: Author’s elaboration

7.1.6 Impact of treatment patterns

Treatment distributions in economic modelling heavily influence costs, health outcomes, and overall results. Prostate cancer treatment approaches vary significantly due to differences in national guidelines, healthcare systems, professional practices, and cultural attitudes. A 2022 ASCO symposium survey highlighted these variations: Germany favours radical prostatectomy (71%), the UK prioritises radiotherapy (48%), and the USA leans towards prostatectomy (57%)[4].

Our base-case analysis followed clinical guidelines recommending conservative management, such as Active Surveillance or Watchful Waiting, for low-grade cancers or patients with limited life expectancy. These approaches reduce adverse events, preserve health-related quality of life (modelled as health state utility values), and lower costs. Radical treatments like prostatectomy or radiotherapy, while potentially curative, often lead to significant short- and long-term side effects, impairing quality of life.

Real-world data from the Saarland cancer registry corroborates Germany's preference for radical treatments. Table S.15 shows treatment distributions by Gleason score, informing our sensitivity analysis on the impact of real-world practices on model outcomes

Table S.15: Treatment Distribution by Gleason Score from the Saarland Registry, 2014-2019

Age	Gleason score	Conservative management	Radical prostatectomy	Radiation therapy
50-59	≤6	0,18	0,65	0,17
50-59	=7	0,11	0,82	0,07
50-59	≥8	0,33	0,65	0,03
60-69	≤6	0,27	0,50	0,23
60-69	=7	0,13	0,78	0,10
60-69	≥8	0,38	0,56	0,06
70-79	≤6	0,36	0,39	0,25
70-79	=7	0,31	0,51	0,18
70-79	≥8	0,53	0,40	0,07
≥80	≤6	0,54	0,33	0,13
≥80	=7	0,57	0,26	0,18
≥80	≥8	0,73	0,20	0,07

In comparison to the base case scenario in Figure 2 of the main article, Figure S.12 shows DSA results using actual treatment distributions observed from the Saarland cancer registry instead of the recommended treatment guidelines. From Figure 2, Figure S.12 shifts slightly upwards on the y-axis, demonstrating moderate increases in healthcare expenditures, discounted at 3%, for almost the sane health gains.

[4] https://www.dana-farber.org/newsroom/news-releases/2022/dana-farber-researchers-to-present-findings-at-2022-asco-genitourinary-cancers-symposium (Accessed 18.02.2024)

Specifically, the total healthcare costs for PSA risk-adapted screening, starting between ages 50-60 and followed by standard biopsy, escalated from saving approximately €1,2 million incremental cost of €1,187 million per 100,000 men screened compared to no screening.

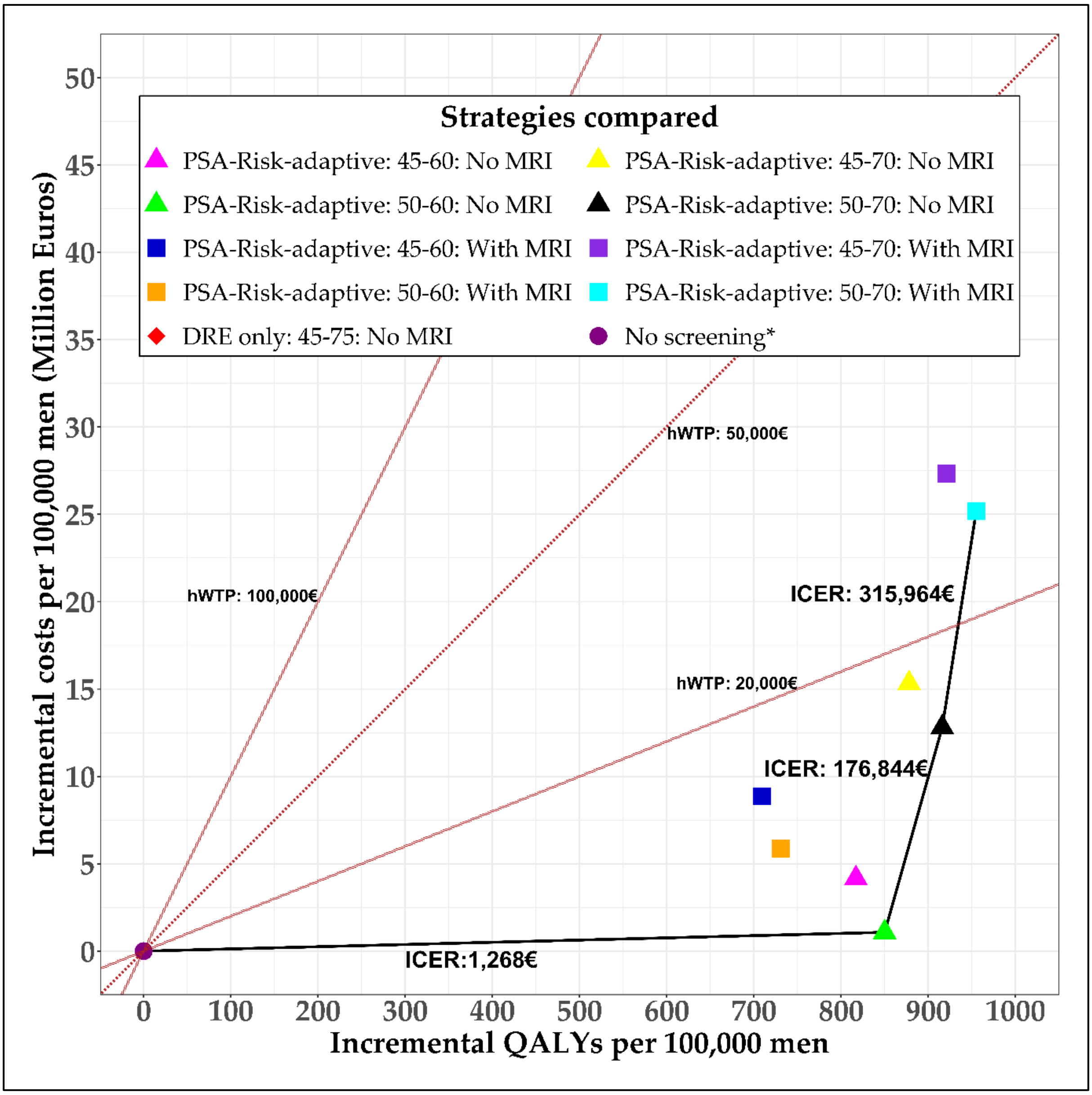

Figure S.12: DSA: Impact of using observed treatment distributions form the Saarland registry.
Notably, the MRI based interventions remain dominated
DRE, digital rectal examination; with MRI means MRI is used to inform biopsy decisions followed by combined systemic (SBx) and targeted (TBx) biopsies. Strategies without MRI, "No MRI", do not include MRI use to triage biopsy decision, suspicious cases are followed by systemic biopsies. *In the base case analysis, all symptomatically detected cancers were assumed to be further investigated using MRI imaging followed by combined biopsies, the red dotted lines represent the various hypothetical willingness to pay threshold (hWTP) used as the decision rule to interpret the cost-effectiveness ratios
Source: Author's elaboration

7.1.7 Impact reducing MRI cost from €500

Expert input from the PROBASE trial suggests MRI costs, in which they were funded approximately €400 per scan, could decrease further in a screening context due to economies of scale. Increased demand for MRI as part of structured screening protocols may drive prices down. Additionally, evidence supports the clinical utility of bpMRI, which is faster and less expensive than mpMRI, with no compromise in diagnostic quality. Transitioning to bpMRI alongside increased demand could further lower MRI costs. Two scenarios were considered: a conservative 50% reduction and a best-case scenario with costs as low as €120.

Results for the best-case scenario are shown in Figure 2 of the main manuscript. Figure S.13 demonstrates that reducing MRI costs to €250 improves the ICER for PSA risk-adaptive screening with MRI from €290,000/QALY to €93,687/QALY. However, reduced costs also decrease the overall expense of no screening. As a result, PSA risk-adaptive screening (50–60 years) without MRI is no longer cost-saving, with an ICER of €1,384/QALY. Further cost reductions make this strategy less favourable.

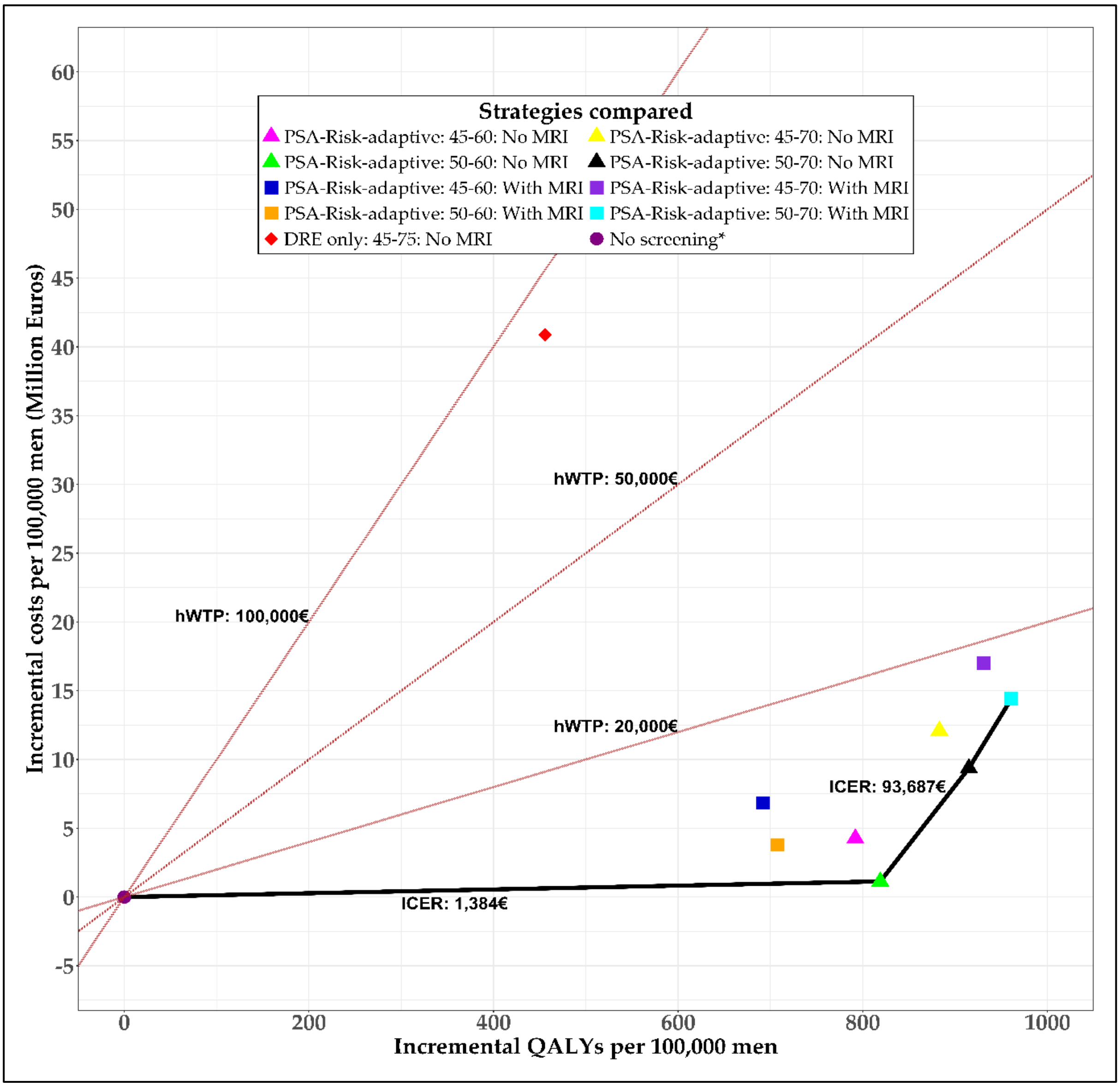

Figure S.13: DSA: Impact of reducing MRI cost by 50%
(see Figure S.12 for legend descriptions)

7.1.8 Summary of the results of the DSA

Figure S.14 juxtaposes 10 base case strategies with selected variations investigated PSA risk-adaptive screening initiated at ages 45, 50, and 55, and concluding at 60, 65, and 70. Screening intervals are set at 2 years for moderate-risk groups and 5 years for low-risk groups, aligning with current German recommendations [38, 39] and the EU's PRAISE-U initiative [40]. A biopsy threshold of 3 ng/mL was used for most strategies to conform to these guidelines and recommendations.

The findings suggest that PSA risk-adaptive screening starting at ages 50 to 60 without MRI is optimal under a €20,000 per QALY threshold. Extending PSA risk-adaptive screening without MRI to age 70 resulted in significant QALY gains but incurred considerable incremental costs. This strategy appears cost-effective with an ICER of €80,583 per QALY gained compared to PSA risk-adaptive screening (50–60) without MRI, suggesting its viability for those willing to invest more in health interventions. Similarly, PSA risk-adaptive screening (50–70) with MRI achieved the highest QALY gains and is now on the efficient frontier, appearing cost-effective with an ICER of €290,344 per QALY gained compared to PSA risk-adaptive screening (50–70) without MRI. Strategies initiating screening at 55 and stopping at 65 or 69, despite risk stratification and base case rescreening intervals, remain dominated.

Speculation that MRI costs could be reduced within the screening framework, combined with additional risk-scoring methods to triage MRI use, warrants further exploration to determine the most cost-effective integration of MRI within the screening context. We explored the impact of reducing cost to of MRI to 250€ (Figure S. 15) and 120€ (Figure 3, Panel B of the main manuscript text).

Figure S.15 presents the results of a probabilistic analysis, assuming a reduced mean MRI cost of €120 to reflect potential cost reductions. In this scenario, PSA-RAS (50–60 years) without MRI retains the highest probability of cost-effectiveness at WTP thresholds below €75,000 per QALY. Notably, the likelihood of PSA-RAS (50–70 years) being cost-effective increases significantly, exceeding a 60% probability at WTP thresholds below €100,000 per QALY

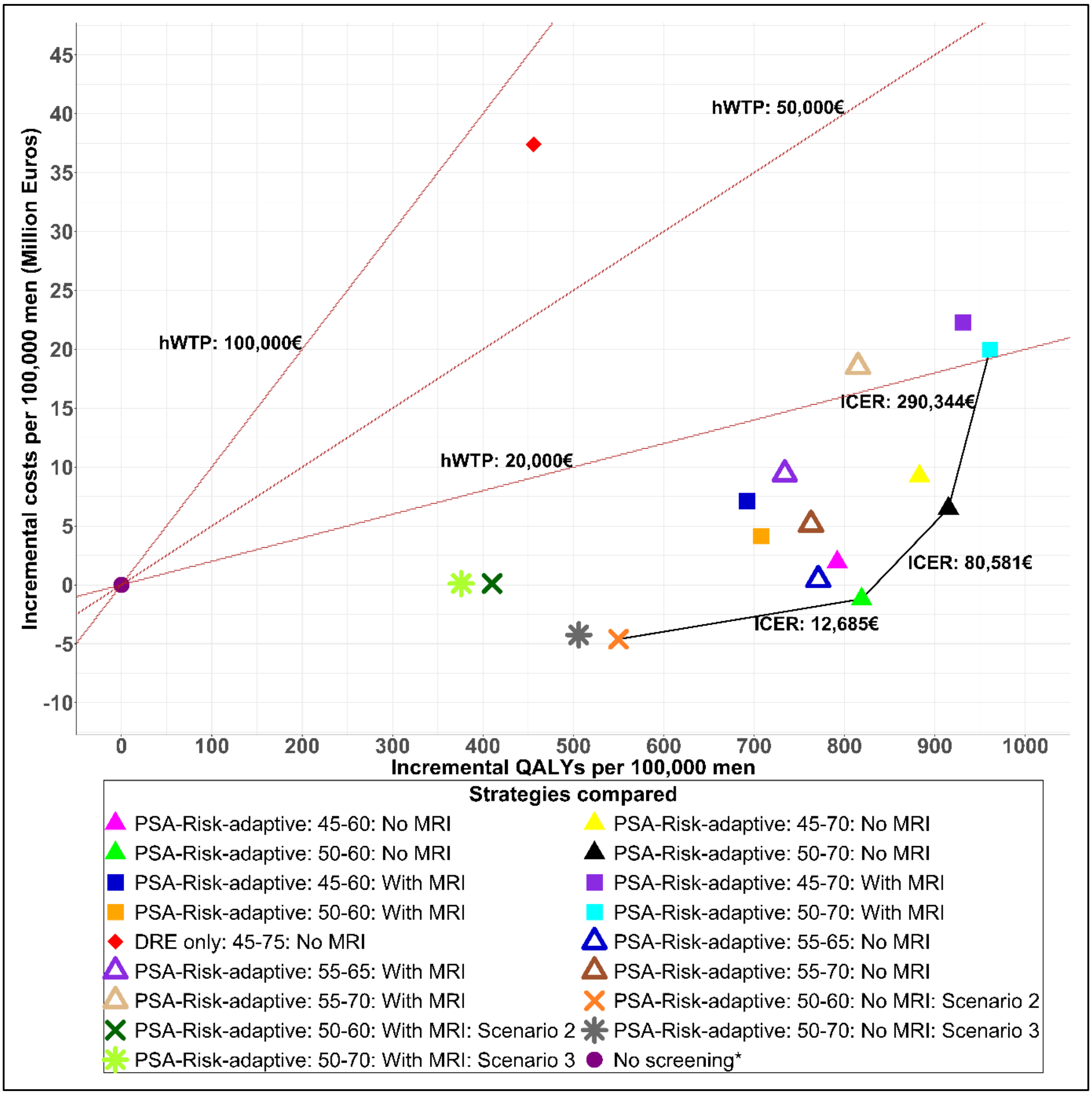

Figure S.14: DSA: Ten base case strategies compared with selected variations during the DSA
MRI, magnetic resonance imaging; PSA, prostate specific antigen; QALYs, quality-adjusted life-years; DRE, digital rectal examination; with MRI means MRI is used to inform biopsy decisions followed by combined systemic (SBx) and targeted (TBx) biopsies. Strategies without MRI, "No MRI", do not include MRI use to triage biopsy decision, suspicious cases are followed by systemic biopsies; DRE, Digital rectal examination In scenario 1, screening was confined to ages 55 to 70, with modifications across all strategies to exclude re-screening for low PSA risk groups (<2ng/ml) and implemented 5-year re-screening for moderate (2ng/ml<PSA<4ng/ml) and high-risk groups (PSA ≥ 4ng/ml) following negative MRI and biopsy results, up to age 70. The biopsy threshold was set at a PSA of 4ng/ml. Notably, PSA-risk-adaptive without MRI have been combined into a unified strategy, as have PSA-risk-adaptive with MRI. In scenario 2, screening was confined to age 70, with modifications across all strategies to exclude re-screening for low PSA risk groups (<1.5ng/ml) and implemented 5-year re-screening for moderate (1.5<PSA<3ng/ml) and high-risk groups (PSA ≥ 3ng/ml) following negative MRI and biopsy results, up to age 70. The biopsy threshold was set at a PSA of 3ng/ml, the red dotted lines represent the various hypothetical willingness to pay threshold (hWTP) used as the decision rule to interpret the cost-effectiveness ratios
Source: Author's elaboration

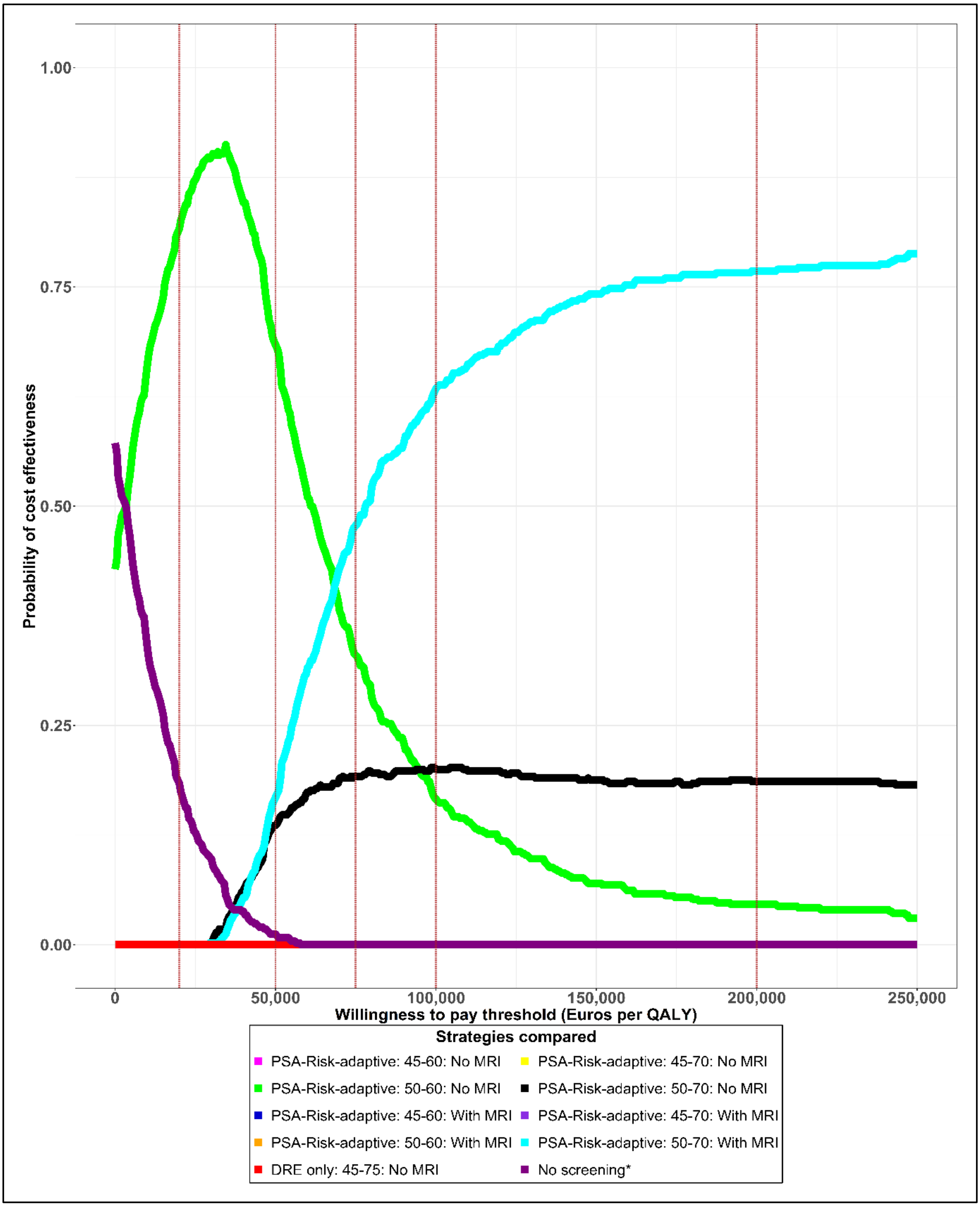

Figure S.15: CEAC: Results of probabilistic sensitivity analysis: mean MRI cost=120€

All 10 strategies—lines not appearing on the curve exhibited zero probability of being cost-effective within the willingness to pay threshold displayed: Horizontal red dashed line indicates the arbitrary cost-effectiveness threshold (k) of 20,000€; 50,000€, 75,0000€, 100,000€, and 200,000€ per QALY gained. MRI: magnetic resonance imaging; PSA: prostate specific antigen; QALYs: quality-adjusted life-years; DRE: digital rectal examination; with MRI means MRI is used to inform biopsy decisions followed by combined systemic (SBx) and targeted (TBx) biopsies. Strategies without MRI, "No MRI", do not include MRI use to triage biopsy decision, suspicious cases are followed by systemic biopsies; *In the base case analysis, all symptomatically detected cancers were assumed to be further investigated using MRI imaging followed by combined biopsies

Source: Author's elaboration

Table S.16: Model outcomes by strategy from age 45: Using 100% screening participation rate

	No screening*	PSA-Risk-adaptive: 50-60: No MRI	PSA-Risk-adaptive: 45-60: No MRI	PSA-Risk-adaptive: 50-60: With MRI	PSA-Risk-adaptive: 50-70: No MRI	PSA-Risk-adaptive: 45-60: With MRI	PSA-Risk-adaptive: 45-70: No MRI	PSA-Risk-adaptive: 50-70: With MRI	PSA-Risk-adaptive: 45-70: With MRI	DRE only: 45-75: No MRI
**Outcomes per 100,000 men**										
Number of DRE test	0	0	0	0	0	0	0	0	0	2501396
Number of PSA tests	0	293111	379163	294727	489307	380692	565492	494805	570705	0
Number of MRI events	0	0	0	17977	0	17319	0	69040	65791	0
Total number biopsy	19672	32415	32050	21061	76316	21043	73721	28196	27828	208286
Number of clinically-initiated biopsies	19672	15455	15688	16520	10753	16689	11226	12205	12600	6773
Number of screen-initiated biopsies	0	16960	16362	4541	65563	4355	62495	15991	15229	201513
Number of diagnosed PCa	10474	10892	10870	10743	12127	10729	12046	11680	11617	13768
Number of clinically diagnosed PCa	10474	8249	8373	8804	5749	8892	5998	6498	6707	3612
Number of screen diagnosed PCa	0	2642	2497	1940	6379	1837	6047	5182	4910	10156
Number of Localised and G < 7	3628	3910	3894	3767	4676	3760	4626	4255	4221	5613
Number of Localised and G = 7	3416	3540	3534	3535	3899	3529	3873	3884	3860	4359
Number of Localised and G > 7	3430	3442	3442	3441	3552	3440	3546	3541	3536	3796
Number of metastatic PCa diagnosed	2018	1636	1656	1722	1160	1735	1203	1260	1297	710
Estimated overdiagnosed PCa	0	418	396	270	1653	256	1571	1206	1143	3293
Prostate cancer deaths	3657	3210	3230	3326	2793	3338	2836	2936	2971	2403
LY, undiscounted	3458500	3463714	3463545	3462574	3467129	3462487	3466784	3465876	3465608	3470301
LY, discounted at 3%	2091200	2093107	2093055	2092715	2094204	2092691	2094095	2093786	2093703	2095225
QALYs, undiscounted	3119139	3123105	3122969	3122320	3124586	3122246	3124371	3124137	3123960	3124858
QALYs, discounted at 3%	1912587	1913680	1913642	1913532	1913809	1913511	1913763	1913871	1913829	1913194
QALYs, discounted at 5%	1474640	1475024	1475007	1475018	1474933	1475006	1474920	1475073	1475057	1474238
**Costs (M€) per 100,000 men**										
Healthcare perspective, undiscounted	426	402	407	417	405	422	410	441	445	452
Healthcare perspective, discounted at 3%	166	166	170	173	176	177	180	193	196	217
Healthcare perspective, discounted at 5%	94	100	104	104	109	108	112	120	123	145
ICERs (€ per QALY): Compared with No screening	**Reference case**	45	4021	7369	8363	11837	11842	21371		84625
ICERs on Efficient Frontier (€ per QALY)	**First on Frontier**	45	Dominated	Dominated	79001	Dominated	Dominated	277838	Dominated	Dominated

Columns are arranged in order of increasing healthcare perspective cost discounted at 3%. PSA: prostate specific antigen; MRI: magnetic resonance imaging; DRE: digital rectal examination; PCa: prostate cancer; LY: life years; GS: Gleason score; ICER: incremental cost-effectiveness ratio; QALY. Quality adjusted life years

Table S.17: Model outcomes by strategy from age 45: Using 50% screening participation rate

	PSA-Risk-adaptive: 50-60: No MRI	PSA-Risk-adaptive: 45-60: No MRI	No screening*	PSA-Risk-adaptive: 50-60: With MRI	PSA-Risk-adaptive: 50-70: No MRI	PSA-Risk-adaptive: 45-60: With MRI	PSA-Risk-adaptive: 45-70: No MRI	PSA-Risk-adaptive: 50-70: With MRI	PSA-Risk-adaptive: 45-70: With MRI	DRE only: 45-75: No MRI
**Outcomes per 100,000 men**										
Number of DRE test	0	0	0	0	0	0	0	0	0	2501396
Number of PSA tests	146513	189534	0	147328	244605	190300	282703	247359	285315	0
Number of MRI events	0	0	0	8995	0	8664	0	34533	32916	0
Total number biopsy	26045	25864	19672	20368	47996	20357	46700	23936	23753	208286
Number of clinically-initiated biopsies	17561	17679	19672	18099	15209	18183	15443	15935	16132	6773
Number of screen-initiated biopsies	8484	8186	0	2270	32787	2175	31257	8001	7621	201513
Number of diagnosed PCa	10682	10671	10474	10607	11299	10601	11259	11076	11045	13768
Number of clinically diagnosed PCa	9359	9422	10474	9639	8107	9683	8231	8482	8587	3612
Number of screen diagnosed PCa	1322	1250	0	968	3192	918	3028	2593	2458	10156
Number of Localised and G < 7	3769	3761	3628	3697	4151	3694	4127	3942	3925	5613
Number of Localised and G = 7	3477	3474	3416	3474	3657	3472	3644	3649	3638	4359
Number of Localised and G > 7	3436	3436	3430	3436	3491	3435	3488	3485	3483	3796
Number of metastatic PCa diagnosed	1825	1835	2018	1869	1585	1875	1607	1636	1654	710
Estimated overdiagnosed PCa	208	198	0	134	825	127	785	602	571	3293
Prostate cancer deaths	3434	3444	3657	3492	3226	3497	3247	3298	3315	2403
LY, undiscounted	3461114	3461033	3458500	3460546	3462826	3460507	3462656	3462195	3462067	3470301
LY, discounted at 3%	2092156	2092131	2091200	2091960	2092707	2091950	2092653	2092495	2092456	2095225
QALYs, undiscounted	3121128	3121062	3119139	3120736	3121869	3120703	3121764	3121641	3121558	3124858
QALYs, discounted at 3%	1913135	1913117	1912587	1913062	1913200	1913052	1913178	1913230	1913210	1913194
QALYs, discounted at 5%	1474833	1474825	1474640	1474830	1474788	1474825	1474781	1474857	1474850	1474238
**Costs (M€) per 100,000 men**										
Healthcare perspective, undiscounted	408	411	426	416	410	419	412	430	432	452
Healthcare perspective, discounted at 3%	163	165	166	167	169	169	170	178	180	217
Healthcare perspective, discounted at 5%	95	97	94	97	100	99	102	106	108	145
ICERs (€ per QALY): Compared with No screening	Dominant	Dominated	**Reference case**	2722	4375	7101	7660	19314	22423	84625
ICERs on Efficient Frontier (€ per QALY)	**First on Frontier**	Dominated	Dominated	Dominated	79284	Dominated	Dominated	329414	Dominated	Dominated

Columns are arranged in order of increasing healthcare perspective cost discounted at 3%. PSA: prostate specific antigen; MRI: magnetic resonance imaging; DRE: digital rectal examination; PCa: prostate cancer; LY: life years; GS: Gleason score; ICER: incremental cost-effectiveness ratio; QALY. Quality adjusted life years

8 Supplementary references

1. Karlsson A, Jauhiainen A, Gulati R, Eklund M, Gronberg H, Etzioni R, et al. A natural history model for planning prostate cancer testing: Calibration and validation using Swedish registry data. PloS one. 2019;14(2):e0211918.
2. Loosen SH, Krieg S, Eschrich J, Luedde M, Krieg A, Schallenburger M, et al. The Landscape of Outpatient Palliative Care in Germany: Results from a Retrospective Analysis of 14,792 Patients. Int J Environ Res Public Health. 2022 Nov 12;19(22).
3. Winter A, Sirri E, Jansen L, Wawroschek F, Kieschke J, Castro FA, et al. Comparison of prostate cancer survival in Germany and the USA: can differences be attributed to differences in stage distributions? BJU Int. 2017 Apr;119(4):550-9.
4. Hao S, Östensson E, Eklund M, Grönberg H, Nordström T, Heintz E, et al. The economic burden of prostate cancer - a Swedish prevalence-based register study. BMC health services research. 2020 May 20;20(1):448.
5. Stadlbauer A, Bernt R, Salomonowitz E, Plas E, Strunk G, Eberhardt K. [Health economics evaluation of magnetic resonance imaging for the staging of prostate cancer for Austria and Germany]. RoFo : Fortschritte auf dem Gebiete der Rontgenstrahlen und der Nuklearmedizin. 2012 Jun;184(6):556-64.
6. Niklas C, Saar M, Berg B, Steiner K, Janssen M, Siemer S, et al. da Vinci and Open Radical Prostatectomy: Comparison of Clinical Outcomes and Analysis of Insurance Costs. Urologia internationalis. 2016;96(3):287-94.
7. Koerber F, Waidelich R, Stollenwerk B, Rogowski W. The cost-utility of open prostatectomy compared with active surveillance in early localised prostate cancer. BMC health services research. 2014 2014/04/10;14(1):163.
8. Reinhold T, Dornquast C, Börgermann C, Weißbach L. [Treatment costs of localized prostate cancer in Germany : Economic results from the HAROW observational study]. Der Urologe Ausg A. 2016 Dec;55(12):1573-85.
9. Kreis K, Horenkamp-Sonntag D, Schneider U, Zeidler J, Glaeske G, Weissbach L. Treatment-Related Healthcare Costs of Metastatic Castration-Resistant Prostate Cancer in Germany: A Claims Data Study. PharmacoEconomics - open. 2021 Jun;5(2):299-310.
10. Fourcade RO, Benedict A, Black LK, Stokes ME, Alcaraz A, Castro R. Treatment costs of prostate cancer in the first year after diagnosis: a short-term cost of illness study for France, Germany, Italy, Spain and the UK. BJU Int. 2010 Jan;105(1):49-56.
11. Roehrborn CG, Black LK. The economic burden of prostate cancer. BJU Int. 2011 Sep;108(6):806-13.
12. Govers TM, Hessels D, Vlaeminck-Guillem V, Schmitz-Dräger BJ, Stief CG, Martinez-Ballesteros C, et al. Cost-effectiveness of SelectMDx for prostate cancer in four European countries: a comparative modeling study. Prostate Cancer Prostatic Dis. 2019 Mar;22(1):101-9.

13. Schiffer E, Bick C, Grizelj B, Pietzker S, Schöfer W. Urinary proteome analysis for prostate cancer diagnosis: cost-effective application in routine clinical practice in Germany. Int J Urol. 2012 Feb;19(2):118-25.

14. Herkommer K, Fuchs TA, Hautmann RE, Volkmer BG. [Radical prostatectomy for men aged <56 years with prostate cancer. Cost of illness analysis]. Der Urologe Ausg A. 2005 Oct;44(10):1183-4, 5-8.

15. Michaeli T, Michaeli D. Prostate cancer follow-up costs in Germany from 2000 to 2015. Journal of cancer survivorship : research and practice. 2022 Feb;16(1):86-94.

16. Smith-Palmer J, Takizawa C, Valentine W. Literature review of the burden of prostate cancer in Germany, France, the United Kingdom and Canada. BMC Urol. 2019 Mar 18;19(1):19.

17. Wang L, Hong H, Alexander GC, Brawley OW, Paller CJ, Ballreich J. Cost-Effectiveness of Systemic Treatments for Metastatic Castration-Sensitive Prostate Cancer: An Economic Evaluation Based on Network Meta-Analysis. Value in health : the journal of the International Society for Pharmacoeconomics and Outcomes Research. 2022 May;25(5):796-802.

18. Garje R, Chennamadhavuni A, Mott SL, Chambers IM, Gellhaus P, Zakharia Y, et al. Utilization and Outcomes of Surgical Castration in Comparison to Medical Castration in Metastatic Prostate Cancer. Clinical genitourinary cancer. 2020 Apr;18(2):e157-e66.

19. Parker C, Castro E, Fizazi K, Heidenreich A, Ost P, Procopio G, et al. Prostate cancer: ESMO Clinical Practice Guidelines for diagnosis, treatment and follow-up. Annals of oncology : official journal of the European Society for Medical Oncology. 2020 Sep;31(9):1119-34.

20. Murgić J, Fröbe A, Challapalli A, Bahl A. ROLE OF ANDROGEN RECEPTOR-TARGETED AGENTS IN LOCALIZED PROSTATE CANCER. Acta Clin Croat. 2022 Oct;61(Suppl 3):51-6.

21. Barbier MC, Tomonaga Y, Menges D, Yebyo HG, Haile SR, Puhan MA, et al. Survival modelling and cost-effectiveness analysis of treatments for newly diagnosed metastatic hormone-sensitive prostate cancer. PloS one. 2022;17(11):e0277282.

22. Groeneveld EI, Cassel JB, Bausewein C, Csikós Á, Krajnik M, Ryan K, et al. Funding models in palliative care: Lessons from international experience. Palliative Medicine. 2017 Apr;31(4):296-305.

23. Radbruch L, De Lima L, Knaul F, Wenk R, Ali Z, Bhatnaghar S, et al. Redefining Palliative Care-A New Consensus-Based Definition. Journal of pain and symptom management. 2020 Oct;60(4):754-64.

24. Freytag A, Meissner F, Krause M, Lehmann T, Jansky MK, Marschall U, et al. Ergebnisqualität und Kosten der allgemeinen und spezialisierten Palliativversorgung in Deutschland im regionalen Vergleich: eine GKV-Routinedatenstudie. Bundesgesundheitsblatt - Gesundheitsforschung - Gesundheitsschutz. 2023 2023/08/03.

25. Ditscheid B, Meissner F, Gebel C, Hennig B, Marschall U, Meißner W, et al. Inanspruchnahme von Palliativversorgung am Lebensende in Deutschland: zeitlicher Verlauf (2016–2019) und regionale

Variabilität. Bundesgesundheitsblatt - Gesundheitsforschung - Gesundheitsschutz. 2023 2023/04/01;66(4):432-42.

26. Rusche H, Kreimendahl F, Huenges B, Becka D, Rychlik R. [Medical care and costs in the last year of life - propensity score matching of AAPV and SAPV insurants]. Dtsch Med Wochenschr. 2016 Oct;141(22):e203-e12.

27. Drummond MF, Sculpher MJ, Claxton K, Stoddart GL, Torrance GW, Drummond D. Methods for the economic evaluation of health care programmes. . Fourth ed: Oxford University Press; 2015.

28. Sanghera S, Coast J, Martin RM, Donovan JL, Mohiuddin S. Cost-effectiveness of prostate cancer screening: a systematic review of decision-analytical models. BMC cancer. 2018 Jan 18;18(1):84.

29. Heijnsdijk EA, de Carvalho TM, Auvinen A, Zappa M, Nelen V, Kwiatkowski M, et al. Cost-effectiveness of prostate cancer screening: a simulation study based on ERSPC data. Journal of the National Cancer Institute. 2015 Jan;107(1):366.

30. Ross KS, Guess HA, Carter HB. Estimation of treatment benefits when PSA screening for prostate cancer is discontinued at different ages. Urology. 2005 Nov;66(5):1038-42.

31. Howard DH. Life expectancy and the value of early detection. J Health Econ. 2005 Sep;24(5):891-906.

32. Zhang J, Denton BT, Balasubramanian H, Shah ND, Inman BA. Optimization of PSA screening policies: a comparison of the patient and societal perspectives. Medical decision making : an international journal of the Society for Medical Decision Making. 2012 Mar-Apr;32(2):337-49.

33. Hao S, Discacciati A, Eklund M, Heintz E, Östensson E, Elfström KM, et al. Cost-effectiveness of Prostate Cancer Screening Using Magnetic Resonance Imaging or Standard Biopsy Based on the STHLM3-MRI Study. JAMA oncology. 2022.

34. Hao S, Heintz E, Östensson E, Discacciati A, Jäderling F, Grönberg H, et al. Cost-Effectiveness of the Stockholm3 Test and Magnetic Resonance Imaging in Prostate Cancer Screening: A Microsimulation Study. European urology. 2022 Jul;82(1):12-9.

35. Hao S, Karlsson A, Heintz E, Elfström KM, Nordström T, Clements M. Cost-Effectiveness of Magnetic Resonance Imaging in Prostate Cancer Screening: A Microsimulation Study. Value in health : the journal of the International Society for Pharmacoeconomics and Outcomes Research. 2021 Dec;24(12):1763-72.

36. Heijnsdijk EAM, de Koning HJ. Wise Prostate-specific Antigen Testing Means a Limited, Risk-adjusted, and Personal Approach. European urology. 2023 Jun 7.

37. Magnus A, Isaranuwatchai W, Mihalopoulos C, Brown V, Carter R. A Systematic Review and Meta-Analysis of Prostate Cancer Utility Values of Patients and Partners Between 2007 and 2016. MDM policy & practice. 2019 Jan-Jun;4(1):2381468319852332.

38. Arsov C, Becker N, Hadaschik BA, Hohenfellner M, Herkommer K, Gschwend JE, et al. Prospective randomized evaluation of risk-adapted prostate-specific antigen screening in young men: the PROBASE trial. European urology. 2013 Dec;64(6):873-5.

39. Michel MS, Gschwend JE, Wullich B, Krege S, Bolenz C, Merseburger AS, et al. Risikoadaptierte Prostatakarzinomfrüherkennung 2.0 – Positionspapier der Deutschen Gesellschaft für Urologie 2024. Die Urologie. 2024 2024/09/01;63(9):893-8.

40. Chandran A, van Harten M, Singh D, Vilaseca J, Patasius A, Tupikowski K, et al. Risk-stratified Approach to Implementing Population-based Prostate Cancer Screening in Five Pilot Sites in the European Union: A Protocol for the PRAISE-U Project. European Urology Open Science. 2024 2024/12/01/;70:8-17.
